# Experiences Receiving and Delivering Virtual Health Care For Women: Qualitative Evidence Synthesis

**DOI:** 10.2196/68314

**Published:** 2025-05-15

**Authors:** Karen M Goldstein, Sharron Rushton, Allison A Lewinski, Abigail Shapiro, Tiera Lanford-Davey, Jessica N Coleman, Neetu Chawla, Dhara B Patel, Katherine Van Loon, Megan Shepherd-Banigan, Catherine Sims, Sarah Cantrell, Susan Alton Dailey, Jennifer M Gierisch

**Affiliations:** 1 VA Center of Innovation to Accelerate Discovery and Practice Transformation Durham, NC United States; 2 Division of General Internal Medicine Duke University School of Medicine Durham, NC United States; 3 Duke University School of Nursing Durham, NC United States; 4 VA Center for the Study of Healthcare Innovation Implementation and Policy Los Angles, CA United States; 5 Department of Population Health Sciences Duke University School of Medicine Durham, NC United States; 6 Duke Margolis Institute for Health Policy Durham, NC United States; 7 VA Veterans Integrated Services Network-6 Mid-Atlantic Mental Illness Research and Education Clinical Center Durham, NC United States; 8 Division of Rheumatology Duke University School of Medicine Durham, NC United States; 9 Duke University Medical Center Library & Archives Duke University School of Medicine Durham, NC United States

**Keywords:** virtual care, telehealth, women’s health, sex-specific care, access to care

## Abstract

**Background:**

Persisting sex- and gender-based disparities in access to high-quality, personalized health care in the United States can lead to devastating outcomes with long-lasting consequences. Strategic use of virtual resources could expand equitable health care access for women. However, optimal approaches and timing for individualized, virtually delivered health care for women are unclear.

**Objective:**

This study aims to conduct a detailed analysis of the current literature to answer the following question: “According to women and their health care teams, what are the reported successes and challenges in accessing, delivering, and participating in synchronous virtual health care for women?”

**Methods:**

We conducted a qualitative evidence synthesis using a best-fit framework approach based on the Nonadoption, Abandonment, Scale-up, Spread, and Sustainability (NASSS) framework and concepts from the Public Health Critical Race Praxis. We searched MEDLINE, Embase, and CINAHL from January 1, 2010, to October 10, 2022, using a combination of database-specific, relevant, controlled vocabulary terms and keywords; this search was updated in MEDLINE through January 2024. Additional citations were identified through handsearching. Our eligibility criteria were developed using the Sample, Phenomenon of Interest, Design, Evaluation, Research type tool to identify qualitative studies addressing synchronous virtual care for women. Citations were screened in duplicate, and eligible articles were abstracted. An iterative thematic synthesis approach was used to identify descriptive themes related to the successes and challenges related to delivering high-quality virtual care. Data reduction was performed using inductive and deductive reasoning. Quality assessment was conducted using the Critical Appraisal Skills Program and certainty of evidence using Confidence in the Evidence from Reviews of Qualitative Research approaches.

**Results:**

Of 85 eligible articles, we sampled 51 (60%) for data extraction based on representation of patient and clinician perspectives, marginalized voices, and relevance to a variety of clinical contexts. We identified themes across NASSS domains, including difficulty building rapport and emotional connections in the virtual setting, the amplification of barriers for women with preexisting challenges (eg, language barriers, limited transportation, and family and social commitments), and differing perceptions of privacy and safety related to virtual care depending on patient home context. Themes found to have high confidence included the value of convenience and cost savings offered by virtual care, the importance of patient choice in visit modality, the potential for negative impact on user well-being, considering the clinical context of modality choice, the importance of technology usability, and the value of virtual care for women located in regions without adequate supply of clinical offerings.

**Conclusions:**

The benefits of virtual care for health care access may be more acutely felt by women, especially those with preexisting challenges. Strategic incorporation of virtual modalities into health care delivery for women could improve equitable access to high quality, patient-centered care.

**Trial Registration:**

PROSPERO CRD42021283791; https://www.crd.york.ac.uk/PROSPERO/view/CRD42021283791

**International Registered Report Identifier (IRRID):**

RR2-10.1089/heq.2023.0089

## Introduction

### Background

Sex- and gender-based disparities in access to high-quality, personalized health care persist in the United States. Compared with men, women have more limited access to a regular source of care, fewer feasible choices in health care source [1-3], as well as higher associated health care costs and greater competing domestic demands (eg, dependent care provision) [4-6]. In addition, trauma-related experiences, which are more common among women [7], are associated with a preference for gender-concordant clinicians [8] and the need to establish physical and emotional safety in health care settings [9]. Moreover, women often experience missed or delayed diagnoses with devastating long-term adverse health outcomes [1-3]. For example, although women use outpatient care more often than men and experience higher rates of chronic conditions and disability [4], women more frequently report delayed receipt of care for high-risk conditions, such as diabetes and heart disease [2]. In addition, women are significantly more likely than men to receive poor quality care for acute coronary syndromes and have worse outcomes following cardiac ischemic events [10,11].

Barriers to care are often amplified among women from populations considered historically marginalized and those residing in areas with physical barriers, such as distance to health care facilities or limited transportation. In the United States, people of color have less regular access to a source of care compared to White individuals [12], while barriers to affordable care are exacerbated for women in regions with structural sexism or gender-based inequitable distribution of power and resources [3]. Many women experience multiplicative barriers from structural bias related to other aspects of their identity, such as having a chronic condition, single motherhood, or disability. Women with such barriers to care warrant targeted strategies to improve access to care.

Leveraging virtual visits as a purposeful component of health care delivery may be a promising strategy for improving equitable care access and addressing health care needs for women. While expanding access to care for all patients, virtual care delivery may be uniquely valuable to women who may benefit from flexible, virtually delivered innovations. As demonstrated during the COVID-19 pandemic, women are more receptive to video-based care [13], the use of which was not impacted by age, rurality, mental health, or marital status, unlike use patterns among men [14]. Women are also more likely to choose virtual care visits over in-person when seeing a known clinician [14-16]. Importantly, virtual health care services designed to account for sex- or gender-based barriers and to address systemic racism and discrimination may improve these intersectional health disparities for women [17]. Thus, it is important to explore how to optimize the deployment of virtual health care modalities informed by conceptual and theoretical approaches that purposefully attend to, and address, the perspectives and needs of individuals who often struggle with access to care. Strategic integration of virtual care, such as videoconferencing and telephone visits, into high-quality, patient-centered health care for women may therefore improve sex- and gender-based disparities and equitable access to care.

We do not know how to deploy virtual care to optimize health care for women. To date, there has been no rigorous, comprehensive evidence-based summary of how virtual care should be optimized to improve health care provision for women. Existing guidance on the conduct of synchronous virtual care does not specifically address the adaptation of these approaches to the needs of women or is largely based on expert opinion alone [18-21]. It is particularly unclear how to best use virtual care for women living in lower-resourced areas or with preexisting limited access to health care [22].

### This Study

To address this information gap, we conducted a systematic review of qualitative literature to synthesize the reported barriers and successes experienced by both women who received virtual care and the health care teams who provided it. We opted to use qualitative evidence synthesis methodology as this approach is optimal for assessing contextual factors related to the complexity of individual experiences of a phenomenon and *how* that experience could be improved. To ensure our analysis and findings are relevant to individuals with the greatest health care access barriers, we will incorporate key concepts from relevant implementation science and equity-focused frameworks. Our key question was as follows: “What do women and health care teams report are successes and challenges to accessing, delivering, and engaging in synchronous virtual health care for women?”

## Methods

### Overview

We used a qualitative evidence synthesis approach to assess the existing literature describing experiences of women using virtual care [23]. Due to the historical dearth of representative gender diversity within published literature, our synthesis aimed to include the experiences of all individuals who might use care defined under the umbrella of “women’s health” or those who might use the identifiers *woman* or *female*. To ensure accurate reflection of the identified literature, we used the term *woman* to reflect any individual who identified as a woman (ie, cis-gender woman and trans woman) or who was identified anatomically as female at birth and does not identify as a woman (eg, trans man and gender nonbinary). We recognize that this approach does not encapsulate the identity of all virtual care users and that both biology and sociocultural experiences can uniquely impact health care needs and barriers. Our focus was on synchronous virtual care or health care visits conducted via a telephone or video platform for the real-time exchange of bidirectional information between patients and clinicians for the purposes of providing individual care. We categorized pathways of care as being related to primary care, mental health, and specialty care.

### Positionality Statement

Our approach was largely informed by the clinical and research experiences of the first and senior authors, both of whom are cisgender White women with terminal degrees. The identities of our author team also informed our approach to this evidence synthesis, including 3 women of color; lesbian, gay, bisexual, transgender, queer (LGBTQ) authors; as well as representation of disability. Most team members have experience with Public Health Critical Race Praxis (PHCRP) and are interdisciplinary, representing public health, medicine, nursing, psychology, and health behavior disciplines. All authors have experienced virtual care as patients, and most clinician authors have provided virtual care. We consulted with, and incorporated input from, our patient researcher team member at multiple points during the conduct of this study. While we intentionally sought to include diversity of voices on our team to strengthen the rigor of this work and assist with centering this work on voices from populations considered marginalized, we recognize the incomplete nature of inclusion on our team and how this might impact our review. We also acknowledge how our own implicit biases might hinder our ability to perceive certain PHCRP constructs in data, such as the ordinariness of racism, with a majority White team.

### Guiding Framework

As detailed previously, our work is guided by 2 existing models [24]. First, to ensure focus on equity and inclusion of the perspective and needs of nonprivileged groups, our methods are guided by PHCRP [25]. Specifically, our work is informed by the PHCRP’s orientation of centering on the margins and emphasizing perspectives of groups considered socially marginalized (ie, women of color) rather than the dominant cultural group (ie, White women). Second, our work is guided by the Nonadoption, Abandonment, Scale-up, Spread, and Sustainability (NASSS) technology implementation framework as it provides a way to understand the implementation of technology in the context of health care delivery [26]. The NASSS includes seven domains: (1) health condition, (2) technology, (3) value proposition, (4) adopter system, (5) organization, (6) wider (institutional and societal) context, and (7) embedding and adaptation over time. The NASSS was used as the starting point for a modified best-fit framework approach to synthesize across identified literature. For the assessment of each NASSS domain, we incorporated specific PHCRP domains (eg, structural determinism and ordinariness of racism) into our a priori coding scheme used with each included article. Then, while summarizing and assessing themes within each NASSS domain, we considered the overlap of data coded using PHCRP domains.

### Search Strategy

We developed an a priori protocol for this review, which was registered with PROSPERO (CRD42021283791) and described in detail previously [24]. We searched the following databases from January 1, 2010, to October 10, 2022, using a combination of database-specific, controlled vocabulary terms and keywords: MEDLINE (through Ovid), Embase (through Elsevier), and CINAHL Complete (through EBSCO; Multimedia Appendix 1). Additional citations were identified by handsearching relevant reviews and references, as well as identification from telehealth listserves. To ensure the identification of current literature, we updated this search in MEDLINE using the same search strategy through January 5, 2024, to identify additional concepts or experiences from literature published since our initial search. We screened additional citations identified in January 2024, as described earlier. We abstracted the results and combined them with our existing synthesis, as described subsequently. Our search strategy was developed and implemented by an expert medical librarian, with input from the review team and a second librarian during peer review. Citations were managed in Covidence Systematic Review Software (Veritas Health Innovation) and EndNote (Clarivate Analytics).

### Eligibility Criteria and Screening

Our eligibility criteria were developed using the Sample, Phenomenon of Interest, Design, Evaluation, Research type tool to identify primary qualitative studies (Multimedia Appendix 2) [27]. Studies that collected data synchronously were eligible (ie, not via asynchronous survey). We restricted articles to those relevant to care provided in the United States or countries with similar economic and health care resources because we intend for our findings to generalize to US-based health care systems. We excluded studies that evaluated asynchronous virtual care (ie, SMS text messaging and web-based portals) and those that included both men and women, which did not report results specific to women separately.

We screened articles for eligibility by title and abstract and subsequently by full text in duplicate. Each step was piloted. We resolved eligibility conflicts via team discussion or arbitration by a third team member. All articles identified for inclusion were labeled according to established health care delivery pathways (ie, mental health, primary care, and specialty care) and our a priori identified focus on populations considered historically marginalized (ie, rural dwelling, women of color, veteran status, and sexual- or gender-marginalized groups). Each article was assigned a richness score by 2 team members (AS and TL-D), ranging from 1 to 5, based on the volume and depth of relevant qualitative data [28]. We sampled a subset of articles for abstraction with a focus on overrepresenting marginalized voices, purposively choosing articles from each health care delivery pathway, and prioritizing articles with higher richness scores.

### Data Abstraction and Synthesis

We used a best-fit framework approach for abstraction and synthesis of narrative data [29] using thematic synthesis based on NASSS domains, as described earlier. Study characteristics were abstracted in Covidence by a single reviewer and were overread by a second reviewer, with discrepancies resolved via discussion (Multimedia Appendix 3). We piloted our qualitative coding approach to ensure calibration and then coded narrative data from the included studies into NASSS domains using Microsoft Excel. A subgroup of 3 investigators (KMG, AAL, and SR) subsequently reduced data into analytic themes using inductive and deductive reasoning [30], to promote rigor and transparency. As we found stability of data across themes, suggesting saturation from initial sampling strata, we did not sample additional articles from our eligible pool [31].

### Quality Assessment

Assessment of an individual study’s methodological limitations or quality supports an understanding of how likely its results reflect the actual phenomena under study without systematic bias. We used the Critical Appraisal Skills Program tool for qualitative studies to assess sources of potential bias in the included studies [32]. Critical Appraisal Skills Program considers the validity of results, appropriateness and rigor of methods used, clarity of stated aims and conclusions, consideration of ethical issues, and the relationship between researcher and participant. Quality assessments were completed in duplicate by 2 independent investigators, with discrepancies resolved through discussion.

### Certainty of Evidence

A key step for a systematic review is assessing the certainty of evidence or determining the degree to which the available evidence accurately addresses the guiding questions for the review. To select the findings most important to our research questions, we sought guidance from a multiperspective advisory panel, a study-specific advisory group composed of individuals with experiences receiving, delivering, or overseeing the care of women [24]. The multiperspective advisory panel prioritized the following NASSS domains: value proposition, adopter system, and wider context. To facilitate transparency in our certainty of evidence assessment, we used the GRADE-Confidence in the Evidence from Reviews of Qualitative Research web-based tool [33], which considers the methodological limitations of contributing literature, coherence between the findings and primary data, adequacy of contributing data, and relevance of contributing data to the intended context. A subgroup of investigators with expertise in systematic reviews and who were most heavily involved with data synthesis made Confidence in the Evidence from Reviews of Qualitative Research assessments as a group (KMG, AAL, and SR). This work was approved by the Durham Department of Veteran Affairs Institutional Review Board.

## Results

### Overview of the Included Studies

**Figure 1:**
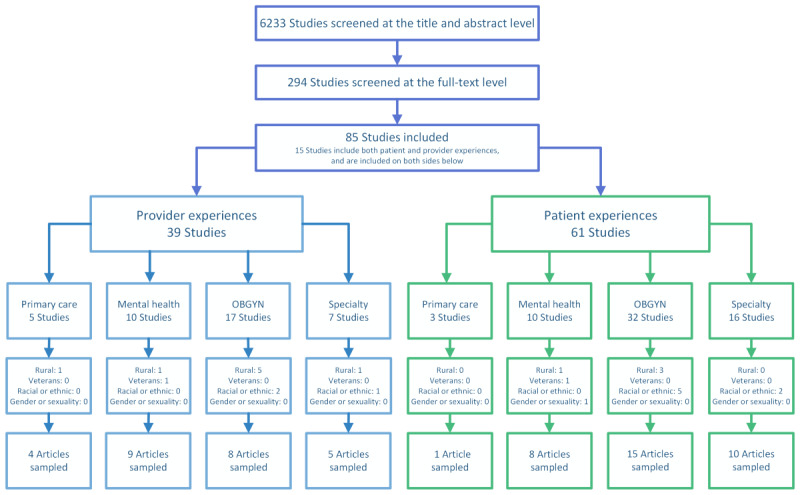
Literature flow diagram. OBGYN: obstetrics and gynecology.

We identified 85 eligible articles, of which 51 (60%) were sampled for data abstraction. Figure 1 shows our organization of included studies and from which categories the extracted studies were sampled. Of the 51 sampled articles, we found that 25 (49%) articles represented data from exclusively patient perspectives [34-58], of which 1 (4%) was relevant to primary care [34], 5 (20%) to mental health [35-39], and 19 (76%) to specialty care (including obstetrics and gynecology) [40-58]. Of the 51 articles, 17 (33%) represented only clinician perspectives [59-75], with 4 (8%) relevant to primary care [59-62], 6 (12%) to mental health [49,63-68,76-78], and 7 (14%) to specialty care (which included obstetrics and gynecology) [69-71,73-75,79]. Of the 51 articles, 9 (18%) included both patient and clinician data, with 3 (6%) relevant to mental health [76-78] and 6 (12%) to specialty care [72,80-84]. Details about excluded studies can be found in Multimedia Appendix 4.

Among articles addressing the primary care setting, common clinical conditions or care types were contraceptive and preexposure prophylaxis care. Among those addressing mental health, common clinical foci were intimate partner violence or domestic violence interventions, prenatal behavioral health, and mindfulness. Those focused on specialty care most often focused on abortion, breast and gynecological cancer, prenatal care, and tele-lactation. Of the 51 articles, 4 (8%) focused on women considered racially or ethnically marginalized [40,49,55,84]; 1 (2%) on lesbian, gay, bisexual, transgender, queer, and similar minority (LGBTQ+) individuals [35]; and 7 (14%) on rural-dwelling women [39,41,61,63,75,81,84]. An explicit focus on experiences with video-based care was reported by 25 articles [35-39,43,47,49,52,54-56,58,63,68,71,72,75-79,81,82,84,85], while 10 articles focused on telephone-based care [42,45,46,50,51,69,70,73,80,83] and 13 focused on both [34,40,44,48,53,57,59-61,64,66,67,74]. Of the 51 articles, 3 (6%) did not specify the type of care [41,62,65]. Of the 51 articles, 5 (10%) addressed group-based care [35-39]. Refer to Multimedia Appendix 5 for individual study characteristics.

Common sources of potential bias included an absent description of theoretical underpinnings for the study and limited or no discussion of researcher-participant relationships. We also identified some articles with potential biases related to recruitment strategies, data collection, and analysis rigor. Details on risk of bias assessments are provided in Multimedia Appendix 6.

### NASSS Domains

#### Overview

Findings are organized by NASSS domains. For domains prioritized for certainty of evidence, we report our assessments in the context of each finding. A full description of the assessment is provided in Multimedia Appendix 7. Details and illustrative quotes are provided in Multimedia Appendix 8.

#### Technology

The technology domain pertains to aspects of the physical hardware and software used by patients and clinicians while engaging in virtual care and the impact of this technology on clinical relationships and experience. Key themes in this domain included the challenging impact that virtual care has on establishing and leveraging emotional connections between patients and clinicians, the need for training both patients and clinicians to promote engagement during virtual visits, the risk of amplifying communication barriers during virtual visits, and the importance of matching modality to clinical context of each visit. Key factors associated with the uptake of virtual care included the usability of available technology and related platforms and preexisting infrastructure and experience.

#### Adopter System

The adopter system addressed the features of the individuals using technology (ie, women and their clinicians) that impacted the uptake of virtual modalities for health care. Themes related to virtual care adopters included an acknowledgment of the negative impact on both patient and clinician well-being, the transferability of clinicians’ existing interpersonal and technological skills to the virtual setting, and the varying effects of the virtual care environment on patient privacy and safety, both positive and negative.

#### Value Proposition

The value proposition domain acknowledges the interaction between women’s values and uptake of virtual care. Relevant themes included the importance of convenience and cost savings to women through use of virtual care [34,36-39,41,44,45,47,48,51,53,59,60,62,63,73,74,76,79-82] (particularly for women with competing demands or preexisting challenges to health care access), the importance of clinician continuity when using virtual care, the high value placed on modality choice, and the potential loss of collateral benefits associated with in-person care.

#### Organization

The organization domain addresses aspects of a health care system that can hinder or promote the use of virtual care. Key findings for this domain included the importance of organization culture, which normalizes and supports virtual care; the responsibility to conduct outreach to women to raise awareness of virtual care offerings; and the need for adequate resource provision by health care organizations that support delivery of virtual care, including staffing, hardware, software, and established workflow processes.

#### Wider Context

This domain addresses systems outside organizations (eg, policies, politics, and sociocultural factors) in which virtual care occurs. Specifically, we found that virtual care was considered especially relevant in regions with a dearth of essential women’s health services or expertise [64,81,82] and when there were regional limitations in existing technology infrastructure (eg, bandwidth).

#### Embedding Over Time

The final domain addresses the need to continually work to incorporate virtual care delivery into care processes over time. We found limited information related to this domain in the identified literature. What we did find broadly noted the need for organizations to enact strategies to maintain and grow virtual care use.

### Findings Related to PHCRP

We found minimal data directly addressing PHCRP principles. Articles that provided PHCRP-relevant data had a primary objective to explore access or equity issues and largely focused on race or ethnicity; these studies did not include other social identities, such as religion or sexuality. The most common PHCRP construct addressed was structural determinism, such as distance to health care facilities, which was described as a structural barrier to access to care. Other PHCRP-relevant findings included unstable funding for women’s health clinical services as a contributor to disparities and the potential for phone-based care to address implicit bias by preventing clinicians from judging based on patient surroundings or physical state. We sought to analyze the positionality statements of included articles for evidence of authors’ reflection on the impact of their identity on their work; however, no identified articles included a positionality statement, although some demonstrated evidence of reflexivity.

## Discussion

### Principal Findings

In this qualitative evidence synthesis, we found that synchronous virtual care is highly valued by women for the increase in access afforded by its convenience and cost savings. These benefits are likely felt most strongly by women with preexisting barriers to care, such as limited transportation resources and overlapping, competing demands such as school, caregiving, and work commitments. We also found that virtual care is an important option for women in regions with a dearth of essential health services or expertise in women-specific care needs, such as contraception or abortion care. However, there are potential negative consequences related to the use of virtual care modalities, including detrimental effects on the patient-clinician relationship; a diminished sense of personal well-being for both patients and clinicians; and exacerbation of care barriers among populations considered disadvantaged and marginalized, particularly those with communication barriers. Our findings align with those seen in other studies of the impact of virtual care on health care experiences and add specific examples and nuance to how this impact is felt specifically among women and women’s health care clinicians [86-89].

Virtually delivered health care can be an acceptable strategy to address access issues to sex-specific health care needs. We found that women were particularly grateful for virtual care options for women-specific health care (eg, contraception and family planning services), which were not otherwise locally available. This was especially true for time-sensitive care needs, such as abortion and obstetric care. Our findings are consistent with previous data identifying telehealth as a viable strategy to supplement limited regional gynecologic resources [90]. Virtual care also has the potential to address disparities in access to care for women. Women’s health care deserts are often in rural areas [90,91] and are more likely to impact low-income individuals, those with lower education levels, and women of color [92-94]. Moreover, technology has been noted as a potential strategy to overcome insufficient communication between clinicians and Black women for key health issues, such as breast cancer risk [95].

Delivering high-quality virtual care requires the intentional consideration of determinants of health care, which apply to all patients but are more common or more impactful among women. For example, we found that while the convenience of virtual care was universally valued by women, it was particularly valuable for those who would otherwise need to miss work, identify viable transportation, and navigate complicated caregiving responsibilities to attend an appointment. Similarly, women and their clinicians broadly expressed concerns about safety and the ability to speak freely about health issues during virtual encounters, particularly when women lived with a partner who used violence. Privacy was often an issue due to children or others in the home and lack of a private physical location to participate in a health care encounter. While these barriers to care are applicable to all patients, women are more likely to forego needed care due to these competing demands than men. Health care systems and policy makers should consider how decisions about virtual modality availability may impact certain patient populations differently, particularly those with existing limitations on access to care (eg, difficulty with transportation and language barriers). In addition, health care systems can ensure that clinical processes and clinician trainings support practices that address and overcome barriers to optimal virtual care (eg, assessing patient privacy during a video visit and offering patient choice of visit modality).

Finally, the threat to the emotional connection between patients and clinicians may be a particularly important barrier to care for women, as evidence suggests that patient satisfaction with patient-clinician communication may differ between men and women [96]. Among women receiving obstetric and gynecologic care, for example, clinicians using a patient-centered communication style were preferred [97]. Themes related to the importance of modality choice and the value of continuity regardless of visit modality highlight the potential role of trauma-informed care practices and the need for training dedicated to communication strategies in the virtual setting [98]. Communication style and trust have been noted as particularly important for women regarding sensitive issues, such as disclosure of intimate partner violence [99] and sexual assault.

Considering our a priori approach to center on the voices of populations considered historically marginalized through our PHCRP-informed sampling and analysis strategies, we found relatively little literature focused on the needs of marginalized populations of women. Identified articles featured predominantly cis, heterosexual, and White participant populations in the existing literature from both the initial and updated searches. This lack of equitable representation limits the relevancy of our findings, and consequently, the field misses unique experiences of populations who are likely already experiencing barriers regarding access to care. Use of virtual care without intentional structure and action for inclusivity could perpetuate disparities in care and isolation of women who identify with these communities. Reasons for the lack of representation of marginalized voices in research are generally multifactorial and include a lack of cross-cultural competency, inadequate outreach about research participation opportunities, and inflexible protocols [100-102]. Researchers exploring the use of virtual modalities across health care settings should intentionally work to include individuals from populations considered marginalized to ensure virtual innovations promote equitable access to care, rather than amplifying existing disparities. For the purposes of our work, we plan to address this gap through individual qualitative interviews, prioritizing voices from populations poorly represented in the literature, and purposefully engaging with key shareholder groups and comparing those findings to those from this study.

### Limitations and Strengths

This review has certain limitations related to our approach and the identified studies. First, while we sought to include primary studies that examined virtual care for women in all clinical contexts, we primarily found studies relevant to women-specific issues (eg, abortion care and lactation services). Second, the eligible literature focused primarily on younger women of childbearing age, with limited exploration of the experiences of older women receiving virtual care. Given the potential generational differences in comfort with technology, we may have missed age-related disparities. Third, while we sought to optimize the representation of historically marginalized voices through oversampling articles from these populations, we found few studies that specifically centered on these populations. We also acknowledge the limitation of our research team and our own implicit biases during the review process, as the team is predominantly cisgender and White, with all members having postsecondary formal education. We aimed to mitigate this limitation by seeking input from multiple diverse patient advisory groups, technical advisory panels, and experts in women’s health care and gender-affirming care. Another missing perspective in this literature is that of administrative and support staff, whose experiences with virtual care could provide valuable insights into key logistical processes and factors that influence the success of virtual interactions. Our team is currently conducting a qualitative study that actively seeks to integrate these health care team perspectives. Fourth, we noted that eligible studies rarely reported underlying theory or frameworks to guide data collection or analysis, positionality statements, or a description of the researcher-participant relationship. Therefore, this limited our ability to evaluate the validity of analytic findings. Fifth, most studies were conducted around virtual care during the COVID-19 pandemic and few with details about the need to maintain and strengthen virtual care use over time. It is likely that certain challenges related to the initial implementation during the COVID-19 pandemic have resolved and new challenges have arisen. Future reviews should address the evolution of virtual care use after the COVID-19 pandemic. Finally, we also acknowledge the methodologic limitations of our approach. Importantly, as this work is a secondary analysis of qualitative data, we did not have access to raw primary data or coding but rather worked directly from the published findings, which may indicate a loss of thematic granularity. There is also the possible loss of an accurate representation of participant identity among study samples.

Despite these limitations, our work has many strengths. First, we used a systematic approach to identify eligible studies. Second, our process was guided by an a priori protocol based on established standards of rigor in qualitative evidence synthesis. Third, we used the NASSS framework, supplemented by the PHCRP, to guide data extraction, analysis, and interpretation, while purposefully selecting articles that included diverse populations. Fourth, our team consisted of individuals with diverse clinical and professional backgrounds, life experiences, and identities and actively sought input from diverse voices outside our research team. We identified key considerations for investigators conducting future qualitative research in this area (Table 1). Importantly, future research should establish a focus on how virtual care may impact populations of women considered underrepresented and marginalized differently to generate information to promote equitable access to virtual care. We recommend that all investigators consider health equity in their work, rather than deferring responsibility to those specifically focused on equity work. Finally, it is also important to explore the role of virtual care for women, not only in clinical contexts specific to women but also in broader contexts that apply to all patients (eg, primary care), where care may need to be tailored to meet the unique needs of women.

**Table 1:** Future research recommendations.

Category	Recommendation
Sample	Women from populations considered marginalized (eg, women with disabilities, immigrants, women considered sexually marginalized, gender-diverse individuals, women from communities considered racially and ethnically marginalized, and women from highly rural settings) Older women Clinicians for older women Women participating in group-based virtual care
Phenomenon of interest	Virtual care delivered to women in clinical contexts that serve all patients (eg, primary care) but which may require tailoring for women (eg, consideration of teratogenic medications in women of reproductive age) Comparison across modalities in different contexts (eg, phone, video, and in person) How to individualize the incorporation of virtual care to each woman’s needs
Evaluation	How experiences with virtual care change over time with practice, particularly since initial implementation during the COVID-19 pandemic
Research type	Clear reporting of the underlying theory
Other design features	Incorporation of PHCRP^a^ or other approaches to centering work on the experience of populations considered marginalized Inclusion of positionality statements and consideration of the relationship between the reviewer and participant.

^a^PHCRP: Public Health Critical Race Praxis.

### Conclusions

The use of virtual modalities affects the health care experience in ways that have unique implications for women, with the potential to either reduce or exacerbate existing disparities in access to care. Health care systems delivering virtual care should intentionally consider how to ensure care delivery is conducted in a manner that promotes equitable access and inclusive experiences for all sexes and genders.

## Appendix

Multimedia Appendix 1 Search terms.

Multimedia Appendix 2 Eligibility criteria.

Multimedia Appendix 3 Abstraction form.

Multimedia Appendix 4 Excluded studies.

Multimedia Appendix 5 Individual study characteristics.

Multimedia Appendix 6 Risk of bias assessments.

Multimedia Appendix 7 Confidence in the Evidence from Reviews of Qualitative Research assessments.

Multimedia Appendix 8 Summary of findings by Nonadoption, Abandonment, Scale-up, Spread, and Sustainability domains.

## Abbreviations

LGBTQ: lesbian, gay, bisexual, transgender, queer
LGBTQ+: lesbian, gay, bisexual, transgender, queer, and similar minority
NASSS: Nonadoption, Abandonment, Scale-up, Spread, and Sustainability
PHCRP: Public Health Critical Race Praxis

## References

[1] Zephyrin LC, Suennen L, Viswanathan P, Augenstein J, Bachrach D (2020). Transforming primary health care for women — part 1: a framework for addressing gaps and barriers. The Commonwealth Fund.

[2] Ng JH, Kaftarian SJ, Tilson WM, Gorrell P, Chen X, Chesley FD Jr, Scholle SH (2010). Self-reported delays in receipt of health care among women with diabetes and cardiovascular conditions. Womens Health Issues.

[3] Rapp KS, Volpe VV, Hale TL, Quartararo DF (2022). State-level sexism and gender disparities in health care access and quality in the United States. J Health Soc Behav.

[4] Owens G (2008). Gender differences in health care expenditures, resource utilization, and quality of care. J Manag Care Pharm.

[5] Ranji U, Salganicoff A, Rousseau D, Kaiser Family Foundation (2019). Barriers to care experienced by women in the United States. JAMA.

[6] Bean-Mayberry B, Moreau J, Hamilton AB, Yosef J, Joseph NT, Batuman F, Wight SC, Farmer MM (2022). Cardiovascular risk screening among women veterans: identifying provider and patient barriers and facilitators to develop a clinical toolkit. Womens Health Issues.

[7] Gatov E, Koziel N, Kurdyak P, Saunders NR, Chiu M, Lebenbaum M, Chen S, Vigod SN (2020). Epidemiology of interpersonal trauma among women and men psychiatric inpatients: a population-based study. Can J Psychiatry.

[8] McBain SA, Garneau-Fournier J, Turchik JA (2022). The relationship between provider gender preferences and perceptions of providers among veterans who experienced military sexual trauma. J Interpers Violence.

[9] Nagle-Yang S, Sachdeva J, Zhao LX, Shenai N, Shirvani N, Worley LL, Gopalan P, Albertini ES, Spada M, Mittal L, Moore Simas TA, Byatt N (2022). Trauma-informed care for obstetric and gynecologic settings. Matern Child Health J.

[10] Haider A, Bengs S, Luu J, Osto E, Siller-Matula JM, Muka T, Gebhard C (2020). Sex and gender in cardiovascular medicine: presentation and outcomes of acute coronary syndrome. Eur Heart J.

[11] Cader FA, Banerjee S, Gulati M (2022). Sex differences in acute coronary syndromes: a global perspective. J Cardiovasc Dev Dis.

[12] Radley DC, Shah A, Collins SR, Powe NR, Zephyrin LC (2024). Advancing racial equity in U.S. health care: the Commonwealth Fund 2024 state health disparities report. The Commonwealth Fund.

[13] Ferguson JM, Jacobs J, Yefimova M, Greene L, Heyworth L, Zulman DM (2021). Virtual care expansion in the Veterans Health Administration during the COVID-19 pandemic: clinical services and patient characteristics associated with utilization. J Am Med Inform Assoc.

[14] Ferguson JM, Goldstein KM, Zullig LL, Zulman DM (2024). Gender differences in adoption and frequency of virtual primary care among men and women veterans. J Womens Health (Larchmt).

[15] Reed ME, Huang J, Graetz I, Lee C, Muelly E, Kennedy C, Kim E (2020). Patient characteristics associated with choosing a telemedicine visit vs office visit with the same primary care clinicians. JAMA Netw Open.

[16] Singer A, Kosowan L, LaBine L, Shenoda D, Katz A, Abrams EM, Halas G, Wong ST, Talpade S, Kirby S, Baldwin A, Francois J (2022). Characterizing the use of virtual care in primary care settings during the COVID-19 pandemic: a retrospective cohort study. BMC Prim Care.

[17] Dickins KA, Malley A, Bartels SJ, Baggett TP, Looby SE (2021). Barriers, facilitators, and opportunities to optimize care engagement in a diverse sample of older low-income women: a qualitative study. Geriatr Nurs.

[18] (2020). Implementing telehealth in practice. Obstet Gynecol.

[19] (2022). 4 keys to delivering superior virtual care. American Hospital Association.

[20] Rheuban KS, Jin J (2025). Telehealth integration and optimization: improve patient care through virtual health care delivery. AMA STEPS Forward.

[21] Nayakarathna R, Neilson H, MacDougall D, Cowling T (2022). Virtual care use in primary care or specialty care settings. Can J Health Technol.

[22] Cantor AG, Nelson HD, Pappas M, Atchison C, Hatch B, Huguet N, Flynn B, McDonagh M (2023). Telehealth for women's preventive services for reproductive health and intimate partner violence: a comparative effectiveness review. J Gen Intern Med.

[23] Flemming K, Noyes J (2021). Qualitative evidence synthesis: where are we at?. Int J Qual Methods.

[24] Goldstein KM, Patel DB, Van Loon KA, Shapiro A, Rushton S, Lewinski AA, Lanford TJ, Cantrell S, Zullig LL, Wilson SM, Shepherd-Banigan M, Alton Dailey S, Sims C, Robinson C, Chawla N, Bosworth HB, Hamilton A, Naylor J, Gierisch JM (2023). Optimizing the equitable deployment of virtual care for women: protocol for a qualitative evidence synthesis examining patient and provider perspectives supplemented with primary qualitative data. Health Equity.

[25] Ford CL, Airhihenbuwa CO (2010). The public health critical race methodology: praxis for antiracism research. Soc Sci Med.

[26] Greenhalgh T, Wherton J, Papoutsi C, Lynch J, Hughes G, A'Court C, Hinder S, Fahy N, Procter R, Shaw S (2017). Beyond adoption: a new framework for theorizing and evaluating nonadoption, abandonment, and challenges to the scale-up, spread, and sustainability of health and care technologies. J Med Internet Res.

[27] Cooke A, Smith D, Booth A (2012). Beyond PICO: the SPIDER tool for qualitative evidence synthesis. Qual Health Res.

[28] Ames H, Glenton C, Lewin S (2019). Purposive sampling in a qualitative evidence synthesis: a worked example from a synthesis on parental perceptions of vaccination communication. BMC Med Res Methodol.

[29] Carroll C, Booth A, Leaviss J, Rick J (2013). "Best fit" framework synthesis: refining the method. BMC Med Res Methodol.

[30] Thomas J, Harden A (2008). Methods for the thematic synthesis of qualitative research in systematic reviews. BMC Med Res Methodol.

[31] Malterud K, Siersma VD, Guassora AD (2016). Sample size in qualitative interview studies: guided by information power. Qual Health Res.

[32] CASP checklists. Critical Appraisal Skills Programme.

[33] Lewin S, Booth A, Glenton C, Munthe-Kaas H, Rashidian A, Wainwright M, Bohren MA, Tunçalp Ö, Colvin CJ, Garside R, Carlsen B, Langlois EV, Noyes J (2018). Applying GRADE-CERQual to qualitative evidence synthesis findings: introduction to the series. Implement Sci.

[34] Srinivasulu S, Manze MG, Jones HE (2023). "I totally didn't need to be there in person": New York women's preferences for telehealth consultations for sexual and reproductive healthcare in primary care. Fam Pract.

[35] Pipkin A, Smith A, Shearn C (2022). Transition needs compassion: a thematic analysis of an online compassion-focused therapy group in a gender service. Mindfulness (N Y).

[36] Gorman JR, Drizin JH, Smith E, Corey S, Temple M, Rendle KA (2022). Feasibility of mindful after cancer: pilot study of a virtual mindfulness-based intervention for sexual health in cancer survivorship. J Sex Med.

[37] Parameswaran UD, Pentecost R, Williams M, Smid M, Latendresse G (2022). Experiences with use of technology and telehealth among women with perinatal depression. BMC Pregnancy Childbirth.

[38] Sessanna L, Nisbet P, Alanazi N, Lorissaint D, Auerbach SL, Chang YP, Lorenz RA (2021). The experience of participating in an 8-week mindfulness based stress reduction plus sleep retraining course among women living with multiple sclerosis. Clin Nurs Res.

[39] Tan G, Teo I, Srivastava D, Smith D, Smith SL, Williams W, Jensen MP (2013). Improving access to care for women veterans suffering from chronic pain and depression associated with trauma. Pain Med.

[40] Gomez-Roas MV, Davis KM, Leziak K, Jackson J, Williams BR, Feinglass JM, Grobman WA, Yee LM (2022). Postpartum during a pandemic: challenges of low-income individuals with healthcare interactions during COVID-19. PLoS One.

[41] Ireland S, Belton S, Doran F (2020). ‘I didn’t feel judged’: exploring women’s access to telemedicine abortion in rural Australia. J Prim Health Care.

[42] Lou S, Carstensen K, Vogel I, Hvidman L, Nielsen CP, Lanther M, Petersen OB (2019). Receiving a prenatal diagnosis of Down syndrome by phone: a qualitative study of the experiences of pregnant couples. BMJ Open.

[43] Silverio SA, De Backer K, Easter A, von Dadelszen P, Magee LA, Sandall J (2021). Women's experiences of maternity service reconfiguration during the COVID-19 pandemic: a qualitative investigation. Midwifery.

[44] Bogulski CA, Payakachat N, Rhoads SJ, Jones RD, McCoy HC, Dawson LC, Eswaran H (2023). A comparison of audio-only and audio-visual tele-lactation consultation services: a mixed methods approach. J Hum Lact.

[45] Boydell N, Reynolds-Wright JJ, Cameron ST, Harden J (2021). Women's experiences of a telemedicine abortion service (up to 12 weeks) implemented during the coronavirus (COVID-19) pandemic: a qualitative evaluation. BJOG.

[46] Ericson J, Flacking R, Udo C (2017). Mothers' experiences of a telephone based breastfeeding support intervention after discharge from neonatal intensive care units: a mixed-method study. Int Breastfeed J.

[47] Kerestes C, Delafield R, Elia J, Chong E, Kaneshiro B, Soon R (2021). "It was close enough, but it wasn't close enough": a qualitative exploration of the impact of direct-to-patient telemedicine abortion on access to abortion care. Contraception.

[48] Fix L, Seymour JW, Sandhu MV, Melville C, Mazza D, Thompson TA (2020). At-home telemedicine for medical abortion in Australia: a qualitative study of patient experiences and recommendations. BMJ Sex Reprod Health.

[49] Howell K, Alvarado G, Waymouth M, Demirci J, Rogers R, Ray K, Uscher-Pines L (2023). Acceptability of telelactation services for breastfeeding support among Black parents: semistructured interview study. J Med Internet Res.

[50] Goldstein KM, Zullig LL, Andrews SM, Sperber N, Lewinski AA, Voils CI, Oddone EZ, Bosworth HB (2021). Patient experiences with a phone-based cardiovascular risk reduction intervention: are there differences between women and men?. Patient Educ Couns.

[51] Cox A, Faithfull S (2015). Aiding a reassertion of self: a qualitative study of the views and experiences of women with ovarian cancer receiving long-term nurse-led telephone follow-up. Support Care Cancer.

[52] Zilliacus EM, Meiser B, Lobb EA, Kirk J, Warwick L, Tucker K (2010). Women's experience of telehealth cancer genetic counseling. J Genet Couns.

[53] Christiansen MG, Pappot H, Pedersen C, Jarden M, Mirza MR, Piil K (2022). Patient perspectives and experiences of the rapid implementation of digital consultations during COVID-19 - a qualitative study among women with gynecological cancer. Support Care Cancer.

[54] Vincze L, Rollo ME, Hutchesson MJ, Callister R, Thompson DI, Collins CE (2018). Postpartum women's perspectives of engaging with a dietitian and exercise physiologist via video consultations for weight management: a qualitative evaluation. Healthcare (Basel).

[55] Nguyen-Grozavu F, Ko E, Valadez Galindo A (2023). Gauging the changing landscape: telehealth perceptions among hispanic females with breast cancer. Int J Environ Res Public Health.

[56] Davenport A, Brunn E, Creswell M, Sholklapper T, Ringel N, Gutman R (2022). Exploring patient perspectives surrounding telemedicine versus in-person preoperative visits. Urogynecology.

[57] Buse CR, Kelly EA, Muss HB, Nyrop KA (2022). Perspectives of older women with early breast cancer on telemedicine during post-primary treatment. Support Care Cancer.

[58] Ehrenreich K, Kaller S, Raifman S, Grossman D (2019). Women's experiences using telemedicine to attend abortion information visits in Utah: a qualitative study. Womens Health Issues.

[59] Huang I, Delay R, Boulware A, McHugh A, Wong ZJ, Whitaker AK, Stulberg D, Hasselbacher L (2022). Telehealth for contraceptive care: lessons from staff and clinicians for improving implementation and sustainability in Illinois. Contracept X.

[60] Song B, Boulware A, Wong ZJ, Huang I, Whitaker AK, Hasselbacher L, Stulberg D (2022). "This has definitely opened the doors": provider perceptions of patient experiences with telemedicine for contraception in Illinois. Perspect Sex Reprod Health.

[61] Beatty K, Smith MG, Khoury AJ, Ventura LM, Ariyo O, de Jong J, Surles K, Slawson D (2023). Contraceptive care service provision via telehealth early in the COVID-19 pandemic at rural and urban federally qualified health centers in 2 southeastern states. J Rural Health.

[62] Beatty KE, Smith MG, Khoury AJ, Ventura LM, Ariyo T, de Jong J, Surles K, Rahman A, Slawson D (2022). Telehealth for contraceptive care during the initial months of the COVID-19 pandemic at local health departments in 2 US states: a mixed-methods approach. J Public Health Manag Pract.

[63] Moreau JL, Cordasco KM, Young AS, Oishi SM, Rose DE, Canelo I, Yano EM, Haskell SG, Hamilton AB (2018). The use of telemental health to meet the mental health needs of women using department of veterans affairs services. Womens Health Issues.

[64] Ghidei W, Montesanti S, Wells L, Silverstone PH (2022). Perspectives on delivering safe and equitable trauma-focused intimate partner violence interventions via virtual means: a qualitative study during COVID-19 pandemic. BMC Public Health.

[65] Montesanti S, Ghidei W, Silverstone P, Wells L, Squires S, Bailey A (2022). Examining organization and provider challenges with the adoption of virtual domestic violence and sexual assault interventions in Alberta, Canada, during the COVID-19 pandemic. J Health Serv Res Policy.

[66] Henry A, Yang J, Grattan S, Roberts L, Lainchbury A, Shanthosh J, Cullen P, Everitt L (2022). Effects of the COVID-19 pandemic and telehealth on antenatal screening and services, including for mental health and domestic violence: an Australian mixed-methods study. Front Glob Womens Health.

[67] Sterba KR, Johnson EE, Douglas E, Aujla R, Boyars L, Kruis R, Verdin R, Grater R, King K, Ford D, Guille C (2023). Implementation of a women's reproductive behavioral health telemedicine program: a qualitative study of barriers and facilitators in obstetric and pediatric clinics. BMC Pregnancy Childbirth.

[68] Simon N, Cunningham E, Samuel V, Waters C (2024). Videoconference-delivered group acceptance commitment therapy for perinatal mood and anxiety disorders: facilitators views and recommendations. J Reprod Infant Psychol.

[69] Spiby H, Walsh D, Green J, Crompton A, Bugg G (2014). Midwives' beliefs and concerns about telephone conversations with women in early labour. Midwifery.

[70] Reynolds-Wright JJ, Boydell N, Cameron S, Harden J (2022). A qualitative study of abortion care providers' perspectives on telemedicine medical abortion provision in the context of COVID-19. BMJ Sex Reprod Health.

[71] Madden N, Emeruwa UN, Friedman AM, Aubey JJ, Aziz A, Baptiste CD, Coletta JM, D'Alton ME, Fuchs KM, Goffman D, Gyamfi-Bannerman C, Kondragunta S, Krenitsky N, Miller RS, Nhan-Chang CL, Saint Jean AM, Shukla HP, Simpson LL, Spiegel ES, Yates HS, Zork N, Ona S (2020). Telehealth uptake into prenatal care and provider attitudes during the COVID-19 pandemic in New York City: a quantitative and qualitative analysis. Am J Perinatol.

[72] Kozica-Olenski SL, Garth B, Boyle JA, Vincent AJ (2023). Menopause care delivery in the time of COVID-19: evaluating the acceptability of telehealth services for women with early and usual age menopause. Climacteric.

[73] Hemming P, Kaur R, Meiser B, McKinley J, Young MA, James PA, Forrest LE (2021). Oncologists' perspectives of telephone genetic counseling to facilitate germline BRCA1/2 testing for their patients with high-grade serous ovarian cancer. J Community Genet.

[74] Corcoran J, Marley Campbell C, Ladores S (2023). Transitioning to telehealth during the coronavirus disease 2019 pandemic: perspectives from partners of women with cystic fibrosis and healthcare providers. Chronic Illn.

[75] Allison MK, Curran GM, Walsh WA, Dworkin ER, Zielinski MJ (2023). Factors affecting telemedicine implementation in emergency departments and nurses' perceptions of virtual sexual assault nurse examiner consultation for sexual assault survivors. J Forensic Nurs.

[76] Singla DR, Hossain S, Ravitz P, Schiller CE, Andrejek N, Kim J, La Porte L, Meltzer-Brody SE, Silver R, Vigod SN, Jung JW, Dimidjian S (2022). Adapting behavioral activation for perinatal depression and anxiety in response to the COVID-19 pandemic and racial injustice. J Affect Disord.

[77] Hensel JM, Yang R, Vigod SN, Desveaux L (2020). Videoconferencing at home for psychotherapy in the postpartum period: identifying drivers of successful engagement and important therapeutic conditions for meaningful use. Couns Psychother Res.

[78] Howard A, Wang S, Adachi J, Yadama A, Bhat A (2023). Facilitators of and barriers to perinatal telepsychiatry care: a qualitative study. BMJ Open.

[79] Grindlay K, Grossman D (2016). Telemedicine provision of medical abortion in Alaska: through the provider’s lens. J Telemed Telecare.

[80] Beaver K, Williamson S, Chalmers K (2010). Telephone follow-up after treatment for breast cancer: views and experiences of patients and specialist breast care nurses. J Clin Nurs.

[81] Demirci J, Kotzias V, Bogen DL, Ray KN, Uscher-Pines L (2019). Telelactation via mobile app: perspectives of rural mothers, their care providers, and lactation consultants. Telemed J E Health.

[82] Grindlay K, Lane K, Grossman D (2013). Women's and providers' experiences with medical abortion provided through telemedicine: a qualitative study. Womens Health Issues.

[83] Williamson S, Beaver K, Gardner A, Martin-Hirsch P (2018). Telephone follow-up after treatment for endometrial cancer: a qualitative study of patients' and clinical nurse specialists' experiences in the ENDCAT trial. Eur J Oncol Nurs.

[84] Kissler K, Thumm EB, Smith DC, Anderson JL, Wood RE, Johnson R, Roberts M, Carmitchel-Fifer A, Patterson N, Amura CR, Barton AJ, Jones J (2024). Perinatal telehealth: meeting patients where they are. J Midwifery Womens Health.

[85] Ehrenreich K, Marston C (2019). Spatial dimensions of telemedicine and abortion access: a qualitative study of women's experiences. Reprod Health.

[86] Wu K, Dang Nguyen M, Rouleau G, Azavedo R, Srinivasan D, Desveaux L (2023). Understanding how virtual care has shifted primary care interactions and patient experience: a qualitative analysis. J Telemed Telecare.

[87] Hedden L, Spencer S, Mathews M, Gard Marshall E, Lukewich J, Asghari S, Gill P, McCracken RK, Vaughan C, Wong E, Buote R, Meredith L, Moritz L, Ryan D, Schacter G (2024). "Technology has allowed us to do a lot more but it's not necessarily the panacea for everybody": family physician perspectives on virtual care during the COVID-19 pandemic and beyond. PLoS One.

[88] Alhajri N, Simsekler MC, Alfalasi B, Alhashmi M, AlGhatrif M, Balalaa N, Al Ali M, Almaashari R, Al Memari S, Al Hosani F, Al Zaabi Y, Almazroui S, Alhashemi H, Baltatu OC (2021). Physicians' attitudes toward telemedicine consultations during the COVID-19 pandemic: cross-sectional study. JMIR Med Inform.

[89] Rodriguez JA, Betancourt JR, Sequist TD, Ganguli I (2021). Differences in the use of telephone and video telemedicine visits during the COVID-19 pandemic. Am J Manag Care.

[90] Friedman S, Shaw JG, Hamilton AB, Vinekar K, Washington DL, Mattocks K, Yano EM, Phibbs CS, Johnson AM, Saechao F, Berg E, Frayne SM (2022). Gynecologist supply deserts across the VA and in the community. J Gen Intern Med.

[91] Taporco JS, Wolfe E, Chavez G, Allen Z, Estrada J, Thomson K, Mettling J, Kennedy M (2021). Kansas maternity deserts: a cross-sectional study of rural obstetric providers. Rural Remote Health.

[92] Lee J, Manalew WS (2023). Reasons for not pursuing virtual prenatal care in 2020 through 2021 and policy implications. Telemed J E Health.

[93] Mistry SK, Shaw M, Raffan F, Johnson G, Perren K, Shoko S, Harris-Roxas B, Haigh F (2022). Inequity in access and delivery of virtual care interventions: a scoping review. Int J Environ Res Public Health.

[94] Kreitzer RJ, Smith CW, Kane KA, Saunders TM (2021). Affordable but inaccessible? Contraception deserts in the US states. J Health Polit Policy Law.

[95] Anderson JN, Graff JC, Krukowski RA, Schwartzberg L, Vidal GA, Waters TM, Paladino AJ, Jones TN, Blue R, Kocak M, Graetz I (2021). "Nobody will tell you. You've got to ask!": an examination of patient-provider communication needs and preferences among Black and White women with early-stage breast cancer. Health Commun.

[96] Schmid Mast M, Hall JA, Roter DL (2007). Disentangling physician sex and physician communication style: their effects on patient satisfaction in a virtual medical visit. Patient Educ Couns.

[97] Janssen SM, Lagro-Janssen AL (2012). Physician's gender, communication style, patient preferences and patient satisfaction in gynecology and obstetrics: a systematic review. Patient Educ Couns.

[98] Gerber MR, Elisseou S, Sager ZS, Keith JA (2020). Trauma-informed telehealth in the COVID-19 era and beyond. Fed Pract.

[99] Battaglia TA, Finley E, Liebschutz JM (2003). Survivors of intimate partner violence speak out: trust in the patient-provider relationship. J Gen Intern Med.

[100] Hasson Charles RM, Sosa E, Patel M, Erhunmwunsee L (2022). Health disparities in recruitment and enrollment in research. Thorac Surg Clin.

[101] Louis-Jacques AF, Heuberger AJ, Mestre CT, Evans VF, Wilson RE, Gurka MJ, Lewis TR (2023). Improving racial and ethnic equity in clinical trials enrolling pregnant and lactating individuals. J Clin Pharmacol.

[102] Oyer RA, Hurley P, Boehmer L, Bruinooge SS, Levit K, Barrett N, Benson A, Bernick LA, Byatt L, Charlot M, Crews J, DeLeon K, Fashoyin-Aje L, Garrett-Mayer E, Gralow JR, Green S, Guerra CE, Hamroun L, Hardy CM, Hempstead B, Jeames S, Mann M, Matin K, McCaskill-Stevens W, Merrill J, Nowakowski GS, Patel MI, Pressman A, Ramirez AG, Segura J, Segarra-Vasquez B, Hanley Williams J, Williams JE, Winkfield KM, Yang ES, Zwicker V, Pierce LJ (2022). Increasing racial and ethnic diversity in cancer clinical trials: an American Society of Clinical Oncology and Association of Community Cancer Centers joint research statement. J Clin Oncol.

