# Supplementary material for: Experiences Receiving and Delivering Virtual Health Care For Women: Qualitative Evidence Synthesis
Source: J Med Internet Res. 2025 May 15;27:e68314. doi: 10.2196/68314 (PMC12123244; doi:10.2196/68314)
Supplement: Multimedia Appendix 1 [file jmir_v27i1e68314_app1.docx]

**Database: MEDLINE (via Ovid), Embase (via Elsevier), and CINAHL Complete (via EBSCO)**

| **Description** | **Search set** | **Search strategy** |
| --- | --- | --- |
| *Virtual Care terms* | #1 | exp Telemedicine/ or exp Remote Consultation/ or Videoconferencing/ or Telephone/ or Cell phone/ or Smartphone/ |
|  | #2 | (telehealth or tele-health or telemedicine or tele-medicine or telemedical or tele-medical or telecare or tele-care or teleprimary care or tele-primary care or tele-PCP or tele-visit or televisit or tele-visits or televisits or teleconference or tele-conference or teleconferences or tele-conferences or telemanage or tele-manage or telemanagement or tele-management or telepharmacy or tele-pharmacy or telepharmacies or tele-pharmacies or telepharmacist or telepharmacists or tele-pharmacist or tele-pharmacists or telegynecology or tele-gynecology or telegynecologist or tele-gynecologist or telegynecologists or tele-gynecologists or teleobstetrics or tele-obstetrics or teleobstetrician or tele-obstetrician or teleobstetricians or tele-obstetricianstelenurse or tele-nurse or tele-nurses or tele-nursing or telenurse or telenurses or telenursing or telepsychiatrist or tele-psychiatrist or telepsychiatrists or tele-psychiatrists or telepsychiatry or tele-psychiatry or telecounselling or tele-counselling or telecounselor or tele-counselor or teleconselors or tele-counselors or teleintervention or tele-interventions or teleinterventions or tele-interventions or tele-therapy or tele-therapies or teletherapy or teletherapies).ti,ab. |
|  | #3 | ((virtual or virtually or video or video-based or videobased or videoconference or video-conference or videoconferences or video-conferences or videoconferencing or video-conferencing or webconference or web-conference or webconferences or web-conferences or webconferencing or web-conferencing or Zoom or Skype or WebEx or FaceTime or GoToMeeting or "web based" or web-based or webbased or online or telephone or tele-phone or telephones or tele-phones or telephoned or tele-phoned or cellphone or cellphones or cell-phone or cell-phones or "cell phone" or "cell phones" or smartphone or smartphones or smart-phone or smart-phones or "smart phone" or "smart phones" or "cellular phone" or "cellular phones" or "mobile device" or "mobile devices" or "mobile phone" or "mobile phones" or iPhone or iPhones or iPad or iPads or Android) adj5 (care or cared or health or healthcare or appointment or appointments or meeting or meetings or met or meet or visit or visits or clinic or clinics or medicine or medical or therapy or therapies or therapeutic or therapeutics or intervention or interventions or treatment or treatments or treat or treats or treated or manage or manages or management or managed or physician or physicians or clinician or clinicians or doctor or doctors or patient or patients or nurse or nurses or nursing or diagnose or diagnosis or diagnoses or diagnostic or prescribe or prescribes or prescribing or prescription or prescriptions or pharmacy or pharmacies or pharmacist or pharmacists or counsel or counsels or counselled or counselling or counsellor or counsellors or psychiatry or psychiatrist or psychiatrists or "mental health" or gynecology or gynecologist or gynecologists or obstetrics or obstetrician or obstetricians or "OB/GYN" or OBGYN or "OB GYN")).ti,ab. |
|  | #4 | (Tele adj2 (care or cared or health or healthcare or appointment or appointments or meeting or meetings or visit or visits or clinic or clinics or medicine or medical or session or sessions or therapy or therapies or therapeutic or therapeutics or intervention or interventions or treatment or treatments or treat or treats or treated or manage or manages or management or managed or physician or physicians or clinician or clinicians or doctor or doctors or nurse or nurses or nursing or diagnose or diagnosis or diagnoses or diagnostic or prescribe or prescribes or prescribed or prescribing or prescription or prescriptions or pharmacy or pharmacies or pharmacist or pharmacists or psychiatry or psychiatrists or psychiatrist or "mental health" or counselling or counsel or counsels or counselled or counsellor or counsellors or gynecology or gynecologist or gynecologists or obstetrics or obstetrician or obstetricians or "OB/GYN" or OBGYN or "OB GYN")).ti,ab. |
|  | #5 | ((remote or remotely) adj3 (care or cared or health or healthcare or appointment or appointments or meeting or meetings or visit or visits or therapy or therapies or therapeutic or therapeutics or intervention or interventions or treatment or treatments or treat or treats or treated or management or managed or diagnose or diagnosis or diagnoses or diagnostic or prescribe or prescribes or prescribed or prescribing or prescription or prescriptions or "mental health" or counselling or counsels or counselled or counsel or gynecology or gynecologist or gynecologists or obstetrics or obstetrician or obstetricians or "OB/GYN" or OBGYN or "OB GYN")).ti,ab. |
| *Combining* | #6 | 1 or 2 or 3 or 4 or 5 |
| *Women or women's health terms* | #7 | exp Women/ or exp Women's Health/ or exp Women's Health Services/ or exp Health Services for Transgender Persons/ or exp Homosexuality, Female/ or (woman or women or womens or womans or "women s" or "woman s" or Female or females or "female s" or transwoman or trans-woman or transwomans or "transwoman s" or trans-womans or "trans-woman s" or trans-women or transwomen or transwomens or "transwomen s" or trans-womens or "transwomen s" or non-binary or nonbinary or transman or trans-man or transmans or "transman s" or trans-mans or "trans-man s" or transmen or trans-men or transmens or trans-mens or "transmen s" or "transmen s" or trans or transgender or transgendered or lesbian or lesbians).ti,ab. |
|  | #8 | exp Breast Diseases/ or exp Breast Neoplasms/ or ("breast health" or "breast disease" or "breast diseases" or "breast cancer" or "breast cancers" or "breast neoplasm" or "breast neoplasms").ti,ab. |
|  | #9 | exp Pregnant Women/ or exp Maternal Health/ or exp Maternal Health Services/ or exp Prenatal Care/ or exp Perinatal Care/ or exp Postnatal Care/ or exp Postpartum Period/ or exp Depression, Postpartum/ or exp Pregnancy/ or exp Pregnancy Complications/ or exp Breast Feeding/ or exp Lactation/ or (pregnancy or pregnancies or pregnant or pregnancy-induced or pregnancy-associated or prenatal or pre-natal or prenatally or pre-natally or perinatal or peri-natal or perinatally or peri-natally or postnatal or post-natal or postnatally or post-natally or maternal or maternally or postpartum or post-partum or breastfeeding or "breast feeding" or lactation).ti,ab. |
|  | #10 | exp Contraception/ or exp Hormonal Contraception/ or exp Contraceptives, Oral/ or exp "Contraceptive Devices, Female"/ or Reproductive Health Services/ or exp Preconception Care/ or exp Family Planning Services/ or exp Fertility/ or exp Infertility/ or exp Fertility Clinics/ or exp Abortion, Induced/ or exp Abortion, Spontaneous/ or exp Levonorgestrel/ or (conception or preconception or pre-conception or "reproductive health" or "reproductive care" or "reproductive healthcare" or "reproductive plan" or "reproductive planning" or "family planning" or fertility or infertility or contraception or contraceptive or contraceptives or "morning after pill" or "morning after pills" or levonorgestrel or "plan b" or abortifacient or abortifacients or misoprostol or mifepristone or RU-486 or abortion or abortions).ti,ab. |
|  | #11 | exp Menopause/ or (menopause or menopausal or perimenopause or peri-menopause or perimenopausal or peri-menopausal or premenopause or pre-menopause or premenopausal or pre-menopausal or postmenopause or post-menopause or postmenopausal or post-menopausal or climacteric or "hot flash" or "hot flashes").ti,ab. |
|  | #12 | exp Menstrual Cycle/ or exp Menstruation Disturbances/ or (menstruation or menstruate or menstruates or menstruating or menstruated or menses or menstrual or dysmenorrhea or "painful period" or "painful periods" or "irregular period" or "irregular periods" or amenorrhea or menorrhagia or oligomenorrhea or premenstrual or pre-menstrual).ti,ab. |
|  | #13 | exp Urinary Tract Infections/ or exp Pelvic Floor Disorders/ or exp Polycystic Ovary Syndrome/ or exp Genital Neoplasms, Female/ or (PCOS or "polycystic ovary syndrome" or UTI or "urinary tract infection" or "urinary tract infections" or "pelvic floor disorder" or "pelvic floor disorders" or "disorders of the pelvic floor" or "pelvic floor health" or "pelvic floor prolapse").ti,ab. or ((ovary or ovaries or ovarian or uterine or uterus or cervical or cervix or vaginal or vagina or vaginas or fallopian or fallopians or endometrial or endometrium or endometriod or vulva or vulvas or vulvar) adj3 (cancer or cancers or cancerous or adenocarcinoma or adenocarcinomas or tumor or tumour or tumors or tumours or malignancy or malignancies or malignant or metastasis or metastases or metastasize or metastasizes or metastatic or neoplasm or neoplasms or cyst or cysts)).ti,ab. |
|  | #14 | exp Domestic Violence/ or exp Intimate Partner Violence/ or exp Spousal Abuse/or exp Battered Women/ or exp Rape/ or ((sex or sexual or sexually or domestic or partner or spouse or spousal or physical or physically) adj3 (abuse or abuses or abused or abuser or abusers or abusive or violence or violent or assault or assaults or assaulted)).ti,ab. or (rape or rapes or raped).ti,ab. |
| *Combining* | #15 | 7 or 8 or 9 or 10 or 11 or 12 or 13 or 14 |
| *Qualitative study filter* | #16 | exp Qualitative Research/ or exp Focus Groups/ or exp Interviews as Topic/ or (qualitative or qualitatively or "focus group" or "focus groups" or "group discussion" or "group discussions" or ethnograph or ethnographic or ethnography or ethnographies or autoethnography or autoethnographies or autoethnographic or "key informant" or "lived experience" or "lived experiences" or phenomenology or phenomenological or "mixed method" or "mixed methods" or mixed-methods or mixed-method).ti,ab. or ((semi-structured or semistructured or in-depth or indepth) adj5 (interview or interviews or interviewed or interviewing or discussion or discussions)).ti,ab. or ((stakeholder or stakeholders) adj2 (interview or interviews or interviewed or interviewing or discussion or discussions)).ti,ab. or (thematic adj2 (analysis or analyses)).ti,ab. |
| *Combining* | #17 | 6 and 15 and 16 |
| *Exclusions – study designs* | #18 | 17 not (case reports OR editorial OR letter OR comment OR congress).pt. |
| *Exclusions– animal-only research* | #19 | 18 not (exp animals/ not exp humans/) |
| *Date Limit* | #20 | Limit 19 to da=20100101-20230101 |
| *Language Limit* | #21 | 20 and English.lg. |
