# Supplementary material for: Experiences Receiving and Delivering Virtual Health Care For Women: Qualitative Evidence Synthesis
Source: J Med Internet Res. 2025 May 15;27:e68314. doi: 10.2196/68314 (PMC12123244; doi:10.2196/68314)
Supplement: Multimedia Appendix 2 [file jmir_v27i1e68314_app2.docx]

| **Inclusion criteria** | **Exclusion criteria** |
| --- | --- |
| **Sample** | |
| - Individuals identifying as women or female or gender diverse who have been offered or received synchronous clinical care via a virtual modality for any condition (e.g., gender-specific or gender-neutral) - Clinical team members involved in providing virtual care to individuals identifying as women or female - *Virtual modalities* include clinical care provision delivered synchronously (i.e., in real time) between a patient(s) and clinical team member specifically via video-based conferencing or telephone - *Clinical care provision* to include 1:1 or group-based encounters delivered as part of a clinic setting and which is provided by or consulted to by a treating clinician - *Clinical team members* include any professional role involved in delivering clinic-based health care or coordinating/scheduling of clinic-based care, including administrative staff (e.g., management, clinic-based administrative staff), nursing, prescribing-providers - Focus on parent as patient (e.g., lactation consultation) | - Individuals identifying as cisgender men or male. Assigned male at birth but do not identify as transgender or gender diverse - Mixed gender populations for which the results are not stratified, or intervention is not sub-grouped by gender - Virtual care modalities for asynchronous (i.e., not in real time) including secure messaging, static health related webpages, store, and forward services - Text-based chat – even if synchronous - Clinician to clinician virtual communication that does not include patient participation (e.g., e-consults) - Non-specific peer led support groups - Sample exclusively under 18, or mixed age range with a mean or standard deviation under 18 - Focus on infant or child rather than parent as patient |
| **Phenomenon of interest** | |
| - Virtual care delivered remotely (i.e., patient and provider in separate physical locations) and which involves the transmission of clinical information from patient to clinical team synchronously. Bidirectional information exchange between provider and clinician (e.g., not a health education workshop led by a clinician) - Care provided by the VA already (e.g., lactation consultation) - Clinician experiences delivering care to women digitally - Studies that include novel interventions, pilots, and usual care that focuses on the actual experiences of women using telehealth, rather than perceived/anticipated experiences. We will include articles that assess the feasibility of a virtual modality for usual care as well as articles about women who sought/want telehealth but were unable to receive telehealth (e.g., due to poor fit, lack of access, etc.) - Studies about IPV, DV, or SA that specifically focus on these topics as experienced by women or gender diverse people | - Self-management interventions that do not involve communication with a clinical team member or do not involve a patient sharing some of their own information - Virtual health care that is asynchronous as described above - Non-specific peer support groups - Support delivered unrelated to specific clinical conditions (e.g., benefits process support, training on financial literacy) - Mobile App interventions or any interventions where the virtual care part is not required or the dominant part - Care not provided by the VA (e.g., neonatal care) - Articles about clinicians that do not pertain to delivery of care to women digitally (e.g., wellbeing of clinicians) - Articles about the feasibility of novel interventions or potential experiences of a novel intervention that happens to have a virtual modality - Studies about IPV, DV, or SA that **do not** specifically focus on these topics as experienced by women or gender diverse people |
| **Design** | |
| - Synchronous data collection gathered via interviews (individual, dyad, group; semi-structured or structured) - Focus groups; observations; ethnographies | - Not open-ended surveys or asynchronous means of collecting data - Content analysis - Protocol papers - Systematic reviews and scoping reviews (tag for later review if relevant, however) |
| **Evaluation** | |
| Primary purpose of data collection is to evaluate the experiences and perspectives around being offered or receiving virtual care for women or the delivery of synchronous virtual care to individuals who identify as women or female | |
| **Research type** | |
| Qualitative or mixed or multiple methods | Quantitative-only study designs |
| **Countries** | |
| [Organization for Economic Co-operation and Development (OECD) Member Countries](https://www.oecd.org/about/document/ratification-oecd-convention.htm) | Non-OECD |
| **Time** | |
| Publications starting January 1^st^, 2010 | Publications from December 31, 2009, or earlier |
| **Publications** | |
| Full, peer-reviewed manuscripts | Posters, abstracts, letters |

DV: domestic violence, IPV: interpersonal violence, OECD: organization for economic co-operation and development, SA: sexual assault
