# Supplementary figures and images for: Experiences Receiving and Delivering Virtual Health Care For Women: Qualitative Evidence Synthesis
Source: J Med Internet Res. 2025 May 15;27:e68314. doi: 10.2196/68314 (PMC12123244; doi:10.2196/68314)

**
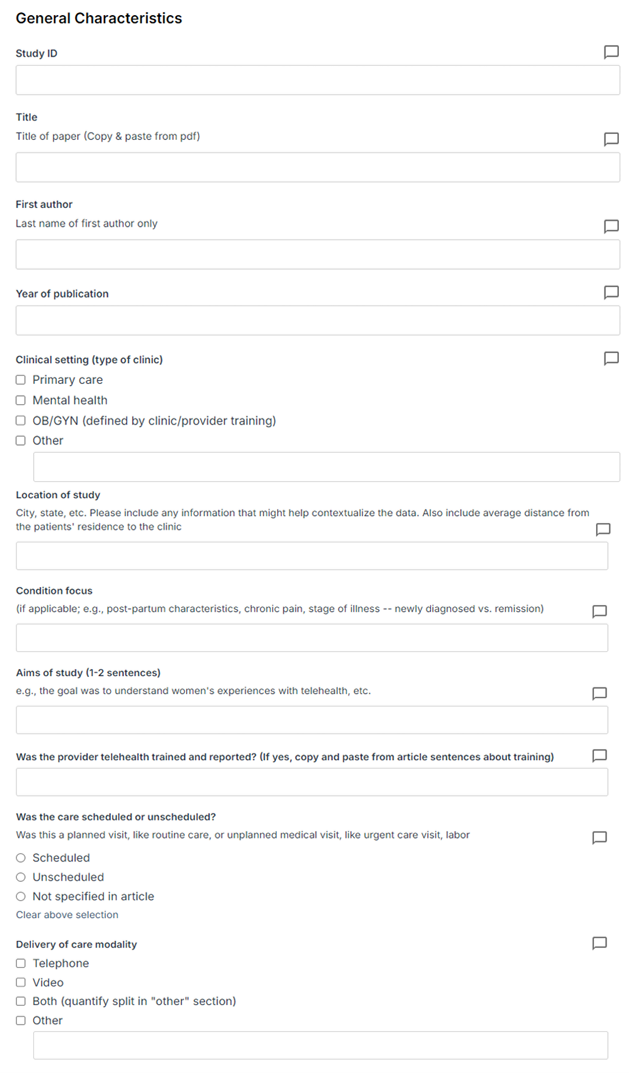
**


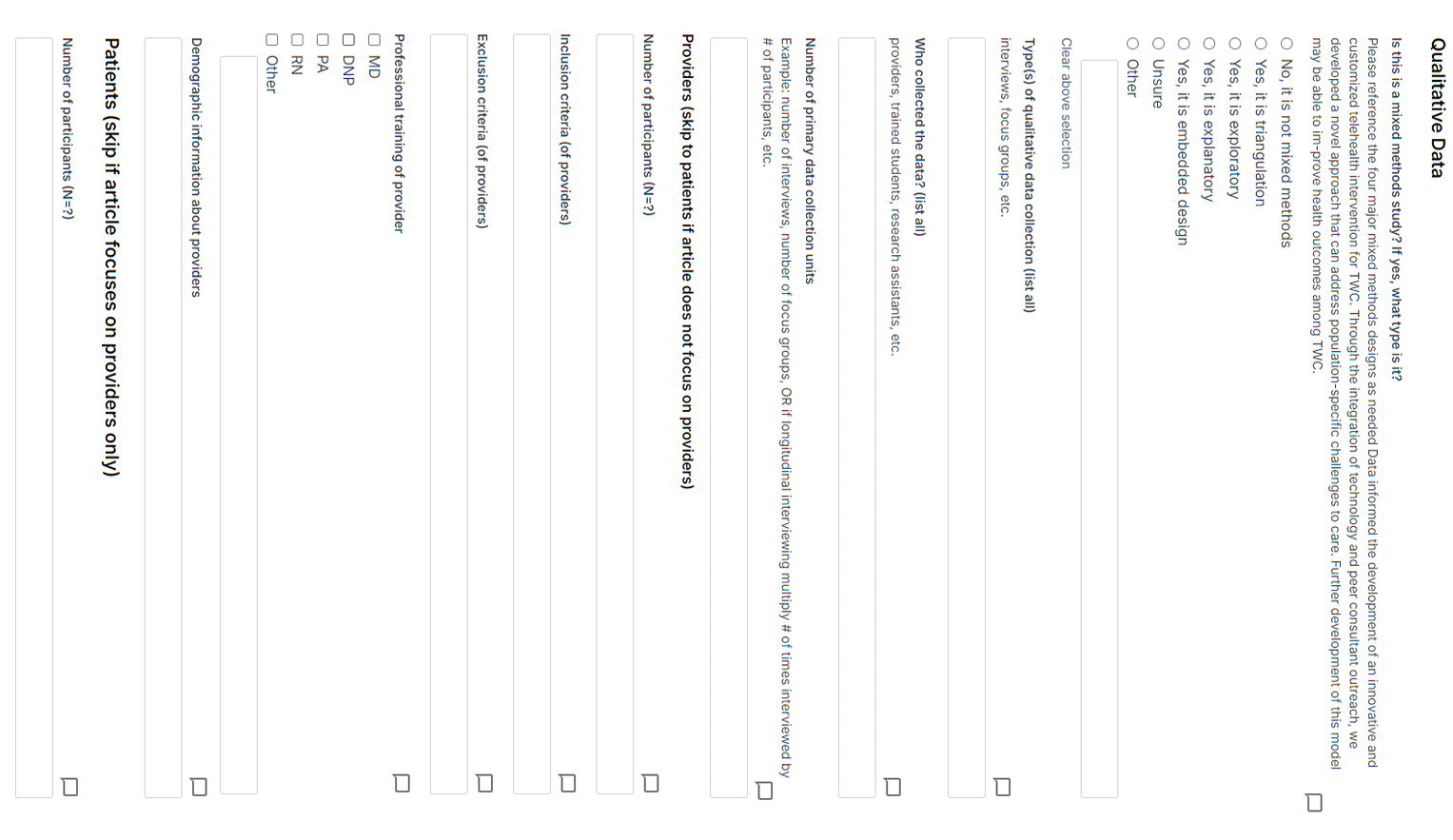

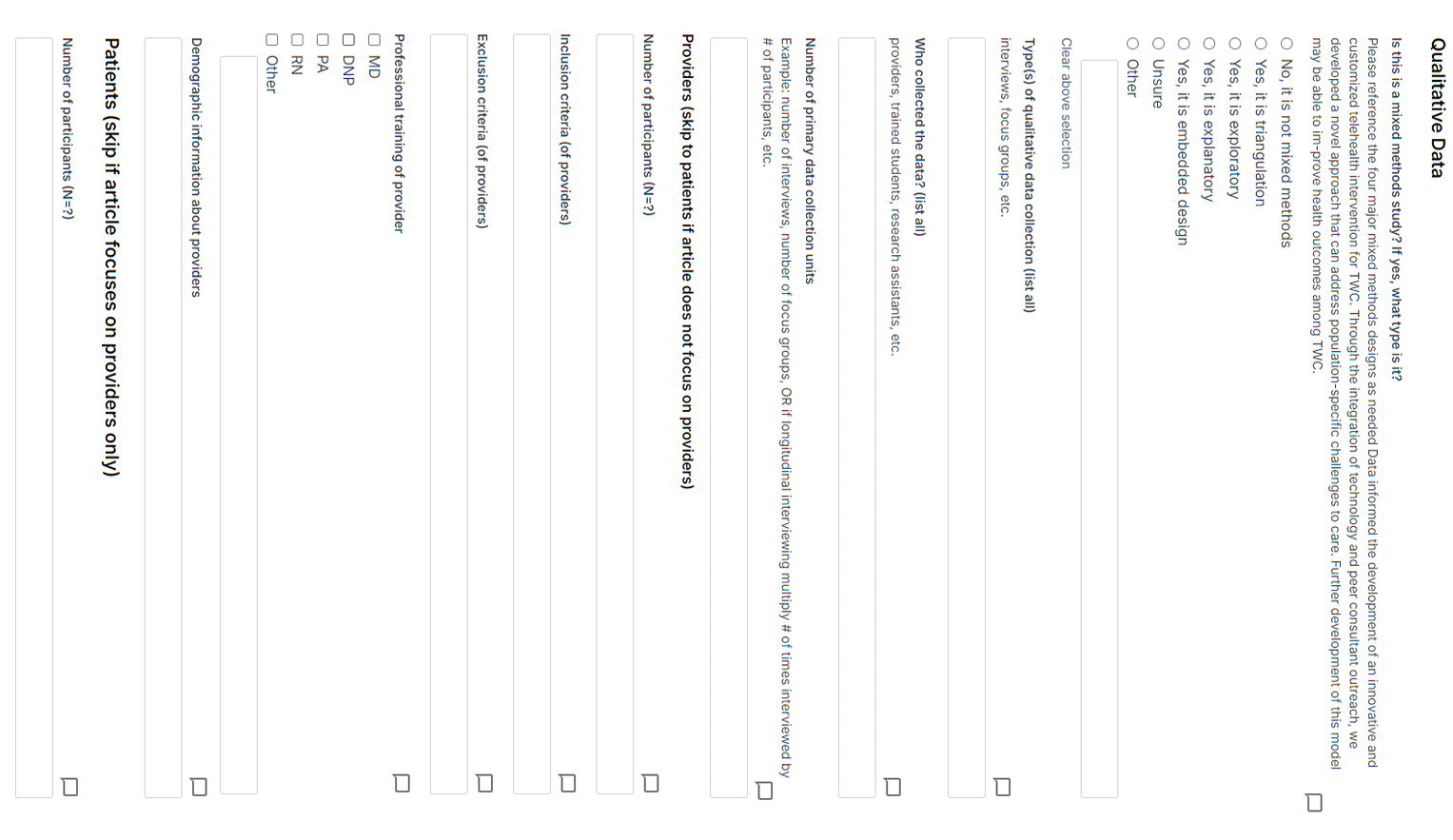

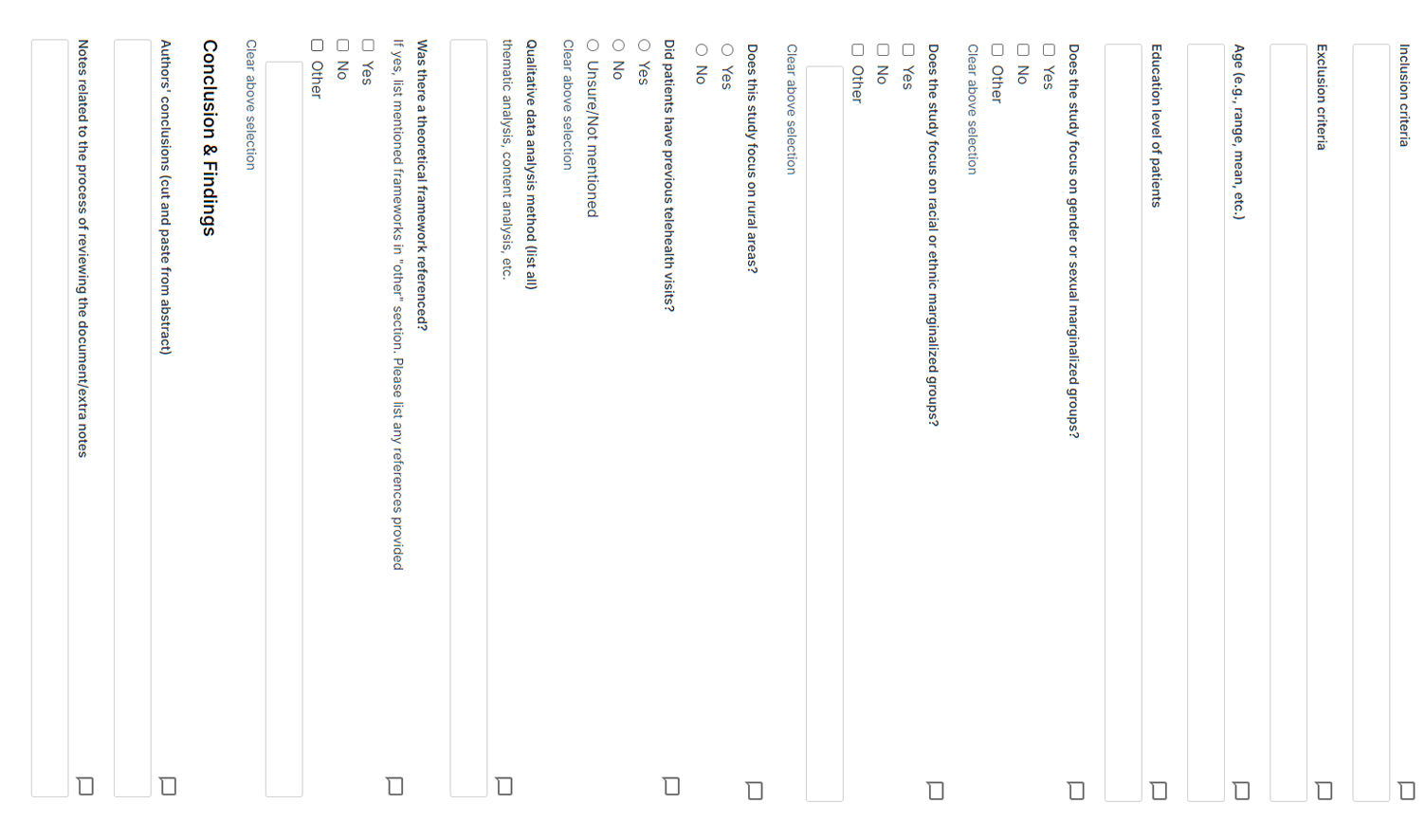

Supplement: Multimedia Appendix 3 [file jmir_v27i1e68314_app3.docx]
