# Supplementary material for: Experiences Receiving and Delivering Virtual Health Care For Women: Qualitative Evidence Synthesis
Source: J Med Internet Res. 2025 May 15;27:e68314. doi: 10.2196/68314 (PMC12123244; doi:10.2196/68314)
Supplement: Multimedia Appendix 4 [file jmir_v27i1e68314_app4.docx]

| **Citation** | **Exclusion reason** |
| --- | --- |
| Abedian, 2015^1^ | 1 |
| Abraham, 2017^2^ | 6 |
| Adams, 2023^3^ | 2 |
| Addington, 2018^4^ | 6 |
| Aiken, 2018^5^ | 3 |
| Aiken, 2018^5^ | 3 |
| Aiken, 2018^5^ | 6 |
| Altman, 2023^6^ | 6 |
| Anderson, 2021^7^ | 3 |
| Andrejek, 2021^8^ | 3 |
| Arias, 2022^9^ | 7 |
| Arthur, 2022^10^ | 2 |
| Asadisarvestani, 2024^11^ | 1 |
| Asano, 2021^12^ | 1 |
| Bailey, 2022^13^ | 5 |
| Baldwin, 2022^14^ | 6 |
| Balk, 2022^15^ | 7 |
| Barnicot, 2023^16^ | 6 |
| Baron, 2018^17^ | 3 |
| Bassilios, 2014^18^ | 2 |
| Bauer, 2021^19^ | 2 |
| Bazzano, 2024^20^ | 2 |
| Beamish, 2023^21^ | 2 |
| Ben-Arye, 2021^22^ | 2 |
| Bennett, 2022^23^ | 2 |
| Binda, 2022^24^ | 4 |
| Bittleston, 2022^25^ | 4 |
| Björk, 2014^26^ | 3 |
| Blaney, 2021^27^ | 2 |
| BoeDanbjørg, 2014^28^ | 4 |
| Bonsignore, 2018^29^ | 3 |
| Boran, 2023^30^ | 4 |
| BosoPerez, 2023^31^ | 2 |
| Boucher, 2022^32^ | 2 |
| Boucher, 2023^33^ | 2 |
| Boughton, 2016^34^ | 3 |
| Bracke, 2021^35^ | 3 |
| Bradbury, 2011^36^ | 4 |
| Braund, 2023^37^ | 2 |
| Bright, 2022^38^ | 4 |
| Brown, 2013^39^ | 7 |
| Cairns, 2023^40^ | 3 |
| Chakkalackal, 2021^41^ | 2 |
| Challacombe, 2023^42^ | 6 |
| Challacombe, 2024^43^ | 3 |
| Cibralic, 2023^44^ | 3 |
| Cluxton-Keller, 2018^45^ | 4 |
| Costanzo, 2022^46^ | 2 |
| Costanzo, 2023^47^ | 6 |
| Cruickshank, 2018^48^ | 7 |
| Dainty, 2023^49^ | 2 |
| DeKort, 2021^50^ | 6 |
| deSouza, 2023^51^ | 1 |
| Dol, 2022^52^ | 6 |
| Donnelly, 2013^53^ | 3 |
| Douglas, 2023^54^ | 6 |
| Drake, 2014^55^ | 6 |
| Ee, 2018^56^ | 6 |
| Emard, 2021^57^ | 3 |
| Ernesater, 2012^58^ | 2 |
| Evans, 2017^59^ | 4 |
| Evans, 2021^60^ | 6 |
| Faucher, 2020^61^ | 6 |
| FernandezLopez, 2022^62^ | 3 |
| Fiastro, 2022^63^ | 3 |
| Fitzke, 2023^64^ | 6 |
| Fitzsimmons, 2016^65^ | 2 |
| Fletcher, 2022^66^ | 2 |
| Fogarty, 2022^67^ | 2 |
| Footman, 2023^68^ | 6 |
| Freeman, 2023^69^ | 9 |
| Gagne, 2022^70^ | 6 |
| Gallegos, 2018^71^ | 4 |
| Garcia-Roca, 2022^72^ | 3 |
| Garne, 2016^73^ | 2 |
| Gaudine, 2023^74^ | 2 |
| Geraghty, 2020^75^ | 2 |
| Given, 2015^76^ | 6 |
| Godfrey, 2021^77^ | 6 |
| Gopal, 2022^78^ | 4 |
| Gray, 2015^79^ | 3 |
| Green, 2012^80^ | 6 |
| Green, 2015^81^ | 6 |
| Hanson, 2020^82^ | 2 |
| Hinton, 2022^83^ | 6 |
| Ho, 2022^84^ | 2 |
| Ho, 2023^85^ | 2 |
| Hoddinott, 2012^86^ | 3 |
| Homburg, 2022^87^ | 6 |
| Hood, 2023^45^ | 2 |
| Horn, 2023^88^ | 6 |
| Horwood, 2020^89^ | 2 |
| Hubbard, 2018^90^ | 3 |
| Hukku, 2022^91^ | 3 |
| Hunter-Jones, 2021^92^ | 4 |
| Im, 2021^3^ | 3 |
| Islam, 2023^93^ | 1 |
| Iyer, 2023^94^ | 2 |
| Jacobs, 2021^95^ | 6 |
| Johannessen, 2019^96^ | 2 |
| Johnson, 2021^97^ | 6 |
| Katapodi, 2018^98^ | 3 |
| Katayama, 2020^99^ | 3 |
| Kemp, 2018^100^ | 6 |
| Khalil, 2019^101^ | 6 |
| Killinger, 2022^102^ | 4 |
| Kloer, 2022^103^ | 6 |
| Ladin, 2021^104^ | 2 |
| LaRoche, 2020^105^ | 3 |
| Larrea, 2022^106^ | 6 |
| Laur, 2022^107^ | 2 |
| Lew, 2023^108^ | 6 |
| Lewis, 2021^109^ | 2 |
| Lindsay, 2017^110^ | 2 |
| Lipman, 2011^111^ | 6 |
| Lopez, 2021^112^ | 4 |
| Lubberding, 2015^113^ | 2 |
| Lunt, 2021^114^ | 2 |
| Lynch, 2012^115^ | 9 |
| Mabragana, 2013^116^ | 3 |
| MacLeod, 2023^117^ | 6 |
| Macnab, 2012^118^ | 7 |
| Madera, 2022^119^ | 6 |
| Madore, 2014^120^ | 3 |
| Magnus, 2020^121^ | 3 |
| Marhefka, 2012^122^ | 6 |
| Mary, 2021^123^ | 3 |
| Mattocks, 2017^124^ | 3 |
| Mazza, 2021^125^ | 6 |
| McHale, 2023^126^ | 4 |
| McKay, 2023^127^ | 6 |
| Mehl, 2022^128^ | 6 |
| Mendes-Santos, 2022^129^ | 6 |
| Meropol, 2011^130^ | 2 |
| Meyers, 2022^131^ | 6 |
| Mintz, 2022^132^ | 9 |
| Moin, 2015^133^ | 3 |
| Morgan, 2022^134^ | 2 |
| Morony, 2017^135^ | 3 |
| Neil-Sztramko, 2017^136^ | 9 |
| Newman, 2022^137^ | 2 |
| Newman, 2022^137^ | 2 |
| Ngai, 2019^138^ | 1 |
| Nguyen, 2022^139^ | 6 |
| Nicholson, 2016^140^ | 6 |
| Nishi, 2022^141^ | 3 |
| O'Brien, 2013^142^ | 6 |
| O'Neill, 2023^143^ | 2 |
| Özümerzifon, 2022^144^ | 3 |
| Painter, 2021^145^ | 2 |
| Penny, 2018^146^ | 4 |
| Pereira, 2023^147^ | 1 |
| Pierce, 2015^148^ | 2 |
| Pierre-Wright, 2023^149^ | 6 |
| Poon, 2022^150^ | 1 |
| Power, 2022^151^ | 3 |
| Raad, 2023^152^ | 2 |
| Rasekaba, 2016^153^ | 4 |
| Rasekaba, 2021^154^ | 3 |
| Rezel-Potts, 2020^155^ | 3 |
| Rissel, 2019^156^ | 6 |
| Rousseau, 2022^157^ | 3 |
| Saad, 2021^158^ | 6 |
| Saraiya, 2020^159^ | 6 |
| Schmidt-Hantke, 2023^160^ | 6 |
| Schoebel, 2021^161^ | 2 |
| Sedlander, 2018^162^ | 2 |
| Seward, 2018^163^ | 3 |
| Shah, 2015^164^ | 2 |
| Shaw, 2016^165^ | 3 |
| Shroder, 2018^166^ | 4 |
| Simblett, 2019^167^ | 2 |
| Simons, 2022^168^ | 2 |
| Simony, 2023^169^ | 6 |
| Singla, 2020^170^ | 6 |
| Skolasky, 2022^171^ | 2 |
| Slightam, 2023^172^ | 2 |
| Smith, 2021^173^ | 4 |
| Snaith, 2015^174^ | 3 |
| Spiby, 2019^175^ | 6 |
| Spiess, 2023^176^ | 2 |
| Sun, 2023^177^ | 2 |
| Sundstrom, 2019^178^ | 3 |
| Sundstrom, 2019^179^ | 3 |
| Talal, 2022^180^ | 2 |
| Talal, 2023^181^ | 3 |
| Talati, 2021^182^ | 7 |
| Talati, 2021^183^ | 7 |
| Tavener, 2022^184^ | 3 |
| Taylor, 2019^185^ | 2 |
| Thawani, 2022^186^ | 2 |
| Therouanne, 2023^187^ | 6 |
| Thielde Bocanegra, 2022^188^ | 6 |
| Truong, 2023^189^ | 2 |
| Tutty, 2019^190^ | 4 |
| Uscher-Pines, 2022^191^ | 7 |
| Uskup, 2022^192^ | 4 |
| VanErkel, 2022^193^ | 6 |
| VanGerwen, 2023^194^ | 6 |
| vanIerssel, 2023^195^ | 2 |
| vonBelow, 2023^196^ | 2 |
| Walker, 2018^197^ | 3 |
| White, 2022^198^ | 2 |
| Wisner, 2017^199^ | 9 |
| Wit, 2021^200^ | 6 |
| Woods, 2023^201^ | 6 |
| Wyatt, 2013^202^ | 6 |
| Yelverton, 2023^203^ | 2 |
| Zaccari, 2022^204^ | 4 |
| Zilliacus, 2010^205^ | 6 |

Exclusion reasons: 1 = Non OECD; 2 = Wrong population; 3 = Wrong evaluation; 4 = Wrong study design; 5 = Wrong language; 6 = Wrong phenomena of interest; 7 = Wrong publication type; 8 = Wrong years; 9 = Wrong research type; 10 = Wrong setting

**REFERENCES**

1. Abedian Z, Abbaszadeh N, Roudsari RL, Shakeri MT. The Effects of Telephone Support on Stress and Perceived Social Support in Primiparous Women Experiencing Nausea and Vomiting in the First Half of Pregnancy. *Journal of Midwifery & Reproductive Health.* 2015;3(2):328-334.

2. Abraham TH, Cucciare MA, White P, Booth BM, Wright P. Feasibility and acceptability of shared decision-making to promote alcohol behavior change among women Veterans: Results from focus groups. *Journal of Addictive Diseases.* 2017;36(4):252-263.

3. Adams AM, Williams KKA, Langill JC, et al. Telemedicine perceptions and experiences of socially vulnerable households during the early stages of the COVID-19 pandemic: a qualitative study. *CMAJ Open.* 2023;11(2):E219-E226.

4. Addington EL, Sohl SJ, Tooze JA, Danhauer SC. Convenient and Live Movement (CALM) for women undergoing breast cancer treatment: Challenges and recommendations for internet-based yoga research. *Complementary Therapies in Medicine.* 2018;37:77-79.

5. Aiken ARA, Broussard K, Johnson DM, Padron E. Motivations and Experiences of People Seeking Medication Abortion Online in the United States. *Perspectives on Sexual & Reproductive Health.* 2018;50(4):157-163.

6. Altman MR, Mohammed SA, Eagen-Torkko MK, Kantrowitz-Gordon I, Gavin AR. Losing Connection: Experiences of Virtual Pregnancy and Postpartum Care During the COVID-19 Pandemic. *J Perinat Neonatal Nurs.* 2023;37(1):44-49.

7. Anderson D, Sturt J, McDonald N, et al. Women's Wellness with Type 2 Diabetes Program (WWDP): Qualitative findings from the UK and Australian feasibility study. *Diabetes Research & Clinical Practice.* 2021;172:108654.

8. Andrejek N, Hossain S, Schoueri-Mychasiw N, et al. Barriers and Facilitators to Resuming In-Person Psychotherapy with Perinatal Patients amid the COVID-19 Pandemic: A Multistakeholder Perspective. *International Journal of Environmental Research & Public Health [Electronic Resource].* 2021;18(22):22.

9. Arias MP, Wang EY, Hamm RF, et al. IMPACT (IMpact on PostpArtum Care by Telehealth) Study: a qualitative evaluation of the patient perspective. *American Journal of Obstetrics & Gynecology.* 2022;226(1):S204-S204.

10. Arthur EK, Pisegna J, Oliveri JM, Aker H, Krok-Schoen JL. Older cancer survivors' perspectives and use of telehealth in their cancer survivorship care in the United States: A ResearchMatch R sample. *J Geriatr Oncol.* 2022;13(8):1223-1229.

11. Asadisarvestani K, Hulsbergen M. Experiences with family planning and abortion services during the Covid-19 pandemic: a qualitative study in Bangladesh, Iran and Netherlands. *BMC Public Health.* 2024;24(1):31.

12. Asano M, Koh GCH, Madhukumar P, et al. Study protocol: a pilot quasi-experimental trial of tele-rehabilitation and tele-drain care post-mastectomy. *Pilot and Feasibility Studies.* 2021;7(1).

13. Bailey C, Weller-Newton J, Hall H. O67 - Telephone triage in midwifery practice: a mixed methods study. *Women & Birth.* 2022;35:N.PAG-N.PAG.

14. Baldwin A, Johnson DM, Broussard K, et al. U.S. Abortion Care Providers' Perspectives on Self-Managed Abortion. *Qualitative Health Research.* 2022;32(5):788-799.

15. Balk EM, Konnyu KJ, Cao W, et al. *Agency for Healthcare Research and Quality.* 2022:06.

16. Barnicot K, Parker J, Kalwarowsky S, et al. Mother and clinician experiences of a trial of a video feedback parent-infant intervention for mothers experiencing difficulties consistent with 'personality disorder': A qualitative interview study. *Psychol Psychother.* 2023;96(2):480-503.

17. Baron AM, Ridgeway JL, Finnie DM, et al. Increasing the Connectivity and Autonomy of RNs with Low-Risk Obstetric Patients: Findings of a study exploring the use of a new prenatal care model. *AJN American Journal of Nursing.* 2018;118(1):48-55.

18. Bassilios B, Pirkis J, King K, Fletcher J, Blashki G, Burgess P. Evaluation of an Australian primary care telephone cognitive behavioural therapy pilot. *Australian Journal of Primary Health.* 2014;20(1):62-73.

19. Bauer A, Amspoker AB, Fletcher TL, et al. A Resource Building Virtual Care Programme: improving symptoms and social functioning among female and male rural veterans. *European Journal of Psychotraumatology.* 2021;12(1):1860357.

20. Bazzano AN, Patel T, Nauman E, Cernigliaro D, Shi L. Optimizing Telehealth for Diabetes Management in the Deep South of the United States: Qualitative Study of Barriers and Facilitators on the Patient and Clinician Journey. *J Med Internet Res.* 2024;26:e43583.

21. Beamish P, McNeill K, Arnaout A, Malcolm J. Patient Perspectives on Virtual Care for Diabetes Management in the Era of COVID-19. *Can.* 2023;47(8):636-642.

22. Ben-Arye E, Keshet Y, Gressel O, Tapiro Y, Lavie O, Samuels N. Being in touch: narrative assessment of patients receiving online integrative oncology treatments during COVID-19. *Supportive Care in Cancer.* 2021;29(8):4819-4825.

23. Bennett SE, Almeida C, Bachmair EM, et al. Therapists' experiences of remotely delivering cognitive-behavioural or graded-exercise interventions for fatigue: a qualitative evaluation. *Rheumatol.* 2022;6(3):rkac083.

24. Binda V, Olhaberry M, Castanon C, Abarca C, Caamano C. A Group Videoconferencing Intervention (C@nnected) to Improve Maternal Sensitivity: Protocol for a Randomized Feasibility Trial. *JMIR Research Protocols.* 2022;11(8):e35881.

25. Bittleston H, Goller JL, Temple-Smith M, Hocking JS, Coombe J. Telehealth for sexual and reproductive health issues: a qualitative study of experiences of accessing care during COVID-19. *Sexual Health.* 2022;23:23.

26. Björk A-B, Sjöström M, Johansson EE, Samuelsson E, Umefjord G. Women’s Experiences of Internet-Based or Postal Treatment for Stress Urinary Incontinence. *Qualitative Health Research.* 2014;24(4):484-493.

27. Blaney C, Hitchon CA, Marrie RA, Mackenzie C, Holens P, El-Gabalawy R. Support for a non-therapist assisted, Internet-based cognitive-behavioral therapy (iCBT) intervention for mental health in rheumatoid arthritis patients. *Internet Interventions.* 2021;24:100385.

28. Boe Danbjørg D, Wagner L, Clemensen J. Designing, Developing, and Testing an App for Parents Being Discharged Early Postnatally. *Journal for Nurse Practitioners.* 2014;10(10):794-802.

29. Bonsignore L, Bloom N, Steinhauser K, et al. Evaluating the Feasibility and Acceptability of a Telehealth Program in a Rural Palliative Care Population: TapCloud for Palliative Care. *Journal of Pain & Symptom Management.* 2018;56(1):7-14.

30. Boran P, Donmez M, Baris E, et al. Delivering the Thinking Healthy Programme as a universal group intervention integrated into routine antenatal care: a randomized-controlled pilot study. *BMC Psychiatry.* 2023;23(1):14.

31. Boso Perez R, Reid D, Maxwell KJ, et al. Access to and quality of sexual and reproductive health services in Britain during the early stages of the COVID-19 pandemic: a qualitative interview study of patient experiences. *BMJ sex.* 2023;49(1):12-20.

32. Boucher AA, Jewett PI, Holtan SG, Lindgren BR, Hui JYC, Blaes AH. Adult Hematology/Oncology Patient Perspectives on Telemedicine Highlight Areas of Focus for Future Hybrid Care Models. *Telemedicine Journal & E Health.* 2022;03:03.

33. Boucher AA, Jewett PI, Holtan SG, Lindgren BR, Hui JYC, Blaes AH. Adult Hematology/Oncology Patient Perspectives on Telemedicine Highlight Areas of Focus for Future Hybrid Care Models. *Telemed J E Health.* 2023;29(5):708-716.

34. Boughton RR, Jindani F, Turner NE. Group Treatment for Women Gamblers Using Web, Teleconference and Workbook: Effectiveness Pilot. *International Journal of Mental Health & Addiction.* 2016;14(6):1074-1095.

35. Bracke X, Roberts J, McVeigh TP. A systematic review and meta-analysis of telephone vs in-person genetic counseling in BRCA1/BRCA2 genetic testing. *Journal of Genetic Counseling.* 2021;30(2):563-573.

36. Bradbury AR, Patrick-Miller L, Fetzer D, et al. Genetic counselor opinions of, and experiences with telephone communication of BRCA1/2 test results. *Clinical Genetics.* 2011;79(2):125-131.

37. Braund H, Dalgarno N, Chan-Nguyen S, et al. Exploring Patient Advisors' Perceptions of Virtual Care Across Canada: Qualitative Phenomenological Study. *J Med Internet Res.* 2023;25:e45215.

38. Bright KS, Stuart S, McNeil DA, Murray L, Kingston DE. Feasibility and Acceptability of Internet-Based Interpersonal Psychotherapy for Stress, Anxiety, and Depression in Prenatal Women: Thematic Analysis. *JMIR Formative Research.* 2022;6(6):e23879.

39. Brown S, Findlay D. Maternity Telehealth in Scotland. *Midwifery Matters.* 2013(138):7-8.

40. Cairns AJJ, Kelly J, Taylor CDJ. Assessing the delivering of iMAgery-focused therapy for PSychosis (iMAPS) via telehealth. *Psychol Psychother.* 2023;96(3):678-696.

41. Chakkalackal L, Rosan C, Corfield F, et al. A mixed-method evaluation of video interaction guidance (VIG) delivered by early-years workers in a socially disadvantaged urban community. *Journal of Mental Health Training, Education & Practice.* 2021;16(5):396-409.

42. Challacombe FL, Sabin K, Jacobson S, et al. Patient and therapist experiences of exposure therapy for anxiety-related disorders in pregnancy: qualitative analysis of a feasibility trial of intensive versus weekly CBT. *BJPsych Open.* 2023;9(6):e189.

43. Challacombe FL, Tinch-Taylor R, Sabin K, et al. Exposure-based cognitive-behaviour therapy for anxiety-related disorders in pregnancy (ADEPT): Results of a feasibility randomised controlled trial of time-intensive versus weekly CBT. *J Affect Disord.* 2024;344:414-422.

44. Cibralic S, Fay-Stammbach T, Tucker D, Song D, Eapen V. A mixed-methods evaluation of a state-wide outreach perinatal mental health service. *BMC Pregnancy Childbirth.* 2023;23(1):74.

45. Cluxton-Keller F, Williams M, Buteau J, et al. Video-Delivered Family Therapy for Home Visited Young Mothers With Perinatal Depressive Symptoms: Quasi-Experimental Implementation-Effectiveness Hybrid Trial. *JMIR Mental Health.* 2018;5(4):e11513.

46. Costanzo S, De Summa S, Maurmo L, Digennaro M, Patruno M, Paradiso A. Remote vs in-person BRCA1/2 non-carriers test disclosure: patients' choice during Covid-19 pandemic restriction. *Familial Cancer.* 2022;22:22.

47. Costanzo S, De Summa S, Maurmo L, Digennaro M, Patruno M, Paradiso A. Remote vs in-person BRCA1/2 non-carriers test disclosure: patients' choice during Covid-19 pandemic restriction. *Fam Cancer.* 2023;22(1):43-48.

48. Cruickshank S, Steel E, Fenlon D, et al. A feasibility study of the Mini-AFTER telephone intervention for the management of fear of recurrence in breast cancer survivors: a mixed-methods study protocol. *Pilot & Feasibility Studies.* 2018;4:22.

49. Dainty KN, Seaton MB, Hall JN, et al. "It saved me from the emergency department": A qualitative study of patient experience of virtual urgent care in Ontario. *PLoS ONE.* 2023;18(9):e0285468.

50. De Kort L, Wouters E, Van de Velde S. Obstacles and opportunities: a qualitative study of the experiences of abortion centre staff with abortion care during the first COVID-19 lockdown in Flanders, Belgium. *Sexual and Reproductive Health Matters.* 2021;29(1).

51. de Souza J, Calsinski C, Chamberlain K, Cibrian F, Wang EJ. Investigating interactive methods in remote chestfeeding support for lactation consulting professionals in Brazil. *Front.* 2023;5:1143528.

52. Dol J, Aston M, Grant A, McMillan D, Tomblin Murphy G, Campbell-Yeo M. Implementing Essential Coaching for Every Mother during the COVID-19 pandemic: A pre-post intervention study. *Birth.* 2022;49(2):273-280.

53. Donnelly CM, Lowe-Strong A, Rankin JP, Campbell A, Blaney JM, Gracey JH. A focus group study exploring gynecological cancer survivors' experiences and perceptions of participating in a RCT testing the efficacy of a home-based physical activity intervention. *Supportive Care in Cancer.* 2013;21(6):1697-1708.

54. Douglas TC, May C, Dent K, Carey JC, Mladucky J. Prenatal patient perceptions of receiving difficult news over the telephone. *J Genet Couns.* 2023;32(4):857-869.

55. Drake E, Howard E, Kinsey E. Online screening and referral for postpartum depression: an exploratory study. *Community mental health journal.* 2014;50(3):305-311.

56. Ee C, Smith C, Costello M, et al. Feasibility and acceptability of a proposed trial of acupuncture as an adjunct to lifestyle interventions for weight loss in Polycystic Ovary Syndrome: a qualitative study. *BMC Complementary & Alternative Medicine.* 2018;18(1):298.

57. Emard N, Lynch KA, Liou KT, et al. Virtual Mind-Body Programming for Patients With Cancer During the COVID-19 Pandemic: Qualitative Study. *JMIR Cancer.* 2021;7(2):e27384.

58. Ernesater A, Winblad U, Engstrom M, Holmstrom IK. Malpractice claims regarding calls to Swedish telephone advice nursing: what went wrong and why? *Journal of Telemedicine & Telecare.* 2012;18(7):379-383.

59. Evans EC, Deutsch NL, Drake E, Bullock L. Nurse-Patient Interaction as a Treatment for Antepartum Depression: A Mixed-Methods Analysis [Formula: see text]. *Journal of the American Psychiatric Nurses Association.* 2017;23(5):347-359.

60. Evans S, Dowding C, Druitt M, Mikocka-Walus A. "I'm in iso all the time anyway": A mixed methods study on the impact of COVID-19 on women with endometriosis. *Journal of Psychosomatic Research.* 2021;146:110508.

61. Faucher MA, Kennedy HP. Women's Perceptions on the Use of Video Technology in Early Labor: Being Able to See. *Journal of Midwifery & Women's Health.* 2020;65(3):342-348.

62. Fernandez Lopez R, de-Leon-de-Leon S, Martin-de-Las-Heras S, Torres Cantero JC, Megias JL, Zapata-Calvente AL. Women survivors of intimate partner violence talk about using e-health during pregnancy: a focus group study. *BMC Women's Health.* 2022;22(1):98.

63. Fiastro AE, Sanan S, Jacob-Files E, et al. Remote Delivery in Reproductive Health Care: Operation of Direct-to-Patient Telehealth Medication Abortion Services in Diverse Settings. *Annals of Family Medicine.* 2022;20(4):336-342.

64. Fitzke RE, Bouskill KE, Sedano A, et al. Barriers and Facilitators to Behavioral Healthcare for Women Veterans: a Mixed-Methods Analysis of the Current Landscape. *J Behav Health Serv Res.* 2023;05:05.

65. Fitzsimmons DA, Thompson J, Bentley CL, Mountain GA. Comparison of patient perceptions of Telehealth-supported and specialist nursing interventions for early stage COPD: a qualitative study. *BMC Health Services Research.* 2016;16(1):420.

66. Fletcher TL, Amspoker AB, Wassef M, et al. Increasing access to care for trauma-exposed rural veterans: A mixed methods outcome evaluation of a web-based skills training program with telehealth-delivered coaching. *Journal of Rural Health.* 2022;38(4):740-747.

67. Fogarty A, Savopoulos P, Seymour M, et al. Providing therapeutic services to women and children who have experienced intimate partner violence during the COVID-19 pandemic: Challenges and learnings. *Child Abuse & Neglect.* 2022;130(Pt 1):105365.

68. Footman K. Revolution in abortion care? Perspectives of key informants on the importance of abortion method choice in the era of telemedicine. *Sex.* 2023;31(1):2149379.

69. Freeman JQ, Khwaja A, Zhao F, Nanda R, Olopade OI, Huo D. Racial/Ethnic Disparities in Telemedicine Utilization and Satisfaction Among Breast Cancer Patients During the COVID-19 Pandemic: A Mixed-Methods Analysis. *Telemed J E Health.* 2023;07:07.

70. Gagne M, Lauzier S, Lemay M, et al. Women with breast cancer's perceptions of nurse-led telephone-based motivational interviewing consultations to enhance adherence to adjuvant endocrine therapy: a qualitative study. *Supportive Care in Cancer.* 2022;30(6):4759-4768.

71. Gallegos D, Cromack C, Thorpe KJ. Can a phone call make a difference? Breastfeeding self-efficacy and nurse responses to mother's calls for help. *Journal of Child Health Care.* 2018;22(3):433-446.

72. Garcia-Roca ME, Rodriguez-Arrastia M, Ropero-Padilla C, et al. Breast Cancer Patients' Experiences with Online Group-Based Physical Exercise in a COVID-19 Context: A Focus Group Study. *Journal of Personalized Medicine.* 2022;12(3):26.

73. Garne K, Brodsgaard A, Zachariassen G, Clemensen J. Telemedicine in Neonatal Home Care: Identifying Parental Needs Through Participatory Design. *JMIR Research Protocols.* 2016;5(3):e100.

74. Gaudine A, Parsons K, Smith-Young J. Older Adults' Experiences with Remote Care for Specialized Health Service During the COVID-19 Pandemic: A Descriptive Qualitative Study. *Can J Aging.* 2023:1-9.

75. Geraghty AWA, Roberts LC, Stanford R, et al. Exploring Patients' Experiences of Internet-Based Self-Management Support for Low Back Pain in Primary Care. *Pain Medicine.* 2020;21(9):1806-1817.

76. Given JE, Bunting BP, O'Kane MJ, Dunne F, Coates VE. Tele-Mum: A Feasibility Study for a Randomized Controlled Trial Exploring the Potential for Telemedicine in the Diabetes Care of Those with Gestational Diabetes. *Diabetes Technology & Therapeutics.* 2015;17(12):880-888.

77. Godfrey EM, Fiastro AE, Jacob-Files EA, et al. Factors associated with successful implementation of telehealth abortion in 4 United States clinical practice settings. *Contraception.* 2021;104(1):82-91.

78. Gopal A, Bonanno V, Block VJ, Bove RM. Accessibility to Telerehabilitation Services for People With Multiple Sclerosis: Analysis of Barriers and Limitations. *Int.* 2022;24(6):260-265.

79. Gray MJ, Hassija CM, Jaconis M, et al. Provision of Evidence-Based Therapies to Rural Survivors of Domestic Violence and Sexual Assault via Telehealth: Treatment Outcomes and Clinical Training Benefits. *Training & Education in Professional Psychology.* 2015;9(3):235-241.

80. Green JM, Spiby H, Hucknall C, Richardson Foster H. Converting policy into care: women's satisfaction with the early labour telephone component of the All Wales Clinical Pathway for Normal Labour. *Journal of Advanced Nursing.* 2012;68(10):2218-2228.

81. Green SM, Lockhart E, Marhefka SL. Advantages and disadvantages for receiving Internet-based HIV/AIDS interventions at home or at community-based organizations. *AIDS Care.* 2015;27(10):1304-1308.

82. Hanson JD, Weber TL, Shrestha U, Bares VJ, Seiber M, Ingersoll K. Acceptability of an eHealth Intervention to Prevent Alcohol-Exposed Pregnancy Among American Indian/Alaska Native Teens. *Alcoholism: Clinical & Experimental Research.* 2020;44(1):196-202.

83. Hinton L, Dakin FH, Kuberska K, et al. Quality framework for remote antenatal care: qualitative study with women, healthcare professionals and system-level stakeholders. *BMJ Quality & Safety.* 2022;12:12.

84. Ho JSS, Leclair R, Braund H, et al. Transitioning to virtual ambulatory care during the COVID-19 pandemic: a qualitative study of faculty and resident physician perspectives. *CMAJ open.* 2022;10(3):E762-E771.

85. Ho TF, Fortenberry KT, Gardner E, et al. Perceived Impact of Virtual Visits on Access to Care in Family Medicine During the COVID-19 Pandemic: A Qualitative Study of Benefits and Challenges. *J.* 2023;14:21501319231220118.

86. Hoddinott P, Craig L, MacLennan G, Boyers D, Vale L. Process evaluation for the FEeding Support Team (FEST) randomised controlled feasibility trial of proactive and reactive telephone support for breastfeeding women living in disadvantaged areas. *BMJ Open.* 2012;2(2).

87. Homburg M, Brandenbarg D, Olde Hartman T, et al. Patient experiences during the COVID-19 pandemic: a qualitative study in Dutch primary care. *BJGP open.* 2022;6(4).

88. Horn CE, Seely EW, Levkoff SE, Isley BC, Nicklas JM. Postpartum women's experiences in a randomized controlled trial of a web-based lifestyle intervention following Gestational Diabetes: a qualitative study. *J Matern Fetal Neonatal Med.* 2023;36(1):2194012.

89. Horwood J, Brangan E, Manley P, et al. Management of chlamydia and gonorrhoea infections diagnosed in primary care using a centralised nurse-led telephone-based service: mixed methods evaluation. *BMC Family Practice.* 2020;21(1):265.

90. Hubbard G, Campbell A, Fisher A, et al. Physical activity referral to cardiac rehabilitation, leisure centre or telephonedelivered consultations in post-surgical people with breast cancer: A mixed methods process evaluation. *Pilot and Feasibility Studies.* 2018;4(1).

91. Hukku S, Menard A, Kemzang J, Hastings E, Foster AM. "I just was really scared, because it's already such an uncertain time": Exploring women's abortion experiences during the COVID-19 pandemic in Canada. *Contraception.* 2022;110:48-55.

92. Hunter-Jones J, Gilliam S, Davis C, et al. Process and Outcome Evaluation of a Mindfulness-Based Cognitive Therapy Intervention for Cisgender and Transgender African American Women Living with HIV/AIDS. *AIDS and behavior.* 2021;25(2):592-603.

93. Islam A, Begum F, Williams A, Basri R, Ara R, Anderson R. Midwife-led pandemic telemedicine services for maternal health and gender-based violence screening in Bangladesh: an implementation research case study. *Reprod Health.* 2023;20(1):128.

94. Iyer SS, Ngo V, Humber MB, et al. Caregiver Experience of Tele-dementia Care for Older Veterans. *J Gen Intern Med.* 2023;38(13):2960-2969.

95. Jacobs JM, Walsh EA, Rapoport CS, et al. Development and Refinement of a Telehealth Intervention for Symptom Management, Distress, and Adherence to Adjuvant Endocrine Therapy after Breast Cancer. *Journal of Clinical Psychology in Medical Settings.* 2021;28(3):603-618.

96. Johannessen TB, Storm M, Holm AL. Safety for older adults using telecare: Perceptions of homecare professionals. *Nursing Open.* 2019;6(3):1254-1261.

97. Johnson DM, Madera M, Gomperts R, Aiken ARA. The economic context of pursuing online medication abortion in the United States. *SSM Qualitative Research in Health.* 2021;1.

98. Katapodi MC, Jung M, Schafenacker AM, et al. Development of a Web-based Family Intervention for BRCA Carriers and Their Biological Relatives: Acceptability, Feasibility, and Usability Study. *JMIR Cancer.* 2018;4(1):e7.

99. Katayama K, Ishikawa D, Miyagi Y, Takemiya S, Okamoto N, Ogawa A. Qualitative analysis of cancer telephone consultations: Differences in the counseling needs of Japanese men and women. *Patient Education and Counseling.* 2020;103(12):2555-2564.

100. Kemp E, Koczwara B, Butow P, et al. Online information and support needs of women with advanced breast cancer: a qualitative analysis. *Supportive Care in Cancer.* 2018;26(10):3489-3496.

101. Khalil C. Understanding the Adoption and Diffusion of a Telemonitoring Solution in Gestational Diabetes Mellitus: Qualitative Study. *JMIR Diabetes.* 2019;4(4):e13661.

102. Killinger K, Gunther S, Gomperts R, Atay H, Endler M. Why women choose abortion through telemedicine outside the formal health sector in Germany: a mixed-methods study. *BMJ Sexual & Reproductive Health.* 2022;48(e1):e6-e12.

103. Kloer C, Lewis HC, Rezak K. Delays in gender affirming healthcare due to COVID-19 are mitigated by expansion of telemedicine. *American Journal of Surgery.* 2022;23:23.

104. Ladin K, Porteny T, Perugini JM, et al. Perceptions of Telehealth vs In-Person Visits Among Older Adults With Advanced Kidney Disease, Care Partners, and Clinicians. *JAMA Network Open.* 2021;4(12):e2137193.

105. LaRoche KJ, Wynn LL, Foster AM. "We've got rights and yet we don't have access": Exploring patient experiences accessing medication abortion in Australia. *Contraception.* 2020;101(4):256-260.

106. Larrea S, Hidalgo C, Jacques-Aviñó C, Borrell C, Palència L. “No one should be alone in living this process”: trajectories, experiences and user’s perceptions about quality of abortion care in a telehealth service in Chile. *Sexual and Reproductive Health Matters.* 2022;29(3).

107. Laur C, Agarwal P, Thai K, et al. Implementation and Evaluation of COVIDCare@Home, a Family Medicine-Led Remote Monitoring Program for Patients With COVID-19: Multimethod Cross-sectional Study. *JMIR Human Factors.* 2022;9(2):e35091.

108. Lew E, Tan SFJ, Teo A, Sng BL, Lum EPM. Perceptions and Attitudes of Patients and Health Care Stakeholders on Implementing a Telehealth Service for Preoperative Evaluation: A Qualitative Analysis. *Telemed Rep.* 2023;4(1):156-165.

109. Lewis AK, Harding KE, Taylor NF, O'Brien TJ, Carney PW. The feasibility of delivering first suspected seizure care using telehealth: A mixed methods controlled study. *Epilepsy Research.* 2021;169:106520.

110. Lindsay JA, Hudson S, Martin L, et al. Implementing Video to Home to Increase Access to Evidence-Based Psychotherapy for Rural Veterans. *Journal of Technology in Behavioral Science.* 2017;2(3-4):140-148.

111. Lipman EL, Kenny M, Marziali E. Providing web-based mental health services to at-risk women. *BMC Women's Health.* 2011;11:38.

112. Lopez CJ, Edwards B, Langelier DM, Chang EK, Chafranskaia A, Jones JM. Delivering Virtual Cancer Rehabilitation Programming During the First 90 Days of the COVID-19 Pandemic: A Multimethod Study. *Archives of Physical Medicine and Rehabilitation.* 2021;102(7):1283-1293.

113. Lubberding S, Uden‐Kraan CF, Te Velde EA, Cuijpers P, Leemans CR, Verdonck‐de Leeuw IM. Improving access to supportive cancer care through an e Health application: a qualitative needs assessment among cancer survivors. *Journal of Clinical Nursing (John Wiley & Sons, Inc).* 2015;24(9-10):1367-1379.

114. Lunt A, Llewellyn C, Bayley J, Nadarzynski T. Sexual healthcare professionals' views on the rapid provision of remote services at the beginning of COVID-19 pandemic: A mixed-methods study. *International Journal of STD & AIDS.* 2021;32(12):1138-1148.

115. Lynch J, Gay S. A survey of telehealth coordinators in Canada. *Journal of Telemedicine & Telecare.* 2012;18(4):231-234.

116. Mabragana M, Carballo-Dieguez A, Giguere R. Young women's experience with using videoconferencing for the assessment of sexual behavior and microbicide use. *Telemedicine Journal & E-Health.* 2013;19(11):866-871.

117. MacLeod A, Aston M, Price S, et al. "There's an Etiquette to Zoom That's Not Really Present In-Person": A Qualitative Study Showing How the Mute Button Shapes Virtual Postpartum Support for New Parents. *Qual Health Res.* 2023;33(11):1005-1016.

118. Macnab I, Rojjanasrirat W, Sanders A. Breastfeeding and telehealth. *Journal of human lactation : official journal of International Lactation Consultant Association.* 2012;28(4):446-449.

119. Madera M, Johnson DM, Broussard K, et al. Experiences seeking, sourcing, and using abortion pills at home in the United States through an online telemedicine service. *SSM Qual Res Health.* 2022;2.

120. Madore S, Kilbourn K, Valverde P, Borrayo E, Raich P. Feasibility of a psychosocial and patient navigation intervention to improve access to treatment among underserved breast cancer patients. *Supportive Care in Cancer.* 2014;22(8):2085-2093.

121. Magnus M, Edwards E, Dright A, et al. Development of a telehealth intervention to promote care-seeking among transgender women of color in Washington, DC. *Public Health Nursing.* 2020;37(2):262-271.

122. Marhefka S, Fuhrmann H, Gilliam P, Lopez B, Baldwin J. Interest in, Concerns About, and Preferences for Potential Video-Group Delivery of an Effective Behavioral Intervention Among Women Living With HIV. *AIDS & Behavior.* 2012;16(7):1961-1969.

123. Mary M, Das P, Creanga AA. Perinatal telemedicine at lower-level birthing hospitals in Maryland. Lessons learned from a landscape analysis. *Minerva Obstetrics and Gynecology.* 2021;09:09.

124. Mattocks KM, Kuzdeba J, Baldor R, Casares J, Lombardini L, Gerber MR. Implementing and Evaluating a Telephone-Based Centralized Maternity Care Coordination Program for Pregnant Veterans in the Department of Veterans Affairs. *Women's Health Issues.* 2017;27(5):579-585.

125. Mazza D, Seymour JW, Sandhu MV, Melville C, O'Brien J, Thompson T-A. General practitioner knowledge of and engagement with telehealth-at-home medical abortion provision. *Australian Journal of Primary Health.* 2021;27(6):456-461.

126. McHale S, Paterson M, Pearsons A, et al. Exploring the patient experience of remote hypertension management in Scotland during COVID-19: a qualitative study. *BMJ Open.* 2023;13(12):e078944.

127. McKay E, Ojukwu E, Hirani S, Sotindjo T, Okedo-Alex I, Magagula P. How the COVID-19 Pandemic Influenced HIV Care: Are We Prepared Enough for Future Pandemics? An Assessment of Factors Influencing Access, Utilization, Affordability, and Motivation to Engage with HIV Services amongst African, Caribbean, and Black Women. *Int J Environ Res Public Health.* 2023;20(11):05.

128. Mehl SC, Short WD, Powell P, et al. Impact of Telemedicine on Prenatal Counseling at a Tertiary Fetal Center: A Mixed Methods Study. *Journal of Surgical Research.* 2022;280:288-295.

129. Mendes-Santos C, Nunes F, Weiderpass E, Santana R, Andersson G. Development and Evaluation of the Usefulness, Usability, and Feasibility of iNNOV Breast Cancer: Mixed Methods Study. *JMIR Cancer.* 2022;8(1).

130. Meropol NJ, Daly MB, Vig HS, et al. Delivery of internet-based cancer genetic counselling services to patients' homes: A feasibility study. *Journal of Telemedicine and Telecare.* 2011;17(1):36-40.

131. Meyers M, Margraf J, Velten J. A Qualitative Study of Women's Experiences with Cognitive-Behavioral and Mindfulness-Based Online Interventions for Low Sexual Desire. *Journal of Sex Research.* 2022:1-10.

132. Mintz LJ, Gillani B, Moore SE. Telehealth in Trans and Gender Diverse Communities: the Impact of COVID-19. *Current Obstetrics and Gynecology Reports.* 2022;11(2):75-80.

133. Moin T, Ertl K, Schneider J, et al. Women veterans' experience with a web-based diabetes prevention program: a qualitative study to inform future practice. *Journal of Medical Internet Research.* 2015;17(5):e127.

134. Morgan A, Davies C, Olabi Y, Hope-Stone L, Cherry MG, Fisher P. Therapists' experiences of remote working during the COVID-19 pandemic. *Front Psychol.* 2022;13:966021.

135. Morony S, Weir K, Duncan G, Biggs J, Nutbeam D, McCaffery K. Experiences of Teach-Back in a Telephone Health Service. *Health Literacy Research and Practice.* 2017;1(4):e173-e181.

136. Neil-Sztramko SE, Gotay CC, Sabiston CM, Demers PA, Campbell KC. Feasibility of a telephone and web-based physical activity intervention for women shift workers. *Translational Behavioral Medicine.* 2017;7(2):268-276.

137. Newman CE, Fraser D, Ong JJ, Bourne C, Grulich AE, Bavinton BR. Sustaining sexual and reproductive health through COVID-19 pandemic restrictions: qualitative interviews with Australian clinicians. *Sexual Health.* 2022;30:30.

138. Ngai FW, Chan PS. A Qualitative Evaluation of Telephone-Based Cognitive-Behavioral Therapy for Postpartum Mothers. *Clinical Nursing Research.* 2019;28(7):852-868.

139. Nguyen TC, Donovan EE, Wright ML. Doula Support Challenges and Coping Strategies during the COVID-19 Pandemic: Implications for Maternal Health Inequities. *Health Communication.* 2022;37(12):1496-1502.

140. Nicholson WK, Beckham AJ, Hatley K, et al. The Gestational Diabetes Management System (GooDMomS): development, feasibility and lessons learned from a patient-informed, web-based pregnancy and postpartum lifestyle intervention. *BMC Pregnancy & Childbirth.* 2016;16:277-277.

141. Nishi D, Imamura K, Watanabe K, et al. The preventive effect of internet-based cognitive behavioral therapy for prevention of depression during pregnancy and in the postpartum period (iPDP): a large scale randomized controlled trial. *Psychiatry and Clinical Neurosciences.* 2022.

142. O'Brien E, Rauf Z, Alfirevic Z, Lavender T. Women's experiences of outpatient induction of labour with remote continuous monitoring. *Midwifery.* 2013;29(4):325-331.

143. O'Neill L, Brennan L, Sheill G, Connolly D, Guinan E, Hussey J. Moving Forward With Telehealth in Cancer Rehabilitation: Patient Perspectives From a Mixed Methods Study. *JMIR Cancer.* 2023;9:e46077.

144. Özümerzifon Y, Ross A, Brinza T, Gibney G, Garber CE. Exploring a Dance/Movement Program on Mental Health and Well-Being in Survivors of Intimate Partner Violence During a Pandemic. *Frontiers in Psychiatry.* 2022;13.

145. Painter J, Turner J, Procter PM. Understanding and Accommodating Patient and Staff Choice When Implementing Video Consultations in Mental Health Services. *CIN: Computers, Informatics, Nursing.* 2021;39(10):578-583.

146. Penny RA, Bradford NK, Langbecker D. Registered nurse and midwife experiences of using videoconferencing in practice: A systematic review of qualitative studies. *Journal of Clinical Nursing.* 2018;27(5-6):e739-e752.

147. Pereira TAB, Santos IB, Mota RF, Fukusawa L, Azevedo-Santos IF, DeSantana JM. Beliefs and expectations of patients with fibromyalgia about telerehabilitation during Covid-19 pandemic: A qualitative study. *Musculoskelet Sci Pract.* 2023;67:102852.

148. Pierce LL, Steiner V, de Dios AM, Vollmer M, Govoni AL, Thompson TL. Qualitative analysis of a nurse's responses to stroke caregivers on a web-based supportive intervention. *Topics in Stroke Rehabilitation.* 2015;22(2):152-159.

149. Pierre-Wright MJ, Mathey L, Risser HJ. Can Telehealth Provide Timely and Equitable Quality Medical Forensic Services? Perspectives of Illinois Hospital Administrators. *J.* 2023;19(4):223-230.

150. Poon Z, Tan NC. A qualitative research study of primary care physicians' views of telehealth in delivering postnatal care to women. *BMC Primary Care.* 2022;23(1):206.

151. Power R, Ussher JM, Hawkey A, et al. Co-designed, culturally tailored cervical screening education with migrant and refugee women in Australia: a feasibility study. *BMC Women's Health.* 2022;22(1).

152. Raad T, Griffin A, George ES, et al. Experience and perceptions among rheumatoid arthritis patients following a telehealth-delivered dietary intervention: a qualitative study. *Rheumatol Int.* 2023;43(10):1913-1924.

153. Rasekaba TM, Lim K, Blackberry I, Gray K, Furler J. Telemedicine for Gestational Diabetes Mellitus (TeleGDM): A Mixed-Method Study Protocol of Effects of a Web-Based GDM Support System on Health Service Utilization, Maternal and Fetal Outcomes, Costs, and User Experience. *JMIR Research Protocols.* 2016;5(3):e163.

154. Rasekaba T, Nightingale H, Furler J, Lim WK, Triay J, Blackberry I. Women, clinician and IT staff perspectives on telehealth for enhanced gestational diabetes mellitus management in an Australian rural/regional setting. *Rural & Remote Health.* 2021;21(1):5983.

155. Rezel-Potts E, Free C, Syred J, Baraitser P. Expanding choice through online contraception: a theory of change to inform service development and evaluation. *BMJ Sexual & Reproductive Health.* 2020;46(2):108-115.

156. Rissel C, Khanal S, Raymond J, Clements V, Leung K, Nicholl M. Piloting a Telephone Based Health Coaching Program for Pregnant Women: A Mixed Methods Study. *Maternal & Child Health Journal.* 2019;23(3):307-315.

157. Rousseau A, Gaucher L, Gautier S, Mahrez I, Baumann S. How midwives implemented teleconsultations during the COVID-19 health crisis: a mixed-methods study. *BMJ Open.* 2022;12(4):e057292.

158. Saad M, Chan S, Nguyen L, Srivastava S, Appireddy R. Patient perceptions of the benefits and barriers of virtual postnatal care: a qualitative study. *BMC Pregnancy & Childbirth.* 2021;21(1):543.

159. Saraiya TC, Swarbrick M, Franklin L, Kass S, Campbell ANC, Hien DA. Perspectives on trauma and the design of a technology-based trauma-informed intervention for women receiving medications for addiction treatment in community-based settings. *Journal of Substance Abuse Treatment.* 2020;112:92-101.

160. Schmidt-Hantke J, Jacobi C. Investigating perspectives on e-health interventions to enhance maternal mental well-being: Results of a stakeholder interview. *PLOS Digit Health.* 2023;2(8):e0000326.

161. Schoebel V, Wayment C, Gaiser M, Page C, Buche J, Beck AJ. Telebehavioral Health During the COVID-19 Pandemic: A Qualitative Analysis of Provider Experiences and Perspectives. *Telemedicine journal and e-health : the official journal of the American Telemedicine Association.* 2021;27(8):947-954.

162. Sedlander E, Barboza KC, Jensen A, et al. Veterans' Preferences for Remote Management of Chronic Conditions. *Telemedicine Journal & E-Health.* 2018;24(3):229-235.

163. Seward MW, Simon D, Richardson M, Oken E, Gillman MW, Hivert M-F. Supporting healthful lifestyles during pregnancy: a health coach intervention pilot study. *BMC Pregnancy & Childbirth.* 2018;18(1):N.PAG-N.PAG.

164. Shah SP, Glenn GL, Hummel EM, et al. Caregiver tele-support group for Parkinson's disease: A pilot study. *Geriatric Nursing.* 2015;36(3):207-211.

165. Shaw H, Rohde P, Stice E. Participant feedback from peer-led, clinician-led, and internet-delivered eating disorder prevention interventions. *International Journal of Eating Disorders.* 2016;49(12):1087-1092.

166. Shroder M, Anders SH, Dorst M, Jackson GP. Communication Technology Use and Preferences for Pregnant Women and Their Caregivers. *AMIA Annual Symposium Proceedings/AMIA Symposium.* 2018;2018:1515-1523.

167. Simblett S, Matcham F, Siddi S, et al. Barriers to and Facilitators of Engagement With mHealth Technology for Remote Measurement and Management of Depression: Qualitative Analysis. *JMIR MHealth and UHealth.* 2019;7(1):e11325.

168. Simons A, Noordegraaf M, Van Regenmortel T. 'When it comes to relational trauma, you need people at the table': Therapist experiences of online therapy for families with a prior disclosure of sibling sexual abuse during Covid-19 pandemic lockdowns. *Journal of Family Therapy.* 2022;24:24.

169. Simony C, Clausen B, Beck M, et al. An invigorating journey towards better function and well-being: A qualitative study of knee osteoarthritis patients' experiences with an online exercise and education intervention. *Osteoarthr Cartil Open.* 2023;5(3):100384.

170. Singla DR, Lemberg-Pelly S, Lawson A, Zahedi N, Thomas-Jacques T, Dennis CL. Implementing Psychological Interventions Through Nonspecialist Providers and Telemedicine in High-Income Countries: Qualitative Study from a Multistakeholder Perspective. *JMIR Mental Health.* 2020;7(8):e19271.

171. Skolasky RL, Kimball ER, Galyean P, et al. Identifying Perceptions, Experiences, and Recommendations of Telehealth Physical Therapy for Patients With Chronic Low Back Pain: A Mixed Methods Survey. *Archives of Physical Medicine & Rehabilitation.* 2022;103(10):1935-1943.

172. Slightam C, Wray C, Tisdale RL, Zulman DM, Gray C. Opportunities to Enhance the Implementation of Veterans Affairs Video-Based Care: Qualitative Perspectives of Providers from Diverse Specialties. *J Med Internet Res.* 2023;25:e43314.

173. Smith D, Johnston K, Carlisle K, et al. Client perceptions of the BreastScreen Australia remote radiology assessment model. *BMC Women's Health.* 2021;21(1).

174. Snaith VJ, Robson SC, Hewison J. Antenatal telephone support intervention and uterine artery Doppler screening: A qualitative exploration of women's views. *Midwifery.* 2015;31(5):512-518.

175. Spiby H, Faucher MA, Sands G, Roberts J, Kennedy HP. A qualitative study of midwives' perceptions on using video-calling in early labor. *Birth.* 2019;46(1):105-112.

176. Spiess ST, Gardner E, Turner C, et al. We Cannot Put This Genie Back in the Bottle: Qualitative Interview Study Among Family Medicine Providers About Their Experiences With Virtual Visits During the COVID-19 Pandemic. *J Med Internet Res.* 2023;25:e43877.

177. Sun CA, Shenk Z, Renda S, et al. Experiences and Perceptions of Telehealth Visits in Diabetes Care During and After the COVID-19 Pandemic Among Adults With Type 2 Diabetes and Their Providers: Qualitative Study. *JMIR Diabetes.* 2023;8:e44283.

178. Sundstrom B, DeMaria AL, Ferrara M, Meier S, Billings D. "The Closer, the Better:" The Role of Telehealth in Increasing Contraceptive Access Among Women in Rural South Carolina. *Maternal & Child Health Journal.* 2019;23(9):1196-1205.

179. Sundstrom B, DeMaria AL, Ferrara M, Smith E, McInnis S. "People are struggling in this area:" a qualitative study of women's perspectives of telehealth in rural South Carolina. *Women & Health.* 2020;60(3):352-365.

180. Talal AH, Sofikitou EM, Wang K, Dickerson S, Jaanimagi U, Markatou M. High Satisfaction with Patient-Centered Telemedicine for Hepatitis C Virus Delivered to Substance Users: A Mixed-Methods Study. *Telemedicine Journal & E Health.* 2022;04:04.

181. Talal AH, Sofikitou EM, Wang K, Dickerson S, Jaanimagi U, Markatou M. High Satisfaction with Patient-Centered Telemedicine for Hepatitis C Virus Delivered to Substance Users: A Mixed-Methods Study. *Telemed J E Health.* 2023;29(3):395-407.

182. Talati AN, Mallampati D, Johnson JD, West-Honart A, Vladutiu C, Menard MK. 970 Future telehealth use for maternity care beyond COVID19: mixed-methods survey of a regional perinatal health-center. *American Journal of Obstetrics & Gynecology.* 2021;224(2):S601-S602.

183. Talati AN, Mallampati D, Johnson JD, West-Honart A, Vladutiu C, Menard MK. 1000 Provider satisfaction with telehealth for maternity-care during COVID-19: mixed-methods survey of a regional perinatal health-center. *American Journal of Obstetrics & Gynecology.* 2021;224(2):S619-S620.

184. Tavener CR, Kyriacou C, Elmascri I, Cruickshank A, Das S. Rapid introduction of virtual consultation in a hospital-based Consultant-led Antenatal Clinic to minimise exposure of pregnant women to COVID-19. *BMJ Open Quality.* 2022;11(1):01.

185. Taylor M, Kikkawa N, Hoehn E, et al. The importance of external clinical facilitation for a perinatal and infant telemental health service. *Journal of Telemedicine & Telecare.* 2019;25(9):566-571.

186. Thawani SP, Minen MT, Stainman RS, et al. Neurologists' Evaluations of Experience and Effectiveness of Teleneurology Encounters. *Telemedicine Journal & E Health.* 2022;14:14.

187. Therouanne P, Hayotte M, Halgand F, d'Arripe-Longueville F. The Acceptability of Technology-Based Physical Activity Interventions in Postbariatric Surgery Women: Insights From Qualitative Analysis Using the Unified Theory of Acceptance and Use of Technology 2 Model. *JMIR Hum Factors.* 2023;10:e42178.

188. Thiel de Bocanegra H, Goliaei Z, Khan N, Banna S, Behnam R, Mody SK. Refugee Women's Receptiveness for Virtual Engagement on Reproductive Health During the COVID-19 Pandemic. *International Journal of Behavioral Medicine.* 2022;12:12.

189. Truong LK, Mosewich AD, Miciak M, et al. "I feel I'm leading the charge." Experiences of a virtual physiotherapist-guided knee health program for persons at-risk of osteoarthritis after a sport-related knee injury. *Osteoarthr Cartil Open.* 2023;5(1):100333.

190. Tutty E, Petelin L, McKinley J, et al. Evaluation of telephone genetic counselling to facilitate germline BRCA1/2 testing in women with high-grade serous ovarian cancer. *European Journal of Human Genetics.* 2019;27(8):1186-1196.

191. Uscher-Pines L, Demirci J, Waymouth M, et al. Impact of telelactation services on breastfeeding outcomes among Black and Latinx parents: protocol for the Tele-MILC randomized controlled trial. *Trials [Electronic Resource].* 2022;23(1):5.

192. Uskup DK, Nieto O, Rosenberg-Carlson E, Lee SJ, Milburn NG, Brooks RA. Acceptability and Appropriateness of Digital PrEP Interventions for Black and Latina Cisgender Women: Perspectives From Service Providers in Los Angeles County. *Journal of Acquired Immune Deficiency Syndromes: JAIDS.* 2022;90(S1):S134-S140.

193. Van Erkel FM, Pet MJ, Bossink EHM, et al. Experiences of patients and health care professionals on the quality of telephone follow-up care during the COVID-19 pandemic: A large qualitative study in a multidisciplinary academic setting. *BMJ Open.* 2022;12(3).

194. Van Gerwen OT, Austin EL, Bethune CW, Sullivan PS, Muzny CA. Sexual healthcare and at-home STI test collection: attitudes and preferences of transgender women in the Southeastern United States. *Front.* 2023;11:1187206.

195. van Ierssel J, O'Neil J, King J, Zemek R, Sveistrup H. Clinician Perspectives on Providing Concussion Assessment and Management via Telehealth: A Mixed-Methods Study. *J Head Trauma Rehabil.* 2023;38(3):E233-E243.

196. von Below C, Bergsten J, Midbris T, Philips B, Werbart A. It turned into something else: patients' long-term experiences of transitions to or from telepsychotherapy during the COVID-19 pandemic. *Front Psychol.* 2023;14:1142233.

197. Walker AJ, Lewis FM, Al-Mulla H, Alzawad Z, Chi N-C. Being Fully Present: Gains Patients Attribute to a Telephone-Delivered Parenting Program for Child-Rearing Mothers With Cancer. *Cancer Nursing.* 2018;41(4):E12-E17.

198. White V, Bastable A, Solo I, et al. Telehealth cancer care consultations during the COVID-19 pandemic: a qualitative study of the experiences of Australians affected by cancer. *Supportive Care in Cancer.* 2022;30(8):6659-6668.

199. Wisner KL, Sit DKY, McShea M, et al. Telephone-based depression care management for postpartum women: A randomized controlled trial. *Journal of Clinical Psychiatry.* 2017;78(9):1369-1375.

200. Wit RF, Lucassen DA, Beulen YH, et al. Midwives' Experiences with and Perspectives on Online (Nutritional) Counselling and mHealth Applications for Pregnant Women; an Explorative Qualitative Study. *International Journal of Environmental Research & Public Health [Electronic Resource].* 2021;18(13):23.

201. Woods J, Elmore SN, Glenn L, Maues J, James D, Roberson ML. A Qualitative Study of the Impact of the COVID-19 Pandemic on Metastatic Breast Cancer Care. *J Patient Exp.* 2023;10:23743735231167973.

202. Wyatt SN, Rhoads SJ, Green AL, Ott RE, Sandlin AT, Magann EF. Maternal response to high-risk obstetric telemedicine consults when perinatal prognosis is poor. *Australian & New Zealand Journal of Obstetrics & Gynaecology.* 2013;53(5):494-497.

203. Yelverton V, Gass SJ, Amoatika D, et al. The Future of Telehealth in Human Immunodeficiency Virus Care: A Qualitative Study of Patient and Provider Perspectives in South Carolina. *AIDS Patient Care STDS.* 2023;37(10):459-468.

204. Zaccari B, Loftis JM, Haywood T, Hubbard K, Clark J, Kelly UA. Synchronous Telehealth Yoga and Cognitive Processing Group Therapies for Women Veterans with Posttraumatic Stress Disorder: A Multisite Randomized Controlled Trial Adapted for COVID-19. *Telemedicine Journal & E Health.* 2022;29:29.

205. Zilliacus E, Meiser B, Lobb E, Dudding TE, Barlow-Stewart K, Tucker K. The virtual consultation: practitioners' experiences of genetic counseling by videoconferencing in Australia. *Telemedicine Journal & E-Health.* 2010;16(3):350-357.
