# Supplementary material for: Experiences Receiving and Delivering Virtual Health Care For Women: Qualitative Evidence Synthesis
Source: J Med Internet Res. 2025 May 15;27:e68314. doi: 10.2196/68314 (PMC12123244; doi:10.2196/68314)
Supplement: Multimedia Appendix 5 [file jmir_v27i1e68314_app5.docx]

| **Study details and characteristics** | **Condition focus and aim of study** | **Enrollment criteria** | **Participant characteristics** | **Telehealth details** | **Qualitative data collection, analysis, and theoretical frameworks** | **Conclusions** |
| --- | --- | --- | --- | --- | --- | --- |
| **First author, year:** Buse (2022)  **Geographic location:** Urban North Carolina  **Clinical setting:** Other: Oncology  **Methods type:** Embedded mixed methods  **Total enrolled patients/providers:** 15 patients | **Focus:** Early Breast Cancer  **Aim:** To provide a qualitative description of the perspectives of women age ≥65 who received both in-person and telemedicine care for early-stage breast cancer. | **Inclusion**: Patients with a telemedicine appt between Jan - Sept 2021  **Exclusion**: Patients with no memory of appt, cognitive disability, hearing loss. | **Demographics:**  *Age range:* 67-85 years  *Mean age:* 73 ± 5.24 years  *Education:*  High school/some college: 4  College: 4  Advanced degree: 5  *Previous telehealth:* NR  *Underrepresented groups:* No | **Type:** Telephone and Video  **Scheduled:** Yes  **Training:** NR | **Collection method:** Interviews  **Analysis method:** Codebook made from interview guide, iterative expansion of sub-themes based on transcripts.  **Frameworks:** NR | "Patient-provider discussions about appointment modality should take into account newness of diagnosis, patient familiarity with the care team, travel burden, and necessity of physical exam." |
| **First author, year:** Allison, 2023  **Geographic location:** Midwest, USA  **Clinical setting:** Other: Specialty  **Methods type:** Qualitative  **Total enrolled patients/providers:** 15 Providers | **Focus:** Sexual Assault Care  **Aim:** To assess ED nurses' perceptions of influences on telemedicine use, the utility and feasibility of teleSANE, and identify potential influences on teleSANE implementation in EDs. | **Inclusion:** Providers currently employed in an Arkansas ED, who were not SANE trained and had completed a sexual assault forensic medical examination within the last year. | **Demographics:**  *Mean time in nursing practice:* 15.7 (range 3-35) years  *Mean time with current ED:* 9.7 (range 1-20 years)  *Mean time in current role:* 9 (range 2-18 years)  *Underrepresented groups:* Rural focus | **Type:** Video  **Scheduled:** Yes  **Training:** NR | **Collection method:** Interviews  **Analysis method:** Deductive and inductive coding  **Frameworks:** CFIR | "Findings highlight the unique needs of sexual assault survivors receiving telemedicine services in EDs, particularly those in rural communities with heightened privacy concerns and limited access to specialty care." |
| **First author, year:** Davenport, 2023  **Geographic location:** United States  **Clinical setting:** OB/GYN Surgery  **Methods type:** Embedded mixed methods  **Total enrolled patients/providers:** 20 patients | **Focus:** Urogynecology surgery preoperative visits  **Aim:** To explore and compare patient experience, decision making, and satisfaction among women undergoing telemedicine or in-person preoperative visits. | **Inclusion:** Eligible patients were ≥ 6 weeks remote from their surgery and had already undergone a preoperative visit and physical examination. Type of preoperative appt (telemedicine vs in-person) for potential participants was agreed upon via shared decision making between the participant and surgeon. | **Demographics:**  *Age range:* 19-60 years  *Mean age:* 43.4 years  *Education:* NR  *Underrepresented groups:* No  *Previous telehealth:* NR | **Type:** Video  **Scheduled:** Yes  **Training:** NR | **Collection method:** Interviews  **Analysis method:** Iterative review and constant comparative method  **Frameworks:** NR | "Decision making for selecting an in-person or telemedicine visit is complex and involves balancing multiple advantages and disadvantages. Participant experience was similar for both visit types with high satisfaction." |
| **First author, year:** Howard, 2023  **Geographic location:** Washington  **Clinical setting:** Mental Health  **Methods type:** Qualitative  **Total enrolled patients/providers:** 8 patients, 5 providers | **Focus:** Perinatal mental health disorders  **Aim:** To identify facilitators and barriers to receiving perinatal telepsychiatry care from the perspective of patients, clinic staff and psychiatrists. | **Inclusion (patients):** English-speaking and had been scheduled for an appt (including those who attended and did not attend their appt) with the perinatal telepsychiatry clinic between 14 May and 1 August 2021.  **Inclusion (providers)**: Clinic staff | **Demographics (patients):**  *Age:* NR  *Education:* NR  *Underrepresented groups:* No  *Previous telehealth:* NR  **Demographics (providers):**  *Provider role:*  Psychiatrists: 2  Mental Health Navigator: 1  Navigator supervisor: 1  Telepsychiatry Expert: 1 | **Type:** Video  **Scheduled:** Yes  **Training:** NR | **Collection method:** Interviews  **Analysis methods:** Blinded qualitative and thematic analysis  **Frameworks:** Patient Journey Conceptual Model | "The findings from this study suggest that telepsychiatry services are perceived positively by patients and care staff and have the potential to improve access to mental healthcare for perinatal patients." |
| **First author, year:** Howell, 2023  **Geographic location:** Virginia  **Clinical setting:** OB/GYN  **Methods type:** Embedded mixed methods  **Total enrolled patients/providers:** 20 patients | **Focus:** BF  **Aim:** To explore experiences with telelactation among Black parents and identify strategies for making services more culturally appropriate. | **Inclusion:** Trial participants who self-identified as Black, received access to telelactation services through the trial (intervention arm only), and were between 8 and 12 weeks postpartum (to allow participants sufficient time to gain experience with telelactation services and to minimize recall bias). | **Demographics:**  *Age:* NR  *Education:* NR  *Underrepresented groups:* Racial/ethnic marginalized group focus  *Previous telehealth:* NR | **Type:** Video  **Scheduled:** No  **Training:** NR | **Collection method:** Interviews  **Analysis methods:** Iteratively developed and refined a codebook.  **Frameworks:** Sekhon et al’s theoretical framework of acceptability, Equity-centered model developed by the RAND Corporation’s Center for Advancing Racial Equity Policy. | "While Black parents in our sample found telelactation services to be acceptable, telelactation could not, in isolation, address the myriad barriers to long-duration BF. Several changes could be made to telelactation services to increase their use by minoritized populations." |
| **First author, year:** Kissler, 2023  **Geographical location:** Colorado  **Clinical setting:** OB/GYN  **Methods type:** Qualitative  **Total enrolled patients/providers:** 14 patients, 17 providers | **Focus:** Perinatal Health  **Aim:** To describe the experiences of perinatal patients and providers with telehealth during and after the acute phase of the COVID-19 pandemic to inform future utilization of telehealth for the delivery of high-quality, accessible, and equitable perinatal care to diverse communities. | **Inclusion:** Eligible participants from a parent study evaluating telehealth implementation in Colorado nurse-led care models during the COVID-19 pandemic, who indicated a willingness to be contacted for interviews and were engaged in perinatal telehealth care. | **Demographics (patients)**:  *Age:* NR  *Education:* NR  *Underrepresented groups:* Racial/ethnic marginalized group focus, rural rocus  *Previous telehealth:* NR  **Demographics (providers):** NR | **Type:** Video  **Scheduled:** Yes  **Training:** NR | **Collection method:** Interviews  **Analysis methods:** Team-based, iterative, inductive, and deductive analytic strategies, including field notes, memoing, general theme analysis, consultative/ reflexive analysis, and triangulation with experts in perinatal care.  **Frameworks:** Practical robust implementation and sustainability model extension of the Reach, Efficacy, Adoption, Implementation, and Maintenance framework. | "This study provides insight into priorities for continued telehealth utilization focused on providing equitable access to perinatal care. Rather than returning to practices from before the COVID-19 pandemic formed from longstanding routines and perceived limitations, providers are encouraged to capitalize on the rapid innovations in telehealth to build a more effective, equitable, and patient-centered approach to perinatal care." |
| **First author, year:** Kozica-Olenski, 2023  **Geographical location:** Victoria, Australia  **Clinical setting:** OB/GYN  **Methods type:** Embedded Mixed Methods  **Total enrolled patients/providers:** 18 patients, 6 providers | **Focus:** Menopause  **Aim:** To explore women’s and clinician’s experiences and acceptability of telehealth use within a specialized multidisciplinary menopause service during the COVID-19 pandemic. | **Inclusion (patients):** Women who attended the Monash Health menopause service were invited to complete an anonymous online survey and register their interest in interview participation. Those interested were contacted and provided written study information. Participants were required to speak English.  **Inclusion (providers):** A purposeful sample of endocrinology and gynecology specialists (trainees/ consultants) who provided menopause care at Monash Health. | **Demographics (patients)**:  *Mean age:* 52.39 ± 10.78 years  *Age group (years):*  <45: 2  >45-60: 12  >60: 4  *Education:* NR  *Underrepresented groups:* No  *Previous telehealth:* NR  **Demographics (providers):**  *Role:*  Endocrinologist consultants: 2  Endocrinologist fellows/trainees: 2  Gynecologist consultants: 2  *Time in role (years):*  >10: 3  5: 1  <1: 2 | **Type:** Video  **Scheduled:** Yes  **Training:** NR | **Collection method:** Interviews  **Analysis methods:** Thematic analysis  **Frameworks:** NASSS framework | "Telehealth was viewed as acceptable, supporting the ongoing delivery of a hybrid service model of telehealth and face-to-face menopause care. The findings provide valuable information to improve the menopause service to meet the needs of women during the ongoing current pandemic and beyond." |
| **First author, year:** Nguyen-Grozavu, 2023  **Geographical location:** California  **Clinical setting:** OB/GYN Oncology  **Methods type:** Embedded Mixed Methods  **Total enrolled patients/providers:** 27 patients | **Focus:** Breast cancer  **Aim:** To build upon existing scientific literature by examining the potential benefits and challenges, and highlight proposed strategies to address these challenges, thereby improving the healthcare experience with the increasing use of telehealth. | **Inclusion:** Participants who self-identified as a Hispanic female, were ≥18 years old, received a breast cancer diagnosis, and displayed cognitive competence. | **Demographics:**  *Mean age:* 54.4 ± 11.6 years  *Education:*  Less than high school: 5  High school/GED: 12  Some College: 6  College Graduate: 3  Graduate Degree: 1  *Underrepresented groups:* Racial/ethnic marginalized group focus  *Previous telehealth:* NR | **Type:** Video  **Scheduled:** Yes  **Training:** NR | **Collection method:** Interviews  **Analysis method:** Reflexive thematic analysis  **Frameworks:** NR | "The COVID-19 pandemic has greatly affected the landscape of how care is provided, with a greater shift to telehealth services. More research is needed to further examine the challenges of telehealth, particularly for groups that are disproportionately affected, to avoid the disruption of patients’ cancer care and to promote a better patient healthcare experience." |
| **First author, year:** Simon, 2023  **Geographical location:** United Kingdom  **Clinical setting:** Mental health  **Methods type:** Qualitative  **Total enrolled patients/providers:** 7 providers | **Focus:** Perinatal mood disorders  **Aim:** To examine facilitator perspectives of a videoconference-delivered group Acceptance Commitment Therapy (ACT) intervention for perinatal women with moderate- severe mood and/or anxiety disorders. | **Inclusion:** NHS staff working in a specialist PCMHS who were co-facilitating videoconference-delivered ACT-PNMH. | **Demographics:**  *Sex:*  Males: 2  Females: 5  *Education*:  Degree level or above: 7  *Race/ethnicity:*  White British: 7 | **Type:** Video  **Scheduled:** Yes  **Training:** NR | **Collection method:** Interviews  **Analysis method:** Thematic analysis  **Frameworks:** NR | "This study raises important considerations regarding the use of videoconference-delivered group ACT in the perinatal context. There are opportunities afforded by videoconference- delivered group therapies, which is important and timely given the increased drive towards improving access to perinatal services and psychological therapies, and the need for “COVID-proof” therapies. Recommendations for best practice are offered." |
| **First author, year:** Sterba, 2023  **Geographical location:** South Carolina  **Clinical setting:** Mental Health  **Methods type:** Embedded mixed methods  **Total enrolled patients/providers:** 22 providers | **Focus:** Perinatal mood & substance disorders  **Aim:** To characterize barriers and facilitators to implementing a telemedicine program for mental health and substance use disorder in community obstetric and pediatric clinics. | **Inclusion:** Staff and providers at pediatric and obstetric practices engaged in the Women’s Reproductive Behavioral Health Telemedicine program with the Medical University of South Carolina and the telemedicine providers involved in care delivery. | **Demographics:**  *Clinic/team type:*  Pediatric clinics: 4  Obstetric clinics: 14  Women’s reproductive behavioral health team: 4 (2 reproductive psychiatrists, 1 telemedicine medical director, 1 clinical program coordinator) | **Type:** Telephone and video  **Scheduled:** Yes  **Training:** NR | **Collection method:** Focus groups and an Interview  **Analysis method:** Combined inductive-deductive template analysis approach  **Frameworks:** EPIS | "Capitalizing on clinics’ commitment to care for women’s needs and a high demand for mental health and substance use disorder services while also addressing resource and technology needs will facilitate telemedicine program success. Study results may have implications for potential marketing, onboarding and monitoring implementation strategies to support clinics engaging in telemedicine programs." |
| **First author, year:** Zilliacus, 2010  **Geographical location:** New South Wales, Australia  **Clinical setting:** Other: Familial Cancer Service  **Methods types:** Qualitative  **Total enrolled patients/providers:** 12 patients | **Focus:** Breast and/or ovarian cancer  **Aim:** To explore women's experiences using telemedicine and satisfaction with providers and technology during telegenetics. | **Inclusion:** Participants had received genetic counseling for hereditary breast and/or ovarian cancer via telemedicine within the last 12 months, had a high-risk for breast and/or ovarian cancer, were English speaking, and could give informed consent. | **Demographics:**  *Age group (years):*  36-45 years: 2  46-55 years: 3  56-65 years: 6  >65 years: 1  *Education:*  High school: 6  Trade: 3  Diploma: 3  Bachelor: 0  Postgraduate: 0  *Previous telehealth experience:* Yes  *Underrepresented group:* No | **Type:** Video  **Scheduled:** Yes  **Training:** NR | **Collection method:** Interview  **Analysis method:** Data categorization and comparisons according to profession group and other participants. Themes were identified.  **Frameworks:** Miles and Huberman conceptual framework to emphasize the potential for researcher bias and the explicitness of potential strategies to guard against bias and increase finding validity. | "Patients attending for HBOC genetic counseling are generally highly satisfied with the technology and the interaction. Care should be taken, however, with patients with more complex psychosocial needs." |
| **First author, year:** Williamson, 2018  **Geographic location:** United Kingdom  **Clinical setting:** OB/GYN, Gyn Oncology  **Methods type:** Qualitative  **Total enrolled patients/providers:** 25 patients, 8 providers | **Focus:** Endometrial cancer  **Aim:** To explore patient and clinical nurse specialist experiences of telephone follow-up after patients received treatment for endometrial cancer. | **Inclusion (patients):** Randomly selected from the 129 participants from the TFU group in the ENDCAT trial  **Inclusion (providers)***:* NR | **Demographics (patients)***:*  *Age range:* 52-82 years  *Education:*  No formal qualifications: 5  O/A level: 16  Vocational: 5  Diploma/degree: 8  *Previous telehealth:* NR  *Underrepresented groups:* No  **Demographics (providers)**: All oncology clinical nurse specialists | **Type:** Telephone  **Scheduled:** NR  **Training:** NR | **Collection method:** Interviews  **Analysis method:** Content analysis  **Frameworks:** NR | “Alternative models of care such as TFU provided by CNSs provides the care, information and support to enable women treated for endometrial cancer to self-manage and live well.” |
| **First author, year:** Vincze, 2018  **Geographic location:** Australia  **Clinical setting:** OB/GYN  **Methods type:** Explanatory mixed methods  **Total enrolled patients/providers:** 21 patients | **Focus:** Postpartum weight management  **Aim:** To provide qualitative insight into women’s experiences of participating in VITAL change for mums, specifically for engaging with the dietitian and exercise physiologist through video consultations for tailored nutrition and exercise care. | **Inclusion:** Women who were 3-12 months postpartum with a BMI of ≥25 kg/m^2^ or a >2 kg weight retention from their most recent childbirth, and had participated in the 8-week single-arm pre-post VITAL feasibility study. | **Demographics:**  *Mean age:* 32.3 ± 3.0 years  *Education:*  Certificate/diploma: 4  University degree or higher: 17  *Underrepresented groups:* No | **Type:** Video  **Scheduled:** Yes  **Training:** NR | **Collection method:** Interviews  **Analysis method:** Thematic data analysis  **Frameworks:** NR | “Themes relating to the video consultation experience included feeling that they did not differ from other consultations, they were convenient, and the length of time and flexible options were appropriate; however, there was a desire for increased contact frequency. The dietitian and exercise physiologist were perceived to increase the participants’ knowledge and confidence to improve health behaviors. The approach to setting realistic and tailored goals was well received. Tailored advice from a dietitian and exercise physiologist received via video consultations is acceptable for postpartum women and offers a viable alternative to in-person care.” |
| **First author, year:** Tan, 2013  **Geographic location:** Texas  **Clinical setting:** Mental health  **Methods type:** Explanatory mixed methods  **Total enrolled patients/providers:**34 patients | **Focus:** Depression, Chronic pain  **Aim:** Evaluate the feasibility and effects of a novel clinical intervention that combined group therapy and biofeedback training on women veterans in rural areas. | **Inclusion:** Women veterans residing in rural areas served by the CBOCs in Conroe and Lufkin, Texas, with a chronic pain condition; and either PTSD, depression, or both. | **Demographics:**  *Age range:* 22-67 years  *Mean age:* 49.5 years  *Education:* NR  *Underrepresented groups:* Rural Focus  *Previous telehealth experience:* Yes | **Type:** Video, tele-conference to larger VA medical center from their CBOC  **Scheduled:** Yes  **Training:** NR | **Collection method:** Focus groups  **Analysis method:** NR  **Frameworks:** NR | “It is feasible to provide treatment to women veterans living in rural areas by utilizing video-teleconferencing technology between larger VA medical centers and facilities at CBOCs in more rural settings. A controlled trial of the intervention is warranted.” |
| **First author, year:** Srinivasulu, 2022  **Geographic location:** New York, NY  **Clinical setting:** Primary Care  **Methods type:** Qualitative  **Total enrolled patients/providers:**30 patients | **Focus:** Sexual and reproductive healthcare  **Aim:** To explore the preferences of women seeking sexual and reproductive healthcare via telehealth in primary care. | **Inclusion:** English speaking patients, aged 18-45 years, residing in NY, who had experienced a consultation with PCP. | **Demographics:**  *Age:* NR  *Education:* NR  *Race/ethnicity:*  White: 40%  Black/African American: 30%  *Underrepresented groups:* No | **Type:** Telephone and Video  **Scheduled:** NR  **Training:** NR | **Collection method:** Focus groups and interviews  **Analysis method:** Reflexive thematic analysis  **Frameworks:** NR | “Participants find telehealth SRH in primary care preferable, underscoring the importance of offering and expanding this care. As telehealth SRH expands, providers should strengthen quality by building rapport to facilitate conversations on ‘serious’ topics and their ability to help patients remotely.” |
| **First author, year:** Spiby, 2014  **Geographic location:** Midlands of England  **Clinical setting:** OB/GYN  **Methods type:** Qualitative  **Total enrolled patients/providers:**23 providers | **Focus:** Early labor  **Aim:** To explore midwives' concerns, experiences, and perceptions of the characteristics of satisfactory and unsatisfactory telephone contact and conversations with women in early labor. | **Inclusion:** Midwives, labor suite coordinators, midwifery risk manager, labor suite managers, modern matrons, obstetricians, midwifery lecturer, and labor ward receptionist from a NHS Trust in England where maternity care is provided on two sites (site 1 with 4000 births/year and site 2 with 5000 births/year with mixed risk caseloads). | **Demographics:**  *Role:*  Labor suite coordinators: 8 (4 from each hospital)  Labor ward midwives: 6 (mixed clinical experience)  Midwifery risk manager: 1  Labor suite managers: 2  Modern matrons: 2 Obstetricians: 2  Midwifery lecturer: 1 Labor ward receptionist: 1  *Underrepresented Groups:* No | **Type:** Telephone  **Scheduled:** Yes  **Training:** NR | **Collection method:** Focus groups  **Analysis method:** Interpretative phenomenological analysis  **Frameworks:** NR | “Because midwives are trying to reconcile the two conflicting priorities of responding to women's needs and protecting the labor ward from inappropriate admissions, the potential always exists for women's needs to be ‘not heard’ or marginalized. Implications for practice: the primary recommendation is that early labor telephone triage should be a discrete service, staffed by midwives who have been trained for this service, working independently of labor ward workloads.” |
| **First author, year:** Song, 2022  **Geographic location:** Illinois  **Clinical setting:** Primary care  **Methods type:** Qualitative  **Total enrolled patients/providers:**40 providers | **Focus:** Contraception  **Aim:** To understand provider perspectives on the effect of telemedicine implementation on patient care, exploring the benefits and barriers to making telemedicine provision of contraceptive services a satisfactory and acceptable form of service delivery. | **Inclusion:** Clinicians and clinical support staff who provided telemedicine services, and administration/billing staff with experience supporting telemedicine operations. Participants were required to respond yes to two screener questions to determine if they had provided/helped provide contraceptive services during the pandemic and provided/helped provide care via some telemedicine modality during the pandemic. | **Demographics:**  *Specialty:*  OB-GYN (physicians, midwives): 8  Family medicine/ pediatrician (physician, nurse practitioners, physician assistants) 12  Additional roles -  clinic leadership: 3 | **Type:** Telephone and video  **Scheduled:** Yes  **Training:** NR | **Collection method:** Interviews  **Analysis method:** Thematic analysis  **Frameworks:** Patient Centered Contraceptive Care framework | “Providers felt telemedicine provision of contraception positively impacted patient care. Improvements to counseling and easier access to method switching suggest that telemedicine implementation may help reduce contraceptive coercion. Our findings highlight the need to integrate LARC care with telemedicine workflows, improve patient privacy protections, and promote equitable access to all telemedicine modalities.” |
| **First author, year:** Singla, 2022  **Geographic location:** Toronto Canada, UNC Chapel Hill, Northshore Chicago, Illinois  **Clinical setting:** Mental Health  **Methods type:** Triangulation mixed methods  **Total enrolled patients/providers:**23 patients, 28 providers | **Focus:** Perinatal depression and anxiety  **Aim:** To present a BA model as a possible solution to mental healthcare challenges and disparities during the COVID-19 pandemic for perinatal women with depression/ anxiety, and to the movements against racial injustice. In the SUMMIT trial, patient and provider perspectives were identified on the barriers and facilitators to receiving and delivering BA. Present case studies of BA adapted during COVID-19 and the racial justice movement to address disparities in evidence-based psychological intervention access. | **Inclusion (patients):** English or Spanish speaking women (aged ≥18 years) with depressive symptoms (EPDS ≥10), who were up to 36 weeks pregnant or 4-30 weeks postpartum.  **Exclusion (patients)**: Active suicidal intent, active substance abuse or dependence psychosis or mania, a recent medication change, ongoing psychotherapy or severe fetal anomalies, stillbirth, or infant death at the time of enrollment.  **Inclusion (providers):** NR | **Demographics (patients):**  *Age range:* 20-40 years  *Mean age:* 32 years  *Education:* NR  *Underrepresented groups:* No  **Demographics (providers):**  *Role:*  Non-specialist providers (nurses and midwives without formal experience delivering mental healthcare): 15  Specialist providers (social workers, psychologists, psychiatrists): 13 | **Type:** Video  **Scheduled:** Yes  **Training:** Weekly treatment sessions delivered to an individual participant by a trained provider using a secure, telehealth server such as Zoom or WebEx. | **Collection method:** Interviews  **Analysis method:** Content analysis  **Frameworks:** NR | “BA offers a person-centered model to facilitate social connection through creative problem-solving for women with perinatal depressive and anxiety symptoms within the context of the COVID-19 pandemic. Explicit discussion of race and racial injustice during sessions is an important and helpful aspect in psychological treatments.” |
| **First author, year:** Silverio, 2021  **Geographic location:** South London, UK  **Clinical setting:** OB/GYN  **Methods type:** Qualitative  **Total enrolled patients/providers:**23 patients | **Focus:** Pregnancy and childbirth  **Aim:** To understand the experiences of pregnant women with changes in perinatal care and childbirth during the COVID-19 pandemic, with an increased volume of virtual visits. | **Inclusion:** Women who gave birth in South London during between March and August 2020, and had received some of their maternity care prior to both the COVID-19 pandemic and the UK 'lockdown' restrictions (23 March to 13 May 2020). | **Demographics:**  *Age range:* 27-44 years  *Mean age:* 35 years  *Education:* NR  *Underrepresented groups:* No | **Type:** Video  **Scheduled:** Yes  **Training:** NR | **Collection method:** Interviews  **Analysis method:** Template analysis and iterative analysis  **Frameworks:** NR | “Women reported mixed views on the reduction in scheduled in-person appointments. The increase in remote care, especially via telephone, was not well endorsed by women. Furthermore, women reported an under reliance on health care professionals for support, rather turning to family.” |
| **First author, year:** Sessanna, 2021  **Geographic location:** United States  **Clinical setting:** Mental Health  **Methods type:** Qualitative  **Total enrolled patients/providers:**14 patients | **Focus:** Multiple sclerosis  **Aim:** To establish acceptability of an online course for adults living with MS. | **Inclusion:** Physician diagnosed MS, aged 30-70 years, low-moderate disability, medically stable, community-dwelling, understand/ read/ speak English.  **Exclusion:** Unstable medical problem, condition likely to interfere with the intervention or outcome assessment, currently receiving another form of psychological intervention. | **Demographics:** NR  *Underrepresented groups:* No | **Type:** Video  **Scheduled:** Yes  **Training:** NR | **Collection method:** Focus groups  **Analysis method:** Reflexive thematic analysis  **Frameworks:** An experiential framework | “Time and length for both courses was found acceptable, camaraderie and interconnectedness were essential, having choice regarding course delivery format was important, and being provided with organized learning materials at the course start in a binder or packet was considered imperative. Acceptability was established for both the online and onsite formats.” |
| **First author, year:** Reynolds-Wright, 2022  **Geographic location:** Edinburgh, Scotland  **Clinical setting:** OB/GYN  **Methods type:** Qualitative  **Total enrolled patients/providers:**16 providers | **Focus:** Abortion  **Aim:** To explore views and experiences of ACPs involved in providing telemedicine medical abortion during COVID-19 to inform future telemedicine abortion practice. | **Inclusion:** Abortion Care Provider from the NHS Lothian telemedicine abortion service. | **Demographics:**  *Professions:* MD, RN, Midwives | **Type:** Telephone  **Scheduled:** Yes  **Training:** NR | **Collection method:** Interviews  **Analysis method:** Thematic analysis  **Frameworks:** NR | “Abortion Care Providers providing telemedicine abortion care value this option for patients and believe it should remain beyond the COVID-19 pandemic. Safeguarding patients and the selective use of ultrasound can be initially challenging; however, with experience, staff confidence improves.” |
| **First author, year:** Pipkin, 2022  **Geographic location:** England  **Clinical setting:** Gender Services  **Methods type:** Qualitative  **Total enrolled patients/providers:**6 patients | **Focus:**  Psychological distress due to gender identity and transition  **Aim:** To understand how people experience a compassion-focused therapy group intervention online. | **Inclusion:** Transgender and gender non-conforming individuals accessing medical and psychosocial support at the Gender Service. | **Demographics:**  *Age range:* 30-54 years  *Education:* NR  *Underrepresented groups:* Gender/ sexuality marginalized focus | **Type:** Video  **Scheduled:** Yes  **Training:** Although NR, as authors specifically addressed and co-facilitated a telehealth-based study, it was determined they had the relevant training. | **Collection method:** Interviews  **Analysis method:** Inductive thematic analysis  **Frameworks:** Critical realist epistemological position and theoretical interests in internalized stigma models | “Compassion-focused therapy seems to be a feasible and acceptable approach for transgender and gender non-conforming people. Group processes may be helpful in increasing self-acceptance. Further quantitative exploration of therapy process and outcomes is warranted.” |
| **First author, year:** Parameswaran, 2022  **Geographical location:** Utah  **Clinical setting:** Mental Health  **Methods type:** Qualitative  **Total enrolled patients/providers:**17 patients | **Focus:** Perinatal depression  **Aim:** To understand the impact of telemedicine as an intervention (via CBT, mindfulness) for peripartum women who were positive or high risk for peripartum depression. | **Inclusion:** Women who were pregnant or postpartum who screened positive for mild-moderate depression on the EPDS or were high risk for developing depression and had completed an 8-week videoconferencing intervention prior to interview participation. | **Demographics:**  *Age range:* 23-38 years  *Mean age:* 30.9 years  *Education:*  NR: 2  Some College: 1  College Graduate: 7  Post Graduate Master's Degree: 5  Doctorate Degree: 2  *Underrepresented groups:* No | **Type:** Video  **Scheduled:** Yes  **Training:** NR | **Collection method:** Focus groups  **Analysis method:** Thematic and content analyses  **Frameworks:** Grounded theory (Corbin and Strauss) | “This study provides growing evidence informed by perinatal women of positive experiences with engagement in a videoconference intervention for PD. Telehealth may be a reasonable and acceptable platform to increase access and retention for mental health services in childbearing women. Further, this pilot work showcases videoconferencing delivery for a wide range of effective and affordable mental health services in low-resource communities.” |
| **First author, year:** Moreau, 2018  **Geographic location:** United States  **Clinical setting:** Mental health  **Methods type:** Qualitative  **Total enrolled patients/providers:**40 providers | **Focus:** Mental health  **Aim:** To evaluate perceptions of the potential of telemental health to better serve a minority population with specific mental health needs, to inform future gender tailor or targeted expansion. | **Inclusion:** Located at one of nine VAMCs and sites in the Midwest, South, and Southeast; VAMCs had a female population that was 25% rural. | **Demographics:**  *Sex:*  Female: 34  Male: 6  *Care/staff type:*  Primary care: 12  Mental health: 10  Telehealth: 6  Gynecology: 3  Nursing: 3  Pharmacy: 2  Social work: 1  Other staff: 3  *Time at VA (years)*  <1: 3  1-5: 15  6-10: 8  ≥11: 6 | **Type:** Video  **Scheduled:** Yes  **Training:** NR | **Collection method:** Interviews  **Analysis method:** NR  **Frameworks:** NR | “Overall, our stakeholders saw telemental health as a good fit for helping to address the perceived needs of women veterans, especially in addressing the geographical barriers experienced by rural women and those with a limited ability to travel. These findings can help to inform gender-tailored expansion of telemental health within and outside of the VA.” |
| **First author, year:** Montesanti, 2022  **Geographical location:** Alberta, Canada  **Clinical setting:** Primary care  **Methods type:** Qualitative  **Total enrolled patients/providers:**24 providers | **Focus:** Domestic violence  **Aim:** Understand the perspectives of organizational leaders and service providers from the Alberta anti-violence sector during the COVID-19 pandemic on challenges in implementing virtual/remote-based services and interventions, as well as identifying policy-level actions to address these challenges and promote uptake of these services during the pandemic. | **Inclusion:** Providers working with and serving individuals at risk, experiencing, or survivors of domestic violence and sexual assault in Alberta, selected based on the diversity of client populations served. | **Demographics:**  *Role:*  MD, registered psychologists, crisis counselors, family physicians  *Underrepresented groups:* Rural focus | **Type:** NR  **Scheduled:** No  **Training:** NR | **Collection method:** Interviews  **Analysis method:** NR  **Frameworks:** NR | “Equity-focused policy and intersectional and systemic action are needed to enhance delivery and access to virtual interventions and services for domestic violence and sexual assault clients.” |
| **First author, year:** Madden, 2020  **Geographical location:** NYC, NY  **Clinical setting:** OB/GYN  **Methods type:** Triangulation Mixed Methods  **Total enrolled patients/providers:**11 providers | **Focus:** Prenatal care  **Aim:** To review adoption of telehealth for obstetric patients in a tertiary referral hospital and clinic system, to determine the degree to which prenatal care was transitioned to telehealth prenatal practices at two affiliated hospitals in NYC during the COVID-19 pandemic, and to describe provider experience with this transition. | **Inclusion:** OBGYN, maternal-fetal med, health clinic providers practicing telehealth at Columbia Irving - prenatal care. | **Demographics:**  *Role:*  MD, DNP, PA, RN | **Type:** Video  **Scheduled:** Yes  **Training:** Software trained in the week prior to adoption via a 15 minute video tutorial course and user guide with additional support. | **Collection method:** Interviews  **Analysis method:** Sensitivity analysis, thematic analysis  **Frameworks:** NR | “In transitioning to telehealth, operational challenges were more significant for health clinics than for MFM and generalist faculty practices with patients receiving public insurance experiencing greater difficulties and barriers to care. Additional resources on the patient and operational level were required to optimize attendance at in-person and video visits for clinic patients.” |
| **First author, year:** Lou, 2019  **Geographical location:** Denmark  **Clinical setting:** OB/GYN  **Methods type:** Qualitative  **Total enrolled patients/providers:**32 patients | **Focus:** DS diagnosis in pregnancy  **Aim:** To investigate the experience of receiving a prenatal DS diagnosis via phone, among couples who chose to terminate the pregnancy. | **Inclusion:** Couples who received a prenatal DS diagnosis of DS and decided to seek termination of pregnancy.  **Exclusion:** Termination of pregnancy due to uterine death, not own choice. | **Demographics:**  *Age range:* 22-47 years  *Education:*  Student: 1  1-10 years: 1  11-14 years: 12  > 15 years: 16  *Underrepresented groups:* No | **Type:** Telephone  **Scheduled:** Yes  **Training:** NR | **Collection method:** Interviews  **Analysis method:** Thematic analysis  **Frameworks:** NR | “A prearranged phone call was considered an acceptable and appropriate practice. However, some aspects of this practice (particularly related to the context of the call) showed to be less than optimal for the couples. To make sure that a diagnostic result is delivered in accordance with the couples' needs and requests, the context of the call could be addressed and agreed on in advance by physicians and couples.” |
| **First author, year:** Kerestes, 2021  **Geographical location:** United States (Georgia, Illinois, Maryland, Maine, New York)  **Clinical setting:** OB/GYN  **Methods type:** Qualitative  **Total enrolled patients/providers:**45 patients | **Focus:** Abortion  **Aim:** To explore the impact of direct-to-patient telemedicine abortion care upon participant ability to access abortion care outside REMS restrictions and the alternatives that were available if had they been unable to find this care. | **Inclusion:** English speaking individuals aged >18 years who had completed a TeleAbortion in the past 6 months. | **Demographics:**  *Age range:* 18-46 years  *Median age:* 29 years  *Education:* NR  *Underrepresented groups:* No | **Type:** Video  **Scheduled:** Yes  **Training:** NR | **Collection method:** Interviews  **Analysis method:** Qualitative content analysis, thematic analysis  **Frameworks:** NR | “Going to a clinic was a burden for participants, to the point where some would not have otherwise been able to get an abortion. Medication abortion by mail with telemedicine counseling was a highly acceptable alternative.” |
| **First author, year:** Ireland, 2020  **Geographical location:** Australia  **Clinical setting:** OB/GYN  **Methods type:** Qualitative  **Total enrolled patients/providers:**11 patients | **Focus:** Abortion care using telemedicine  **Aim:** To understand the experience of using telemedicine to access a medical abortion among rural women, and to gather information regarding preferences for face-to-face care or telemedicine. | **Inclusion:** Patients aged >18 years who experienced an abortion via telemedicine within 6 months prior to interview, residing in a rural area, were nulliparous or multiparous, of any ethnicity and from all states/territories. | **Demographics:**  *Age range:* 23-38 years  *Education:*  College or university-level education: 8  NR: 3  *Underrepresented groups:* Rural Focus  *Previous telehealth experience:* Yes | **Type:** Not specified  **Scheduled:** NR  **Training:** NR | **Collection method:** Interviews  **Analysis method:** Adapted framework analysis, feminist method  **Frameworks:** Integrated Patient-Centered Access to health-care model | “Telemedicine offers an innovative model for ensuring women’s access to medical abortion services in rural areas of Australia and likely has similar applicability to international non-urban contexts. Strategies are needed to ensure women with lower literacy and less favorable situational contexts, can equitably access abortion services through telemedicine.” |
| **First author, year:** Huang, 2022  **Geographical location:** Illinois  **Clinical setting:** Primary care  **Methods type:** Qualitative  **Total enrolled patients/providers:**40 providers | **Focus:** Contraceptive counseling  **Aim:** To identify clinician and staff perspectives on telehealth implementation regarding workflow, resources used, staff training, and other practice-level changes. | **Inclusion:** Provided contraceptive care in Illinois, purposefully selected based on diversity of geography, practice type, and clinician type. | **Demographics:**  *Clinical location:*  Planned Parenthood Illinois: 20  Community health centers: 11  Academic health centers: 4  Private practice: 5 | **Type:** Telephone and video  **Scheduled:** NR  **Training:** NR | **Collection method:** Interviews  **Analysis method:** Thematic content analysis  **Frameworks:** CFIR | “Illinois contraceptive care providers and staff wish to sustain telehealth for the long term, while also recommending specific improvements to patient communications, clinic operations, and access to supportive resources. Our study highlights considerations for clinics to optimize implementation of telehealth services for contraceptive care. Providers described the value of clear workflows to balance in person and telehealth visits, streamlined communications platforms, targeted patient outreach, training on providing virtual contraceptive care, and creative approaches to ensuring patient access to resources.” |
| **First author, year:** Hensel, 2021  **Geographical location:** Ontario, Canada  **Clinical setting:** Mental health  **Methods type:** Triangulation mixed methods  **Total enrolled patients/providers:**12 patients, 6 providers | **Focus:** Perinatal mental health  **Aim:** To explore the factors influencing engagement with videoconferencing for postpartum depressive and anxiety symptom focused psychotherapy, and identify key therapist considerations for patient engagement and the ongoing provision of psychotherapy via videoconferencing. | **Inclusion (patients):** Eligible patients were ≥18 years old, had an active email address, access to a video-enabled device and high-speed internet connection, displayed symptoms of depression/ anxiety, and had a referral to initiate psychotherapy or were receiving psychotherapy with 3+ months of planned treatment remaining.  **Inclusion (providers):** Performed virtual visits with women who met the inclusion criteria. | **Demographics (patients):**  *Mean age:* 34.5 years  *Education:* NR  *Underrepresented groups:* No  **Demographics (providers):** Master's trained social workers | **Type:** Video  **Scheduled:** Yes  **Training:** In-person training session and reference document | **Collection method:** Interviews  **Analysis method:** Thematic analysis using an inductive constant comparative approach  **Frameworks:** Comparative case study technique | “Therapists should consider that videoconferencing might not be appropriate for every patient; but in the right context and with appropriate therapeutic considerations, offering this treatment format may actually facilitate an individual's recovery. These findings can help to inform the future delivery of videoconferencing psychotherapy.” |
| **First author, year:** Henry, 2022  **Geographical location:** New South Wales, Australia  **Clinical setting:** Mental health care  **Methods type:** Embedded mixed methods  **Total enrolled patients/providers:**17 providers | **Focus:** Antenatal healthcare  **Aim:** To assess the effects of COVID-19 on DFV and mental health screening, as well as broader service provision from the perspective of local maternity service providers. | **Inclusion:** Registered maternity healthcare staff who worked in SESLHD Maternity during 2019 and 2020.  **Exclusion:** Not of allied health field or healthcare staff, did not work in these hospitals, or worked outside of the study time frames. | **Demographics:**  *Gender:*  Female: 14  Male: 2  Non-binary: 1  *Age range:* <25 and >55 years  *Role/service:*  MD, RN, social work, midwives, physiotherapy, genetic counselor, aboriginal health counselors | **Type:** Telephone and video  **Scheduled:** Yes  **Training:** NR | **Collection method:** Interviews  **Analysis method:** Thematic approach  **Frameworks:** NR | “While telehealth may have an ongoing, post-pandemic role in Australian maternity care, staff believe that this should be limited in scope, mostly for low-risk pregnancies. Women with high risk due to physical health or mental health, DFV, and/or other social concerns were considered unsuited to telehealth.” |
| **First author, year:** Hemming, 2021  **Geographical location:** Australia  **Clinical setting:** Oncology  **Methods type:** Qualitative  **Total enrolled patients/providers:**16 providers | **Focus:** Genetic counseling for ovarian cancer patients  **Aim:** Explore the experience of medical oncologists referring women with HGSOC for TGC and the reasoning for referring to TGC compared to their local clinical genetics service. | **Inclusion:** Medical oncologists who had referred ≥1 patient with HGSOC to the TGC service at the Peter MacCallum Cancer Centre, Australia between January 2016 and May 2017 for BRCA1/2 testing. | **Demographics:**  *Sex:*  Female: 10  Male: 6  *Patient referral setting:*  Metropolitan only: 4  Rural/regional only: 5  Metropolitan and rural/regional: 6 | **Type:** Telephone  **Scheduled:** Yes  **Training:** NR | **Collection method:** Interviews  **Analysis method:** Thematic analysis  **Frameworks:** Maxwell’s framework | “Overall, findings suggest that oncologists perceived telephone genetic counseling as an acceptable and useful healthcare service for patients with high-grade serous ovarian cancer. Moreover, they perceived telephone genetic counseling to be efficient, delivering convenient genetic counseling to patients.” |
| **First author year:** Grindlay, 2013  **Geographical location:** Iowa  **Clinical setting:** OB/GYN  **Methods type:** Qualitative  **Total enrolled patients/providers:**25 patients, 15 providers | **Focus:** Medical abortion  **Aim:** To evaluate the experiences of women and providers with telemedicine provision of medical abortion, particularly to understand the acceptability and impact of the telemedicine abortion service and upon patients, staff, and clinic operations. | **Inclusion (patients):** English speaking women who chose medical abortion and were eligible for the method (pregnant at ≤63 days gestation and no other standard contraindications), were ≥ 18 years old, were willing to participate and able to give informed consent.  **Inclusion (providers):** Doctors, advanced practice clinicians, nurses, medical assistants, and clinic managers at a Planned Parenthood of the Heartland clinic who were willing to participate and able to give informed consent. | **Demographics (patients):**  *Age range (years):*  18-24: 16  25-29: 5  30-40: 4  *Education:*  High school or less: 14  Some college or college degree: 11  *Underrepresented groups:* Rural Focus  **Demographics (providers):**  *Role:*  Medical assistants: 6  Clinic managers: 5  Physicians: 2  Nurses: 2 | **Type:** Video  **Scheduled:** Yes  **Training:** NR | **Collection method:** Interviews  **Analysis method:** Inductive coding  **Frameworks:** Grounded theory | “The findings from this study indicate that telemedicine can be used to provide medical abortion in a manner that is highly acceptable to patients and providers with minimal impact on the clinic. Practice Implications: This information demonstrates the feasibility of telemedicine to extend the reach of physicians and improve abortion access in rural settings.” |
| **First author, year:** Grindlay, 2017  **Geographical location:** Alaska  **Clinical setting:** OB/GYN  **Methods type:** Qualitative  **Total enrolled patients/providers:**8 providers | **Focus:** Medical abortion  **Aim:** Evaluate the experience of providers with telemedicine provision of medical abortion in Alaska, to expand the body of evidence on the experience and acceptability of telemedicine for medical abortion provision. | **Inclusion (providers):** Physicians, advanced practice clinicians, nurses, medical assistants/patient care coordinators, clinic managers, or counsellors at a clinic providing medical abortion via telemedicine. | **Demographics:**  *Role:*  Physicians: 4  Clinic managers: 2  Medical assistants/patient care coordinators: 2  *Prior experience with telemedicine:* 1 | **Type:** Video  **Scheduled:** NR  **Training:** NR | **Collection method:** Interviews  **Analysis method:** Inductive coding  **Frameworks:** Grounded theory | “These findings are consistent with previously published literature on medical abortion provided via telemedicine and indicate high acceptability among providers and the appropriateness for telemedicine application to this healthcare service.” |
| **First author, year:** Gorman, 2022  **Geographical location:** Oregon  **Clinical setting:** Oncology  **Methods type:** Triangulation mixed methods  **Total enrolled patients/providers:**10 patients | **Focus:** Sexual health in cancer survivorship  **Aim:** To examine the feasibility of a virtual MBI, Mindful After Cancer. | **Inclusion:** English speaking females, aged ≥18 years, who received a breast or gynecologic cancer diagnosis (stage 1-4) ≥1 year prior, had access to a device with internet access and 15-30 minutes available per day to engage with the intervention.  **Exclusion:** Stage 0 (DCIS) cancer. | **Demographics:**  *Age:* Reported for those who completed the intervention, but not according to those interviewed.  *Education*: Reported for those who completed the intervention, but according to those interviewed.  *Underrepresented groups:* No | **Type:** Video  **Scheduled:** Yes  **Training:** NR | **Collection method:** Interviews  **Analysis method:** Structural coding, constant comparison  **Frameworks:** NR | “Many cancer survivors experience sexual dysfunction and related distress after diagnosis and well after treatment ends, yet there are few interventions available. Improved access to effective interventions can improve the delivery of survivorship care and patient outcomes.” |
| **First author, year:** Gomez-Roaz, 2022  **Geograhpical location:** Chicago, Illinois  **Clinical setting:** OB/GYN  **Methods type:** Qualitative  **Total enrolled patients/providers:**40 patients | **Focus:** Post-partum  **Aim:** To elicit the perspectives of low-income postpartum individuals regarding healthcare experiences at a single academic center in the US during the COVID-19 pandemic. | **Inclusion:** English or Spanish-speaking pregnant/postpartum individuals, aged ≥16 years, with publicly funded prenatal care who received care at the Prentice Ambulatory Care Clinic, Chicago, Illinois and had given birth in the US during the first three months of the COVID-19 pandemic. | **Demographics:**  *Mean age:* 28 years  *Education:* NR  *Underrepresented groups:* Racial/ethnic marginalized group focus | **Type:** Telephone and video  **Scheduled:** Yes  **Training:** NR | **Collection method:** Interviews  **Analysis method:** Constant comparative method  **Frameworks:** NR | “Understanding the challenges experienced by low-income peripartum individuals as the COVID-19 pandemic evolves is critical to informing guidelines and diminishing inequities in healthcare delivery. Potential solutions that may mitigate limitations to care in the pandemic include emphasizing shared decision-making in care processes and developing communication strategies to improve telemedicine rapport.” |
| **First author, year:** Goldstein, 2021  **Geographical location:** Durham, NC  **Clinical setting:** Primary care  **Methods type:** Qualitative  **Total enrolled patients/providers:**23 patients | **Focus:** Patients at risk for CVD  **Aim:** To explore experiences with a tele-health-delivered intervention to reduce CVD risk. | **Inclusion:** Enrollment and completion of the CITIES trial, with preferential recruitment of females. | **Demographics:**  *Mean age (women):* 53 years  *Mean age (men):* 62 years  *Education:* NR  *Underrepresented groups:* Gender/ sexuality minority focus | **Type:** Telephone  **Scheduled:** Yes  **Training:** NR | **Collection method:** Interviews  **Analysis method:** Content analysis  **Frameworks:** NR | “Rapport building may differ between telehealth and in-person healthcare visits; our work highlights how men and women’s experiences can differ with telehealth care, and which can inform the development of future, purposeful rapport building activities to strengthen the clinician-patient interaction.” |
| **First author, year:** Ghidei, 2022  **Geographical location:** Alberta, Canada  **Clinical setting:** Mental health  **Methods type** Qualitative  **Total enrolled patients/providers:**24 providers | **Focus:** IPV  **Aim:** To understand the perspectives of providers who offer IPV counseling, and the facilitators/ obstacles patients may encounter while seeking virtual counseling due to the pandemic. | **Inclusion:** Stakeholders in the anti-violence sector in Alberta that provided IPV interventions virtually. | **Demographics:**  *Sex:*  Female: 17  Male: 4  Unknown: 3  *Race/ethnicity:*  White: 14  "Racialized": 7  *Time worked in the anti-violence sector:*  < 10 years: 15  *Role:*  Executive director: 5  Clinical director: 3  Program director: 3  Consultant: 3  Program or project manager/coordinator: 4  Physician: 2  Registered provisional psychologist: 2  Outreach counselor: 1  Clinical supervisor: 1  *Underrepresented groups:* Immigrants and refugees, Indigenous, rural/remote communities focus | **Type:** Telephone and video  **Scheduled:** Yes  **Training:** NR | **Collection method:** Interviews  **Analysis method:** Thematic analysis  **Frameworks:** Intersectional feminist theory | “Concepts of equity and safety are more complex for individuals affected by IPV, especially those who are socially disadvantaged. Service providers acknowledged pre-existing systemic and institutional barriers faced by underserved individuals impact their access to IPV interventions more generally. The COVID-19 pandemic further compounded these pre-existing challenges and hindered virtual access to IPV interventions. Service providers also highlighted the pandemic exacerbated structural vulnerabilities already experienced by underserved populations, which intensified the barriers they face in seeking help, and reduced their ability to receive safe and equitable interventions virtually.” |
| **First author, year:** Fix, 2020  **Geographical location:** Australia  **Clinical setting:** OB/GYN  **Methods type:** Qualitative  **Total enrolled patients/providers:**24 patients | **Focus:** Abortion  **Aim:** Explore the experiences of patients who obtained a medical abortion through MSA’s at-home telemedicine service and understand the barriers and facilitators of accessing care via this model. | **Inclusion:** Patients who had obtained a medical abortion from MSA via the at-home tele-medicine service and had completed the online English language self-administered survey. | **Demographics:**  *Age range:* 20-43 years  *Mean age:* 28 years  *Education:*  High school or less: 9  Some college or greater: 15  *Underrepresented groups:* No | **Type:** Telephone and video  **Scheduled:** Yes  **Training:** NR | **Collection method:** Interviews  **Analysis method:** Thematic analysis  **Frameworks:** NR | “At-home telemedicine model for medical abortion is a convenient and acceptable mode of service delivery that may reduce patient travel and out-of-pocket costs. Additional provider education about this model may be necessary in order to improve continuity of patient care. Further study of the impacts of this model on patients is needed to inform patient care and determine whether such a model is appropriate for similar geographical and legal contexts.” |
| **First author, year:** Ericson, 2017  **Geographical location:** Sweden  **Clinical setting:** OB/GYN  **Methods type:** Embedded mixed methods  **Total enrolled patients/providers:**26 patients | **Focus:** BF  **Aim:** Evaluate the experiences of mothers who received proactive BF support (receiving daily telephone call) compared with reactive support (mothers initiated the telephone call) after NICU discharge. | **Inclusion:** BF mothers of preterm infants (born <37 weeks GA) who spent >48 h in a NICU and were discharged from one of six NICUs in Sweden. | **Demographics:**  *Age:* NR  *Education:* NR  *Underrepresented groups:* No | **Type:** Telephone  **Scheduled:** Yes  **Training:** Provided although type was unclear | **Collection method:** Interviews  **Analysis method:** Thematic network analysis  **Frameworks:** NR | “There were positive aspects of both proactive (i.e., greater satisfaction and feelings of empowerment) and reactive support (i.e., the opportunity to call for support); however, the provision of reactive support alone may be in adequate for those with the greatest need for support as they are the least likely to access it.” |
| **First author, year:** Ehrenreich, 2019  **Geographical location:** Utah  **Clinical setting:** OB/GYN  **Methods type:** Qualitative  **Total enrolled patients/providers:**18 patients | **Focus:** Abortion access  **Aim:** To understand women’s experience with telemedicine for state mandated pre-abortion information sessions. | **Inclusion:** Pregnant women seeking an abortion in Utah who were state-mandated to undergo pre-abortion information sessions and who had chosen to include telemedicine information sessions.  **Exclusion**: Participated in in-person pre-abortion information sessions. | **Demographics:**  *Age range:* 19-40 years  *Education:*  Less than high school: 2  High school degree/GED: 3  Some college: 4  College degree: 6  Professional or advanced degree: 3  *Underrepresented groups:* No | **Type:** Video  **Scheduled:** Yes  **Training:** Formal training NR. Nurses conducted videoconferences using a state-mandated script and answered patient questions. | **Collection method:** Interviews  **Analysis method:** Thematic analysis  **Theoretical frameworks:** NR | “Telemedicine is highly acceptable to patients as a mode of attending state-mandated information visits for abortion. Although telemedicine does not eliminate the logistical and financial burdens previously found to be associated with Utah’s 72-hour waiting period and two-visit requirement, telemedicine may reduce the burdens associated with two-visit requirements for abortion and should be adopted in states that require face-to-face information sessions.” |
| **First author, year:** Demirci, 2019  **Geographical location:** Pennsylvania  **Clinical setting:** OB/GYN  **Methods type:** Embedded mixed methods  **Total enrolled patients/providers:**17 patients, 10 providers | **Focus:** BF  **Aim:** To understand and assess multiple perspectives of the feasibility, acceptability, strengths, and limitations of direct-to-consumer telelactation services for rural mothers. | **Inclusion (patients):** Mothers with variable patterns of telelactation use.  **Inclusion (providers):** Nurses and physicians caring for these mothers, and IBCLCs employed and referred by the telelactation provider. | **Demographics (patients):**  *Age:* NR  *Education:*  Less than high school: 4  High school/GED: 6  Some college: 3  Bachelors or more: 4  *Underrepresented groups:* Rural focus  *Previous telehealth experience:* Yes  **Demographics (providers):**  *Role:*  IBCLCs: 7  Ped: 1  Nurses: 2 | **Type:** Video  **Scheduled:** No  **Training:** NR | **Collection method:** Interviews  **Analysis method:** Thematic analysis  **Frameworks:** Miles and Huberman guided the analysis | “Among rural women who experience inequitable access to qualified BF support resources, DTC telelactation appears to be an acceptable delivery model for lactation assistance.” |
| **First author, year:** Cox, 2015  **Geographical location:** England  **Clinical setting:** Gyn-Oncology  **Methods type:** Qualitative  **Total enrolled patients/providers:**11 patients | **Focus:** Ovarian cancer survivors  **Aim:** To explore the experiences of women who engaged in long-term, nurse-led telephone follow-p after receiving ovarian cancer treatment. | **Inclusion:** Patients undergoing post treatment for ovarian cancer within one local cancer center , who had received ≥3 years of telephone follow-up. | **Demographics:**  *Age range:* 47-79 years  *Education:* NR | **Type:** Telephone  **Scheduled:** Yes  **Training:** NR | **Collection method:** Interviews  **Analysis method:** Interpretive phenomenological analysis  F**rameworks:** NR | “Nurse-led telephone follow-up was broadly recommended for women following treatment for ovarian cancer, particularly for those later on in the survivorship trajectory when focus may move from biomedical aspects of cure to holistic approaches to well-being. Remote interventions which provide a perception of a consistent and constant source of medical and psychosocial support may support adaption to cancer survivorship by enabling a reassertion of self and a rejection of patient identity.” |
| **First author, year:** Cocoran, 2021  **Geographical location:** United States  **Clinical setting:** Primary care  **Methods type:** Explanatory mixed methods  **Total enrolled patients/providers:**20 patients, 20 providers | **Focus:** CF  **Aim:** To explore the perceptions of CF healthcare providers and partners of women with CF on incorporating telehealth into routine CF health care. | **Patients:** NR  **Providers:** NR | **Demographics (patients):** NR  *Underrepresented groups:* Gender/ sexuality minority focus  **Demographics (providers):** NR | **Type:** Telephone and video  **Scheduled:** NR  **Training:** NR | **Collection method:** Interviews  **Analysis method:** Braun and Clarke’s six steps to thematic analysis: familiarize with the data, generate initial codes, search for themes, review themes, define themes, write-up analysis  **Frameworks:** NR | “Results from this study highlighted the positive value of telehealth. Telehealth presents as a potential alternative to delivering outpatient care for people with chronic illnesses beyond the pandemic.” |
| **First author, year:** Christiansen, 2022  **Geographical location:** Denmark  **Clinical setting:** Gyn-Oncology  **Methods type:** Qualitative  **Total enrolled patients/providers:**32 patients | **Focus:** Gynecologic cancer  **Aim:** To explore the experiences and perspectives of patients with gynecological cancer whose outpatient appointments were re-scheduled from face-to-face to digital consultations during the COVID-19 pandemic. | **Inclusion:** Patients fluent in Danish, aged >18 years, with an active email address, a diagnosis of ovarian/ cervical/ endometrial/ vaginal/ vulvar cancer, and active oncological treatment or follow up.  **Exclusion:** First appointment involved a physical exam or cognitive/ psychiatric impairments | **Demographics:**  *Age range:* 28-75 years  *Mean age:* 53 years  *Education:*  Primary: 1  High school: 1  Short (<3 years): 7  Medium: 13  Higher: 9  NR: 1  *Underrepresented groups:* No | **Type:** Telephone and video  **Scheduled:** Yes  **Training:** NR | **Collection method:** Interviews  **Analysis method:** Thematic analysis  **Frameworks:** NR | “Digital consultations were an accepted alternative during COVID-19. Even though this temporary solution was deemed to be beneficial for practical reasons, patients also experienced digital consultations to be impersonal. A key message is that face-to-face encounters create the foundation to establish a trusting relationship from where a valuable dialogue arises. Digital consultations should therefore be implemented with caution since no one-size-fits-all model is recommended. Among patients with gynecological cancer, however, digital technologies represent a promising and flexible method depending on the purpose of consultations, patient preferences, and needs.” |
| **First author, year:** Boydell, 2021  **Geographical location:** Edinburgh, Scotland  **Clinical setting:** OB/GYN  **Methods type:** Qualitative  **Total enrolled patients/providers:**20 patients | **Focus:** Abortion up to 12-week gestation  **Aim:** Explore the acceptability of the telemedicine abortion consultation/clinical support, views on not receiving a pre-abortion ultrasound scan, experiences of self-administering mifepristone and misoprostol, and the impact of receiving telemedicine in response to COVID-19 upon abortion care. | **Inclusion**: Patients obtaining medical abortion under Ground C of the 1967 Abortion Act 22 (i.e. not for medical conditions or fetal abnormality), who were eligible for medical abortion at home, aged ≥18 years; and fluent in speaking, reading and understanding English. | **Demographics:**  *Age group (years):*  18-25: 8  26-32: 7  33-39: 5  *Education:*  Secondary school: 4  School or college 6th form: 1  College of further education: 4  Polytechnic or University: 11  *Underrepresented groups:* No | **Type:** Telephone  **Scheduled:** Yes  **Training:** NR | **Collection method:** Interviews  **Analysis method:** Thematic analysis  **Frameworks:** Framework analytic method | “This research demonstrates support for the continuation of telemedicine abortion services beyond the temporary arrangements in place during COVID-19, and lends weight to the argument that offering the option of telemedicine abortion care can enable women to access this essential health service.” |
| **First author, year:** Bogulski, 2022  **Geographical location:** Little Rock, Arkansas  **Clinical setting:** OB/GYN  **Methods type:** Triangulation mixed methods  **Total enrolled patients/providers:**36 patients | **Focus:** BF post-partum women  **Aim:** To identify facilitators and barriers to the use of two telehealth service modes, and compare the differences between each for providing tele-lactation services on BF knowledge and intention, perceived social support, and 3-month BF continuation behavior. | **Inclusion:** Postpartum women aged ≥18 years from two medical centers who had a baby which was expected to be discharged with them, a device with a camera/ microphone, and home internet access.  **Exclusion:** Contraindications for BF per their hospital records. | **Demographics:**  *Age group (years):*  18-19: 2  20-29: 14  30-39: 27  *Education:*  Less than high school: 5  Some college: 13  Bachelor’s degree: 10  Graduate degree: 15  *Underrepresented groups:* Rural focus | **Type:** Telephone and video  **Scheduled:** Yes  **Training:** NR | **Collection method:** Interviews  **Analysis method:** Directed content analysis  **Frameworks:** NR | “We found that both telephone-only and audio-visual delivery of tele-lactation services were equally effective. Both methods of tele-lactation services should be considered by health care providers to encourage and sustain BF behavior in mothers.” |
| **First author, year:** Beaver, 2010  **Geographical location:** United Kingdom  **Clinical setting:** Oncology  **Methods type:** Qualitative  **Total enrolled patients/providers:**28 patients, 4 providers | **Focus:** Breast cancer  **Aim:** To understand the views and experiences of patients who received or specialized BCNs who provided telephone follow-up consultations during the Beaver et al. study. | **Inclusion (patients):** A random sample of ~20% of patients who received telephone follow-up for the duration of the study period.  **Inclusion (providers):** BCNs who had delivered the intervention. | **Demographics (patients):**  *Age range:* 48-80  *Mean age:* 61 years  *Education:* NR  *Underrepresented groups:* No  **Demographics (providers):**  Bachelor’s degree: 4  Postgraduate master’s degrees: 3  Courses/modules in breast care nursing and counselling: 4  Experience running nurse-led hospital clinics: 3 | **Type:** Telephone  **Scheduled:** Yes  **Training:** Seven specialist BCNs undertook a period of training on administration of the telephone intervention, although four administered the telephone intervention in the main trial. | **Collection method:** Interviews  **Analysis method:** Manifest content analysis  **Frameworks:** NR | “Positive views on telephone follow-up were reported. An experienced and skilled practitioner, with effective communication skills, is required to deliver the intervention and a period of training is recommended. Nurse-led telephone interventions have a broader applicability to other disease conditions, although more work is needed to develop appropriate interventions and evaluate their effectiveness.” |
| **First author, year:** Beatty, 2022  **Geographical location:** Johnson City, TN  **Clinical setting:** Primary Care  **Methods type:** Qualitative  **Total enrolled patients/providers:**20 providers | **Focus:** Contraceptive care  **Aim:** Examine the provision of telehealth contraceptive services during the initial months of the COVID-19 pandemic among HDs in two Southern US states with centralized and largely centralized governance structures, and to identify telehealth facilitators and barriers. | **Inclusion:** HD providers at clinics that offered any contraceptive service in 2019 (the year preceding the survey). | **Demographics:**  *Role:*  District clinical directors, senior nurse practitioners, nurse supervisor, preventive health nurses, site supervisors, program managers | **Type:** NR  **Scheduled:** NR  **Training:** NR | **Collection method:** Interviews  **Analysis method:** Rapid analysis  **Frameworks:** NR | “Implementation of telehealth for contraceptive services varied between state HD agencies in the early phase of the pandemic. Medicaid reimbursement policy and directives from HD agency leadership are key to telehealth service provision among HDs in centralized states.” |
| **First author, year:** Beatty, 2022  **Geographical location:** Alabama and South Carolina  **Clinical setting:** Primary Care  **Methods type:** Triangulation mixed methods  **Total enrolled patients/providers:**25 providers | **Focus:** Contraceptive care  **Aim:** To provide information about telehealth provision for rural and urban FQHCs and inform programs designed to increase and maintain telehealth access for contraceptive care in safety-net clinics. | **Inclusion:** Clinic-, corporate-, and system-level employees of rural and urban FQHCs key informants. | **Demographics:**  *Clinic-level interviewee roles:*  Reproductive health manager, practice manager, family nursing supervisor, registered nurse clinical coordinator, certified medical assistant, licensed practical nurse, women’s health nurse practitioner.  *System-level interviewee roles:*  Project managers, chief executive officer, clinic operations directors,  directors of patient services.  *Underrepresented groups*: Rural focus | **Type:** Telephone and video  **Scheduled:** NR  **Training:** NR | **Collection method:** Interviews  **Analysis method:** rapid analytic approach (Hamilton), thematic analysis  **Frameworks:** NR | “Key facilitators of telehealth were reimbursement policy, electronic infrastructure and technology, and funding for technology. Barriers included challenges with funding for telehealth, limited electronic infrastructure, and reduced staffing capacity. Differences in telehealth service provision for contraceptive care between rural and urban FQHCs highlight the need for supportive strategies to increase access to care for low-income rural populations, particularly in AL and SC. It is essential for public and private entities to support the implementation and continuation of telehealth among rural clinics, particularly, investing in widespread and clinic-level electronic infrastructure and technology for telehealth, such as broadband and electronic health record systems compatible with telehealth technology.” |

ACP: advanced clinical practitioners, ACT: acceptance commitment therapy, AL: Alabama, BA: behavioral activation, BCN: breast care nurses, BF: breastfeeding, BMI: body mass index, BRCA: breast cancer gene, CBOC: community-based outpatient clinic, CBT: cognitive behavioral therapy, CF: cystic fibrosis, CFIR: consolidated framework for implementation research, CITIES: Cardiovascular Intervention Improvement Telemedicine Study, CNS: certified nursing assistant, CVD: cardiovascular disease, DCIS: ductal carcinoma in situ, DFV: domestic and family violence, DNP: doctor of nursing practice, DTC: direct to consumer, DS: Down Syndrome, ED: emergency department, EPDS: Edinburgh postnatal depression scale, EPIS: exploration, preparation, implementation, sustainment, FQHC: federally qualified health center, GA: gestational age, GED: general educational development, HBOC: hereditary breast and ovarian cancer, HD: health department, HGSOC: high-grade serious ovarian cancer, IBCLC: international board certified lactation consultant, IPV: intimate partner violence, LARC: long-acting reversible contraception, MBI: mindfulness based intervention, MD: Doctor of medicine, MS: multiple sclerosis, MSA: Marie Stopes Australia, NASSS: non-adoption, abandonment, scale-up, spread, sustainability framework, NR: not reported, NHS: National Health Service, NICU: neonatal intensive care unit, NSP: non specialist provider, NY: New York, OB/GYN: obstetrics and gynecology, PA: physician assistant, PCMHS: primary care mental health service, PCP: primary care physician, PNMH: perinatal mental health service, PTSD: post-traumatic stress disorder, REMS: risk evaluation and mitigation strategy, RN: registered nurse, SANE: sexual assault nurse examiner, SC: Southern Carolina, SESLHD: south eastern Sydney local health district, SUMMIT: scaling up maternal mental healthcare by increasing access to treatment, TFU: telephone follow up, TGC: telephone genetic counselling, US: United States, UK: United Kingdom, VA: Veterans Affairs, VAMC: Veterans Affairs Medical Center, VITAL: Video coaching to assist lifestyle
