# Supplementary material for: Experiences Receiving and Delivering Virtual Health Care For Women: Qualitative Evidence Synthesis
Source: J Med Internet Res. 2025 May 15;27:e68314. doi: 10.2196/68314 (PMC12123244; doi:10.2196/68314)
Supplement: Multimedia Appendix 6 [file jmir_v27i1e68314_app6.docx]

| **Author**  **Year** | **Was there a clear statement of the aims of the research?** | **Is a qualitative methodology appropriate?** | **Was the research design appropriate to address the aims of the research?** | **Are the study’s theoretical underpinnings clear, consistent and conceptually coherent?** | **Was the recruitment strategy appropriate to the aims of the research?** | **Was the data collected in a way that addressed the research issue?** | **Has the relationship between researcher and participants been adequately considered?** | **Have ethical issues been taken into consideration?** | **Was the data analysis sufficiently rigorous?** | **Was there a clear statement of the findings?** |
| --- | --- | --- | --- | --- | --- | --- | --- | --- | --- | --- |
| Allison 2023 | Yes | Yes | Yes | Somewhat | Yes | Yes | No | Somewhat | Yes | Yes |
| Beatty 2022  J Rural Health | Yes | Yes | Yes | Can't tell | Somewhat | Yes | Can't tell | Yes | Yes | Yes |
| Beatty 2022  J. Public Health Mgmt. & Pract | Yes | Yes | Yes | Can't tell | Yes | Somewhat | Can't tell | Yes | Yes | Somewhat |
| Beaver 2010 | Somewhat | Yes | Yes | Can't tell | Yes | Yes | No | Yes | Yes | Yes |
| Bogulski 2022 | Yes | Yes | Yes | Can't tell | Somewhat | Yes | Somewhat | Yes | Yes | Yes |
| Boydell 2021 | Yes | Yes | Yes | Somewhat | Yes | Yes | Somewhat | Yes | Yes | Yes |
| Buse 2022 | Yes | Yes | Somewhat | Can't tell | Somewhat | Yes | No | Yes | Somewhat | Yes |
| Christiansen 2022 | Yes | Yes | Yes | Yes | Yes | Yes | Somewhat | Yes | Yes | Yes |
| Corcoran 2021 | Yes | Yes | Yes | Somewhat | Yes | Yes | Somewhat | Yes | Yes | Yes |
| Cox 2015 | Yes | Yes | Yes | Yes | Yes | Yes | No | Yes | Yes | Yes |
| Davenport 2023 | Yes | Yes | Somewhat | Yes | Yes | Yes | Somewhat | Yes | Yes | Yes |
| Demirci 2019 | Yes | Yes | Yes | Can't tell | Somewhat | Somewhat | Can't tell | Yes | Somewhat | Yes |
| Ehrenreich 2019 | Yes | Yes | Yes | Can't tell | Yes | Yes | No | Yes | Yes | Yes |
| Ericson 2017 | Yes | Yes | Yes | Can't tell | Yes | Somewhat | Yes | Yes | Yes | Yes |
| Fix 2020 | Yes | Yes | Yes | Can't tell | Yes | Yes | No | Yes | Can't tell | Yes |
| Ghidei 2022 | Yes | Yes | Yes | Can't tell | Yes | Yes | Yes | Yes | Yes | Yes |
| Goldstein 2014 | Yes | Yes | Yes | Yes | Yes | somewhat | Yes | Yes | Yes | Yes |
| Gomez-Roas 2022 | Yes | Yes | Yes | Yes | Yes | Yes | Can't tell | Yes | Yes | Yes |
| Gorman 2022 | Yes | Yes | Yes | Yes | Yes | Yes | Can't tell | Yes | Yes | Yes |
| Grindlay 2017 | Yes | Yes | Yes | No | Somewhat | Somewhat | No | Somewhat | Somewhat | Yes |
| Grindlay 2013 | Yes | Yes | Yes | Can't tell | Yes | Somewhat | No | Yes | Can't tell | Somewhat |
| Hemming 2021 | Yes | Yes | Yes | Somewhat | Yes | Yes | No | Yes | Somewhat | Somewhat |
| Henry 2022 | Yes | Yes | Yes | No | Yes | Yes | Yes | Yes | Somewhat | Somewhat |
| Hensel 2021 | Yes | Yes | Yes | Somewhat | Somewhat | Yes | Can't tell | Yes | Somewhat | Yes |
| Howard 2023 | Yes | Yes | Yes | Can't tell | Yes | Somewhat | No | Somewhat | Yes | Yes |
| Howell 2023 | Yes | Yes | Yes | Yes | Yes | Yes | Yes | Yes | Yes | Yes |
| Huang 2022 | Yes | Yes | Yes | Yes | Yes | Yes | Can't tell | Yes | Somewhat | Yes |
| Ireland 2020 | Yes | Yes | Yes | Yes | Yes | Yes | Can’t tell | Yes | Somewhat | Somewhat |
| Kerestes 2021 | Yes | Yes | Somewhat | Can't tell | Yes | Yes | Can't tell | Yes | Somewhat | Yes |
| Kissler 2023 | Yes | Yes | Yes | Can't tell | Yes | Yes | Yes | Yes | Yes | Yes |
| Kozica-Olenski 2023 | Yes | Yes | Yes | Yes | Yes | Yes | No | Yes | Yes | Yes |
| Lou 2019 | Yes | Yes | Somewhat | Can't tell | Yes | Yes | No | Yes | Yes | Yes |
| Madden 2020 | Yes | Yes | Can't tell | Can't tell | Yes | Somewhat | Can't tell | Yes | Somewhat | Yes |
| Montesanti 2022 | Yes | Yes | Yes | Yes | Yes | Yes | Can't tell | Yes | Somewhat | Yes |
| Moreau 2018 | Yes | Yes | Somewhat | Yes | Yes | Yes | Can't tell | Yes | Yes | Yes |
| Nguyen-Grozavu 2023 | Yes | Yes | Yes | Can't tell | Yes | Yes | No | Somewhat | Yes | Yes |
| Parameswaran 2022 | Yes | Yes | Yes | Can't tell | Yes | Yes | Can't tell | Yes | Yes | Yes |
| Pipkin 2022 | Yes | Yes | Yes | Yes | Yes | Yes | Yes | Yes | Yes | Yes |
| Reynolds-Wright 2022 | Yes | Yes | Yes | Can't Tell | Yes | Yes | Somewhat | Yes | Yes | Yes |
| Sessanna 2021 | Yes | Yes | Yes | Somewhat | Yes | Yes | Somewhat | Yes | Yes | Yes |
| Silverio 2021 | Yes | Yes | Yes | Yes | Yes | Yes | Yes | Somewhat | Yes | Yes |
| Simon 2023 | Yes | Yes | Yes | Can't tell | Yes | Can't tell | No | Somewhat | Somewhat | Somewhat |
| Singla 2022 | Yes | Yes | Yes | Can't tell | Somewhat | Yes | Yes | Somewhat | Yes | Yes |
| Song 2022 | Yes | Yes | Yes | Can't tell | Yes | Yes | No | Somewhat | Somewhat | Somewhat |
| Spiby 2014 | Yes | Yes | Yes | No | Can't tell | Yes | Can't tell | Somewhat | Yes | Somewhat |
| Srinivasulu 2022 | Yes | Yes | Somewhat | No | Yes | Yes | Can't tell | Somewhat | Yes | Yes |
| Sterba 2023 | Yes | Yes | Yes | Yes | Yes | Yes | Somewhat | Yes | Yes | Yes |
| Tan 2013 | Yes | Yes | Somewhat | Can't tell | Somewhat | Yes | Somewhat | No | No | Somewhat |
| Vincze 2018 | Yes | Yes | Yes | Can't tell | Yes | Yes | Yes | Yes | Yes | Yes |
| Williamson 2018 | Yes | Yes | Yes | Can't tell | Yes | Somewhat | Somewhat | Yes | Yes | Yes |
| Zilliacus 2010 | Yes | Yes | Yes | Can't tell | Yes | Yes | Yes | Yes | Yes | Yes |
