# Supplementary material for: Experiences Receiving and Delivering Virtual Health Care For Women: Qualitative Evidence Synthesis
Source: J Med Internet Res. 2025 May 15;27:e68314. doi: 10.2196/68314 (PMC12123244; doi:10.2196/68314)
Supplement: Multimedia Appendix 7 [file jmir_v27i1e68314_app7.docx]

| **Summary of finding** | **Contributing studies** | **Methodological limitations** | **Coherence** | **Adequacy** | **Relevance** | **CERQual assessment** | **Explanation of assessment** |
| --- | --- | --- | --- | --- | --- | --- | --- |
| ***Adopters*** | | | | | | | |
| **Translation of existing interpersonal skills (patients)**: Providers had mixed perceptions of women’s engagement in virtual care compared to in-person. Women’s sense of confidence in their abilities to complete the required tasks to participate in telehealth were facilitated by previous use of virtual platforms (e.g., use of zoom). | 6684, 7450, 8936, 9148, 9189 | No or very minor concerns. | Moderate concerns: inconsistently mentioned and primarily reported by providers about patients. | Moderate concerns: only mentioned in 5 studies and descriptions vague. | Moderate concerns; little data from patients and data from limited clinical context. | Low confidence | 5 studies with no or very minor methodologic concerns; moderate concerns about coherence, adequacy and relevance due to limited support that was mostly from providers and across a narrow clinical spectrum. |
| **Translation of existing interpersonal skills (clinicians)**: Clinicians who had existing skills related to showing empathy, demonstrating clinical knowledge, and building rapport in-person were often able to translate those skills to virtual settings which facilitated the success of virtually-delivered care. | 674, 687, 1014, 1709, 1904, 2233, 3766, 8157, 8795, 8916, 9142, 9148, 9189, 9290 | Moderate concerns due to 8 studies with at least somewhat unclear underlying theory, 3 with some recruitment concerns, 10 with at least some lack of clarity on researcher-participant relationship, and 2 with somewhat unclear analytic rigor. | Minor concerns: primarily reported by patients about providers. | No or very minor concerns. | No or very minor concerns. | Moderate confidence | 14 studies with moderate concerns due to issues with theoretical underpinnings, recruitment, researcher-participant relationship, and data rigor; minor concerns about coherence and no or very minor concerns about relevance and adequacy. |
| **Negative impact on utilizer’s well-being (patients):** Some patients using virtual care experienced fatigue, distress, and anxiety; the need to be more proactive and self-aware and added technological challenges could be intolerable while others didn’t mind longer waits when virtual. Some patients experienced dysphoria when seeing themselves on camera. | 1522, 2965, 3449, 6393, 6684, 9142, 9189, 9541 | Minor concerns; 1 study with a somewhat appropriate design; 3 with somewhat or can’t tell theoretical underpinnings, 2 with somewhat appropriate recruitment, 6 with some or less detail about researcher-participant relationship; 2 with somewhat rigorous data analysis. | No or very minor concerns. | No or very minor concerns. | No or very minor concerns. | High confidence | 8 studies with minor methodologic concerns unlikely to limit confidence in finding; no or very minor concerns regarding coherence, adequacy, and relevance. |
| **Negative impact on utilizer’s well-being (clinicians):** Several articles reported increased fatigue, stress and burnout for clinicians using virtual care. Contributing experiences included a sense of isolation, low competence with technology, and dysphoria with viewing self on screen. Some felt decreased stress when working from home. | 687, 3445, 3662, 5068, 5623, 6684, 7450, 9148, 9396 | Moderate concerns due to 6 studies with limited information about theoretical underpinnings, 7 with somewhat or greater concerns about data collection, 7 with limited information about researcher-participant relationship, 2 with some concerns about ethics, and 5 with some concerns about analytic rigor. | No or very minor concerns. | No or very minor concerns. | No or very minor concerns. | Low confidence | 9 studies with moderate methodologic concerns with data collection, unclear researcher-participant relationship and analytic rigor; no or very minor concerns about coherence, adequacy, and relevance. |
| **Mixed impact of virtual setting on patient privacy and safety:** Home context drives privacy and safety concerns for women using virtual care. For example, some women feel more comfortable sharing sensitive information from home. However, others have difficulty finding space free from distractions, such as children. Safety concerns included both online security and the presence of perpetrators of violence in the home. | 1014, 1522, 2233, 2817, 2965, 3063, 3066, 3445, 3449, 3662, 4221, 5623, 6393, 7450, 8487, 8936, 9036, 9142, 9148, 9396, 9541 | Moderate concerns due to 13 studies with at least somewhat unclear theoretical underpinnings, 2 with some recruitment issues, 3 with some or unclear data collection, 16 with only some or less information about researcher-participant relationship, 4 with some lack of ethics clarity, and 8 with some concerns about data rigor. | No or very minor concerns. | No or very minor concerns. | No or very minor concerns. | Moderate confidence | 21 studies with moderate methodologic concerns due to limited information about researcher-participant relationship, lack of clarity on ethical considerations, data collection, and recruitment and 8 studies with some analytic rigor concerns. However, no or very minor concerns for coherence, adequacy and relevance including broad support by patient and provider data across all clinical contexts. |
| ***Values*** | | | | | | | |
| **Convenience and cost savings:** Virtually-delivered care provides significant convenience to women through reducing travel burden, requiring less time away from work, school and caregiving commitments, and less cost burden related to gas money, childcare expenses, and lost income. Women who had caregiving responsibilities, mobility-limiting disabilities, limited transportation access, a high healthcare burden, full-time work and/or school commitments, or those experiencing life chaos or living far from health care resources benefited the most from this convenience and cost savings. | 676, 687, 921, 1014, 1522, 1658, 1709, 1904, 2233, 2545, 2965, 3063, 3066, 3426, 3449, 3662, 3766, 4221, 5648, 6217, 7122, 7324, 7450, 7510, 7715, 8157, 8487, 8795, 8936, 9036, 9142, 9148, 9189, 9190, 9290, 9541 | Minor concerns due to 4 studies with some concern about design, 14 with unclear or only somewhat clear theoretical underpinnings, 3 with only somewhat appropriate recruitment, 10 with inadequate or no consideration of researcher-participant relationship, 4 with somewhat insufficiently rigorous analysis. | No or very minor concerns about coherence. | No or very minor concerns about adequacy. | No or very minor concerns about relevance. | High confidence | 36 studies with minor methodologic concerns. Data from both patients and providers from across diverse clinical service types. No or very minor concerns about coherence and adequacy. |
| **Importance of clinician continuity:** Providing virtual care within an existing provider relationship is key for patient comfort as is virtual care delivered by the same clinician over multiple visits (e.g., mental health treatment or surveillance during breast cancer survivorship care). | 687, 921, 1522, 1709, 1904, 2337, 3449, 5648, 7324, 7486, 9189, 9541 | Moderate concerns due to 2 studies with somewhat appropriate methods, 7 with unclear theoretical underpinnings, 6 with unclear recruitment methods, 2 with some data collected issues, 9 with unclear researcher-participant relationship, and 3 with only somewhat rigorous analysis. | No or very minor concerns. | Minor adequacy: mentioned but not expanded upon or emphasized, richness of relevant data was less. | Minor concerns due to unclear relevance to diverse clinical settings/context – not all represented in data; fewer provider supporting data than patient. | Moderate confidence | 12 studies with moderate methodologic concerns primarily related to recruitment methods, data collection and analytic rigor; minor concerns about adequacy and relevance, and no or very minor concerns coherence. |
| **Value of modality choice:** No modality is right for every woman; offering patients autonomy around selection of visit modality was highly valued and seen by some as manifestation of delivering patient-centered care. | 1522, 1904, 2233, 2545, 2965, 3063, 3426, 3449, 3662, 7122, 7450, 8487, 9142, 9148, 9290, 9541 | Minor concerns due to 2 studies with somewhat appropriate design, 5 with unclear theoretical underpinning, 1 with some concerns about recruitment and data collection, and 6 with unclear researcher-participant relationship, 2 studies with limited analytic rigor. | No or very minor concerns. | No or very minor concerns. | No or very minor concerns. | High confidence | 16 studies with minor methodologic concerns; no or very minor concerns about coherence, adequacy, and relevance. |
| **Loss of collateral benefits:** Some women missed collateral interpersonal interactions typically occurring during in-person visits such as those with members of the extended clinical team (e.g., intake nurses and front desk staff) and other patients. | 687, 1522, 2922, 2965, 6217, 7122, 7715, 8936, 9142, 9290 | Moderate concerns due to 2 studies with design issues, 4 with unclear theory base, 1 with recruitment, data collection concerns and ethics evaluation, all 10 with lack of clarity on researcher-participant relationship, and 2 with some limited analytic rigor. | Moderate concerns: fit is looser for this finding, little specificity on what was missed re: collateral benefits. | Minor concerns: supporting data not as rich in detail or extensive. | Moderate concerns: may not be relevant to settings that are primarily delivered via telehealth and may have been heightened due to concurrence with COVID restrictions. | Low confidence | 10 studies with moderate methodologic concerns largely related to lack of theoretical underpinning and design appropriateness; moderate concerns for coherence and relevance; minor concerns for adequacy. |
| ***Wider System*** | | | | | | | |
| **Clinician and clinical deserts:** Areas with limited or no access to women’s health clinical resources due to rurality or distance facilitated use of virtually-delivered care. This was particularly relevant for time-sensitive, women-specific care needs such as pregnancy, abortion care, or sexual assault nurse examinations. In addition, clinical deserts may be particularly impactful for women with key intersectional identities (e.g., IPV services for LGBTQ+, women with a disability, or women referring a race-concordant provider). | 676, 1014, 1904, 2233, 2545, 2817, 3063, 3066, 3426, 3445, 3766, 4956, 5648, 6684, 7122, 7450, 7486, 8795, 8916, 9142, 9148, 9189, 9396 | Moderate concerns due to 2 studies with somewhat appropriate design, 17 with limited information about theoretical underpinnings, 3 with some concerns about recruitment, 4 regarding data collection, 16 with limited detail about researcher-participant relationship, 3 with limited ethics considerations, and 7 with analytic rigor limitations. | No or very minor concerns. | No or very minor concerns. | No or very minor concerns. | Moderate confidence | 23 studies with moderate methodologic concerns primarily downgraded for issues with recruitment, data collection, limited detail about researcher-participant relationship, and analytic rigor. No or very minor concerns about relevance, coherence, and adequacy. |
