# Supplementary material for: Experiences Receiving and Delivering Virtual Health Care For Women: Qualitative Evidence Synthesis
Source: J Med Internet Res. 2025 May 15;27:e68314. doi: 10.2196/68314 (PMC12123244; doi:10.2196/68314)
Supplement: Multimedia Appendix 8 [file jmir_v27i1e68314_app8.docx]

| **Finding (certainty of evidence^a^)** | **Description** | **Illustrative quotes** |
| --- | --- | --- |
| ***Technology*** | | |
| **Challenges to emotional connections** | Both women and clinicians noted that in-person visits were preferred for establishing rapport and were more appropriate during difficult or emotionally-charged conversations (e.g., new cancer diagnosis, discussing sexual dysfunction, or after sexual assault). Using technology could enhance or diminish connection in the group setting but women were often still able to build connections virtually. | “*It is not the same thing when they see you through video when you have cancer and not have them extend their hand. For them to tell you ‘To be honest, these results did not turn out how we expected’. That emotion is not the same one that they transmit through a phone as opposed to a face to face meeting.*”^206^ *–* Hispanic woman with breast cancer  “*You have to wonder, although we’re trying to do the best we can, is providing virtual care really providing that care? Because we’re wired for connection, we’re wired for…when you’re sitting across the table from somebody, I think you can be more empathetic because that person is sitting right in front of you than on computer, you are quite removed*”^207^ – Clinician with intimate partner violence and sexual assault service organization |
| **Amplifying communication barriers** | Women who were hard of hearing or Deaf or those for whom English was not their preferred language faced additional barriers to virtual care as translation services were often difficult to incorporate into virtual visits. One example of communication being challenged by virtual care delivery was emergency care for women who had been sexually assaulted. | *"…if you come into the clinic you can see visually, but you trying to explain to somebody over the phone with an interpreter, how you use some type of birth control or what you need to or how you need to do it, sometimes is not as helpful."*^208^ *–* Women's health clinician  *"I do think [people who require interpreters] would benefit from…you learn so much more from a face-to-face…you can trigger…pick up uncertainty, even sometimes for the poor interpreters there's not always words in their language…or they're not familiar with, that we're trying to think of alternatives in English for them to translate and that can be a major faff on the phone."*^209^ *–* Telemedicine medical abortion care provider |
| **Training for women** | Women desired technical and communication skills to facilitate the receipt of virtual care. Training on how to communicate physical aspects of their health experience was important when women could not demonstrate or show something in-person. Patient-specific training materials should be tailored to learning style (e.g., literature or pictures) and be available in multiple languages. Clinicians felt patients should also receive training on best practices for engaging during a virtual visit (e.g., not multitasking). | “… *there were definitely people who weren't familiar with Zoom. And so the navigators had some scripting on testing…send them, hey, here’s a link to how to use Zoom, you want to practice that now…. So sometimes that could be a barrier, for sure*.”^210^ – Clinician providing perinatal mental health |
| **Training for clinicians** | Clinicians *and* staff need to learn how to build and maintain rapport, effectively communicate in virtual settings, provide high-quality clinical services on a telehealth platform, select a modality that is appropriate for the patient’s clinical condition and reason for visit, and conduct a clinical assessment while addressing limitations of virtual modalities (e.g., having limited visual cues, non-verbal communication). | "*It wasn't well received in the beginning. It is different now. It's nobody's favorite thing to have to do because nobody wants to get in front of the camera and perform. But it's much better. We're all more trained in it and more comfortable with it*.”^211^ *–* Emergency department staff using virtual sexual assault nurse examiner consultation with women  *“I struggle with technology because it’s not natural to me. If, all of a sudden, my screen froze…I have no idea how to troubleshoot or fix this problem. And because it feels like I’m a stranger in a strange land, I would rather not do it in the first place. So, I think that for me, it wasn’t even so much the interaction online. It was this technology feels way too scary and big and overwhelming. I like to be an expert in my own world and I’m definitely not an expert at this.”*^207^ *–* Director of mental health and crisis center |
| **Clinical context of modality choice** | Women and their clinicians felt that the clinical context of a visit should impact modality choice (i.e., in-person, video, or phone). Video allows a visual physical examination (e.g., general appearance and rashes), opportunity for eye contact, ability to pick up on non-verbal cues, and offering of physical direction or instruction (e.g., exercise). An example of when a phone visit worked well was for the assessment of labor. Some women expressed concern about having their concerns fully resolved during a virtual visit, leading to the need for an additional in-person visit. | “*I was also referred by my health visitor for a breastfeeding Zoom call. That was ridiculous. I needed to see someone face-to-face because they have to check your position, your latch and whether your baby has tongue tie. Feeding support has to be there face-to-face and it needs to be available*.”^212^ – New mother |
| **Usability of technology is important** | The evidence was clear that using virtual hardware and software that was easy to use and convenient facilitates the delivery and receipt of virtual care. The amount of prior experience and comfort that the clinician and patient had with the relevant technology facilitated uptake and minimized challenges during set-up and use. | *“The [use of] technology I try to be 20 minutes early for this and I was 10 minutes late, just finding the email, getting in, getting the passwords to work. It’s usually annoying and it was occasionally prohibitive. …..That’s super disappointing so the technology was sort of a drag”*^213^ *–* Perinatal depression telehealth program participant |
| **Existing infrastructure** | Already having infrastructure for virtually-delivered care (e.g., Hardware and software) at the system-, clinician- and patient-level makes uptake of virtual modalities easier. Having options to use multiple modes of technology (e.g., app, video, chat, and phone) during the visit may help meet the needs of the woman. | “*I think it could have been good, if this organization was invested in the equipment. . . It took me four months to get a computer that was a laptop, and I still haven’t been able to crack how to get those two apps on my desktop. . . so I still cannot work remotely*.”^214^ *–* Midwife  “*Well, we already have the machine, and it's already setup going. This is just an extra service that we would have to provide. It's already in place. I mean, IT already knows about the machine, the staff knows how to use it. All we need is the binders and instructions and then do some education and we'd be ready to roll.”*^211^ *–* Emergency Department Nurse on tele-sexual assault nurse examiner consultation |
| ***Adopters*** | | |
| **Negative impact on well-being**  **(high certainty of evidence)** | Women described fatigue and a sense of discomfort or anxiety with seeing themselves on camera and seeing into the private spaces of others. While used to working in groups in person, clinicians reported burnout and isolation. One positive impact was that some clinicians felt lower levels of stress when conducting visits from home. | *"I think people automatically think that working from home is like a glam job, and many of us after now trying it saw how it can be isolating. Because we work quite closely together here just in terms of being in physical contact. And so they found it very isolating, but we had to develop a lot of policies and procedures around supporting our staff."*^207^ *–* Provider of intimate partner violence services |
| **Privacy and safety**  **(moderate certainty of evidence)** | Virtually-delivered care had a *mixed* effect on the patient’s sense of privacy and safety depending on home context. For example, some women appreciated the ability to have an appointment in their own private space as it made them feel more comfortable sharing sensitive information. However, others had difficulty finding an appropriate space that was free from distractions, such as children in the home, or were unable to ensure auditory privacy from children or perpetrators of intimate partner violence. Safety concerns included both online security of the virtual platform and the presence of perpetrators of violence in the home. | “*You do ask them on the phone ‘are you safe at home?’ but I suspect if they’ve got somebody with them who is abusing them…they are going to say yes they’re safe, whereas if they’re on their own face-to-face they might be more able to be honest*”^209^ – Nurse providing tele-abortion care  “…*they might not answer their phone or they might be in the middle of doing other stuff or they take a call while they are in the bath or, you know, something like that and then its noisy and hard to really talk openly and you don’t really get a sense of the full picture as you might if somebody was with you, like you don’t have their full attention*”^215^ – Gynecologist providing menopause care |
| **Translation of existing internal personal skills for women**  **(low certainty of evidence)** | Clinicians had mixed perceptions of women’s engagement in virtual care compared to in-person care with some feeling women were less engaged virtually and others feeling that levels were similar. Women’s own sense of confidence in their abilities to complete the required tasks to participate in telehealth was facilitated by previous use of virtual platforms (e.g., use of Zoom). | “*For some people who have never used it [video-conference delivery] before that could make something feel even more daunting…*”^216^ – Clinical staff member delivering perinatal mood disorder treatment |
| **Translation of existing internal personal skills for clinicians**  **(low certainty of evidence)** | Clinicians who had demonstrated skills related to showing empathy, demonstrating clinical knowledge, and building rapport in-person were often able to translate those skills to virtual settings which facilitated the success of virtually delivered care from the woman’s perspective. | *No quote available* |
| ***Values*** | | |
| **Convenience and cost-savings**  **(high certainty of evidence)** | Virtually-delivered care provides significant convenience and cost savings to women through reducing travel burden, requiring less time away from work, accommodating commitments, and reducing gas and childcare expenses. Women from marginalized groups were most likely to benefit (e.g., women with mobility-limiting disabilities or those with multiple conflicting commitments and limited resources to overcome barriers to care). | “*The fact that [appointments] can be at whichever time is convenient for me and not have to worry about who is going to take me and who is going to pick me up. It takes a weight off my shoulders when the appointments are in this method.”*^206^  *–* Hispanic woman with breast cancer |
| **Clinician continuity**  **(moderate certainty of evidence)** | Women's experience of virtually-delivered care was improved when the clinician providing care was someone with whom the woman already had a previously established relationship or with whom they would be engaging virtually repeatedly over time (e.g., surveillance during breast cancer survivorship care). | *"I mean I can remember [cancer nurse] right from the first time when they diagnosed my cancer, which was quite a shock…She always had the time just to talk you through it and I found her very reassuring right from the start so and I think it's the continuity. I know she came to see me in the hospital and she spoke to me between."*^217^ *–* Woman with breast cancer receiving cancer follow up  *"You see so many different people as well and you don't really, very often see the same person twice, that I think is quite off putting. Everybody's very nice, they say 'I'm so and so' and immediately it goes straight out of my head and I think well probably my name goes out of theirs because they see so many people."*^218^ *–* Woman with ovarian cancer receiving telephone nurse-led follow up |
| **Loss of collateral benefits**  **(low certainty of evidence)** | Some women missed aspects of care received during in-person visits, such as interpersonal interactions with members of the extended clinical team (e.g., intake nurses and front desk staff) and with other patients. | "*because you're on Zoom, and it's harder to make personal connections, to know a person in person, that you could talk to." - virtual group participant”^219^ –* Virtual group participant  *"I had hopes that there would be more interaction with the group and not just the singular like the instructor structure."*^213^ *–* Woman participating in virtual group for perinatal depression |
| **Modality choice**  **(high certainty of evidence)** | Women expressed a clear consensus that no modality is right for every woman in every situation and that clinics should offer patients autonomy around selection of visit modality (e.g., in-person vs virtual). Being able to choose the visit modality was highly valued by women and seen by some as manifestation of delivering patient-centered care. | *"It was important to patients to be able to choose if and when they wanted to use [virtual care] given the multitude of shifting variables that modulated whether it was preferable or convenient for them.”*^220^ *–* Woman receiving cancer follow up |
| ***Organization*** | | |
| **Organizational culture** | Normalizing technological problems of virtually delivered care, providing initial and ongoing information, and technology and leadership support promoted uptake of virtual care. Staff champions were also helpful. | *“[a specific staff member] was the one person in that whole entire clinic that got it and thought it was an important service. So that was their culture, “We don’t really know what this is and we don’t really think it’s important”*^221^ *–* Telehealth team trying to implement a women’s reproductive behavioral health telemedicine program |
| **Need for outreach** | Women and their clinicians felt that organizational outreach about virtually-delivered care options was important as inequitable awareness of this resource could lead to worsening racial and socioeconomic disparities. Importantly, this outreach should consider varying levels of digital literacy and be in several languages. | *“Even just getting it out there, that you can talk about your birth control via telehealth … some people just fell off the map period. I mean we tried to reach out to them, but a lot of our patients don’t have working numbers, or they don’t necessarily answer their phone, … How to just make it known, you could call here?”*^222^ – Clinician in tele-contraception clinic |
| **Required resources** | Clinicians noted that efficient and effective use of virtual care necessitated sufficient clinic staffing to support virtual care in real-time. Adequate physical space for conducting virtual visits and clear and consistent communication between patients and clinics about use of virtual modalities were also cited as important. | *No quote available* |
| **Importance of workflow processes** | Clinicians emphasized that the clinic workflow had to incorporate the realities of virtual care and how they differ from in-person care workflow. This included policies to address issues such as privacy and safety and balancing multiple modality types within a clinic. | “*The community outpatient clinics had real concerns [about telemental health safety]. We issued policies, we did violence prevention training, we met with local law enforcement. It’s been fine, it’s been remarkably good.”*^223^ – Clinic mental health director |
| ***Wider System*** | | |
| **Clinician and clinical deserts**  **(moderate certainty of evidence)** | Living in areas with limited or no access to a specific women's health service facilitated the use of virtually-delivered care. For example, this was the case for time-sensitive, women-specific care needs such as pregnancy, abortion care, or sexual assault nurse examinations. Access could be determined by rurality, travel limitations, or competing demands that prevented access to available in person services. In addition, clinical deserts (or geographic regions without adequate presence of certain clinician types) may be particularly important for women with key intersectional identities (e.g., IPV services for LGBT, women with a disability, or women preferring a race-concordant clinician) and those living in harsher climates. | "*We live in a rural area and there's not a whole lot around here as far as breastfeeding support, so it was nice to have something available and be able to use it and not have to leave the house and travel to get somewhere to get that support."*^224^ *–* Primiparous mother receiving tele lactation visits  *‘I feel like it’s vastly increased our access to the women that are most vulnerable. You know, our wealthier patients will get whatever they need, regardless of telemedicine, but in rural areas it’s a lot more difficult.*”^225^ *–* Telemedicine abortion clinic staff in Alaska |
| **Regional limitations** | Technology resource availability varied across communities which influenced adoption; this variability was primarily noted in rural areas and largely focused on internet access. Technology issues included unreliable internet connectivity, limited bandwidth, the digital divide and limited cell phone data. | *“Like rural or more remote people, it's been a bit mixed in the sense that we have had some people who are much more able to participate because it's remote and they don't have to travel a significant distance......but we do also have people who just don't have the internet connection or even the reliable phone connection to participate effectively. It's kind of an insolvable barrier for us. When people don't have the internet to do it, it's really hard to get them into the programs to start with.*”^219^ – Community stakeholder for cancer survivorship program |
| **Payment structure** | The mechanism and amount by which virtually-delivered care was funded or reimbursed influenced its uptake and adoption. For example, reimbursement rates similar to face-to-face appointments influenced sustainability of virtually-delivered care. | *“So there are many complex issues and trauma experienced by these patients [who experience domestic violence] who are absolutely going to take more than 10 minutes, and that I would be unable to claim any compensation for that extra time spent with the patient…So, frankly, the main barrier I think is more the business model as opposed to having reliable technology or acceptance* *[of virtual care] by patients.”*^207^ – Domestic violence and sexual assault clinician |
| ***Embedding and adaptation over time*** | | |
| **Need for strategies to sustain over time** | Organizations need to plan for and execute strategies that support and maintain virtual services as the healthcare system context and user preferences evolve over time. | *No quote available* |

IPV: intimate partner violence, NASSS: non-adoption, abandonment, scale-up, spread, sustainability framewor
